# Prioritization of Eleven-Nineteen-Leukemia Inhibitors as Orally Available Drug Candidates for Acute Myeloid Leukemia

**DOI:** 10.1021/acs.jmedchem.4c01337

**Published:** 2024-11-12

**Authors:** Xuejiao Shirley Guo, Sandeep Atla, Satyanarayana Nyalata, Yugendar R. Alugubelli, Peng-Hsun Chase Chen, Shiqing Xu, Wenshe Ray Liu

**Affiliations:** † Texas A&M Drug Discovery Center and Department of Chemistry, Texas A&M University, College Station, Texas 77843, United States; ‡ Department of Pharmaceutical Sciences, Irma Lerma Rangel College of Pharmacy, Texas A&M University, College Station, Texas 77843, United States; § Institute of Biosciences and Technology and Department of Translational Medical Sciences, College of Medicine, Texas A&M University, Houston, Texas 77030, United States; ∥ Department of Biochemistry and Biophysics, Texas A&M University, College Station, Texas 77843, United States; ⊥ Department of Cell Biology and Genetics, College of Medicine, Texas A&M University, Bryan, Texas 77807, United States

## Abstract

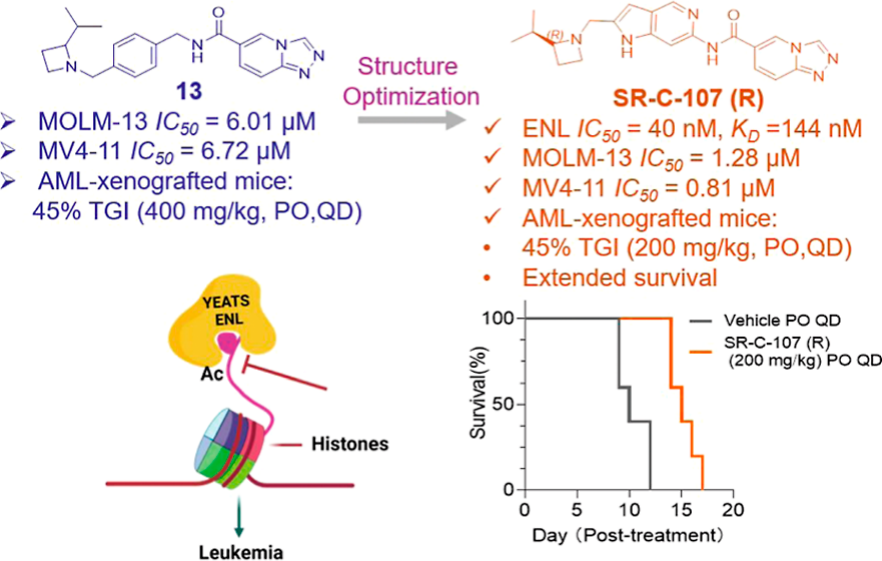

Acute myeloid leukemia (AML) is the second most prevalent and fatal form of leukemia. The growth of AML cells harboring oncogenic MLL rearrangements relies on the YEATS domain-containing protein ENL. Many small molecule inhibitors targeting ENL have been developed. To prioritize these inhibitors for in vivo studies, a NanoBRET system was introduced to evaluate their cellular permeability and potency. This screening identified inhibitor **13** as a promising candidate. This inhibitor has remarkable metabolic stability and potent antiproliferative effects on MLL-fusion leukemia cell lines. In AML-xenografted mice, inhibitor **13** significantly improved survival. Subsequent optimization efforts led to the development of **SR-C-107 (R)**, which exhibited strong activity against AML both at the cellular level (*CC*_50 (MOLM-13)_: 1.25 ± 0.18 μM; *CC*_50 (MV4-11)_: 0.81 ± 0.15 μM) and in vivo. These findings establish **SR-C-107 (R)** as a compelling candidate for AML treatment and lay the groundwork for the development of next-generation AML inhibitors.

## Introduction

Acute myeloid leukemia (AML) is the second most frequently diagnosed leukemia in both adults and children, and it is the most lethal subtype among adults.^1^ Unlike chronic leukemia, AML progresses rapidly, often requiring immediate intervention.^2^ Despite this urgency, the 5 year survival rate remains as a dismal 25%, accounting for nearly half of all leukemia deaths.^3^ AML is characterized by the uncontrolled proliferation of abnormal myeloblasts, which impairs the production of normal blood components, including mature red blood cells, neutrophils, monocytes, and platelets.^4,5^ Genetic anomalies, such as gene mutations, chromosomal rearrangements, and altered expression patterns of various genes and microRNAs, are often implicated in AML.^6^ Notably, nonrandom chromosomal translocations are a major drive of these gene rearrangements, with up to 749 chromosomal aberrations associated with AML.^6,7^ Among these, arrangements involving the mixed-lineage leukemia gene (MLL or KMT2A) at the 11q23 locus are common, occurring in roughly 5 to 10% of AML cases.^8^ Mutation in MLL are also frequently linked to a poor prognosis.^9^

**Figure 1:**
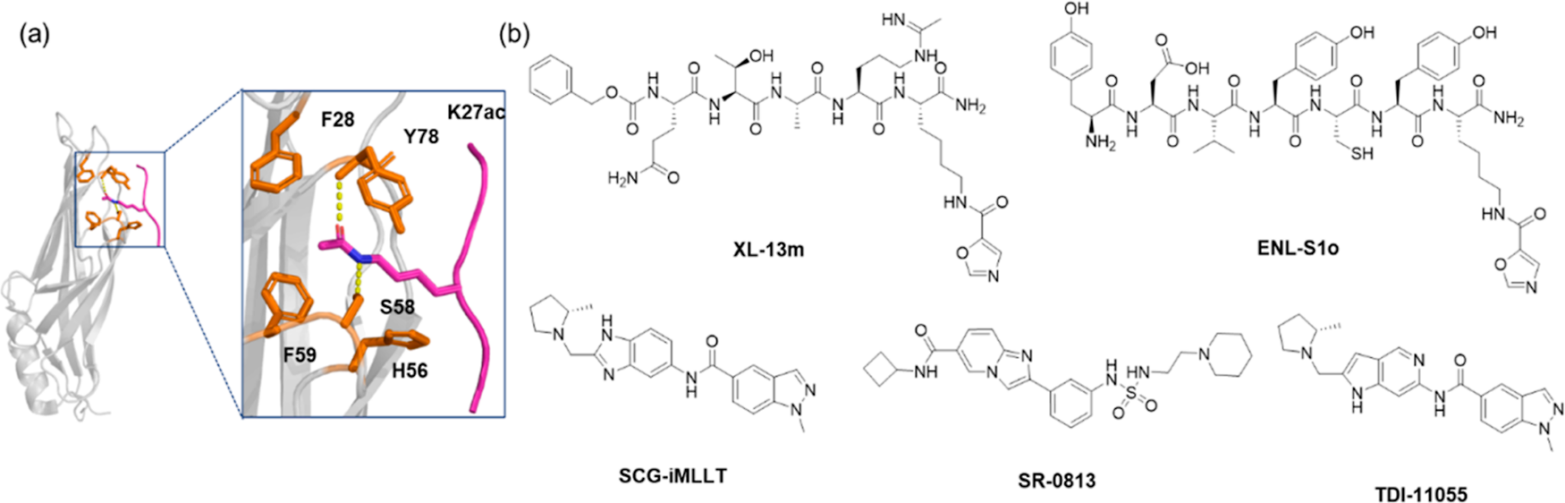
ENL YEATs domain and its representative inhibitors. (a) The structure of the ENL YEATS domain bound to an H3K9ac peptide, derived from PDB entry 5J9S. The ENL YEATS domain is depicted in gray in cartoon representation. Key residues that form the acetyl-lysine (Kac) binding pocket are shown in stick representation and highlighted in orange. The K3K9ac ligand is represented in hot pink, with the Kac moiety displayed in stick form. Two hydrogen bonds between Kac and residues S58 and Y78 are highlighted in yellow. (b) Representative chemical probes for the ENL YEATS domain in the literature.

So far, over 80 genes have been identified as fusion partners with MLL, leading to the production of chimeric proteins that combine the N-terminal region of MLL with the C-terminal segment of the fusion partner.^10−13^ The five most common MLL-translocation events in AML are MLL-AF4 (t(4; 11)(q21; q23)), MLL-AF9 (t(9; 11)(p22; q23)), MLL-ENL (t(11; 19)(q23; p13.3)), MLL-AF10 (t(10; 11)(p12; q23)), and MLL-AF6 (t(6; 11)(q27; q23)).^14^ Among MLL-fusion partners, AF9 and ENL are homologous proteins sharing the evolutionarily conserved YEATS domain, named after its founding members: Yaf9, ENL, AF9, Taf14, and Sas5.^15^ This domain acts as an epigenetic reader, recognizing posttranslational modifications in histones including lysine acetylation and lysine crotonylation to exert chromatin remodeling, transcriptional regulation and histone modification.^16−20^ Both AF9 and ENL play key roles in several transcriptional elongation complexes, including the super elongation complex (SEC) as illustrated in Figure S1.^21^ Their fusion to MLL drives leukemogenesis via activation of transcription of critical genes such as HOX, MEIS, and MYC.^22−24^ While AF9 and ENL are components of the SEC/DOT1L transcriptional assembly, recent studies highlight ENL, rather than AF9, as a crucial stabilizer of the complex, ensuring its binding to DNA and promoting dysregulated gene transcriptions necessary for leukemogenesis. Importantly, disrupting the interaction between the ENL YEATS domain and acetylated histones or reducing ENL protein levels impairs leukemia progression with minimal impact on normal hematopoietic stem and progenitor cells, positioning ENL as a potential therapeutic target for AML.^25,26^ The ENL YEATS domain features a long, narrow hydrophobic “open-end” epigenetic reader pocket as shown in Figure 1a, making a feasible target for small molecules and peptides, as demonstrated by recent studies that introduced such inhibitors.^26^ However, maintaining high selectivity for the ENL YEATS domain over its close homologue, the AF9 YEATS domain, remains a considerable challenge in developing these inhibitors.^27−30^

Various ENL inhibitors with distinct chemical scaffolds have been identified. A peptide-based inhibitor, XL-13m, was designed with an expanded π system to enhance π-stacking interactions with the aromatic triad in the Kac pocket.^31^ Using a phage-assisted active site-directed ligand evolution (PADLE) technique, Chen et al. developed a selective peptide inhibitor ENL-S1o, which boasts a *K*_D_ value of 2.0 nM and shows 28-fold selectivity for the ENL YEATS domain over AF9 YEATS.^32^ Moustakim et al. were the first to report small molecule ENL YEATS inhibitors, among which SGC-iMLLT (*K*_D_: 129 nM) features a benzimidazole-amide scaffold.^33^ More recently, SR-0813 (*K*_D_: 30 nM), which incorporates an amido-imidazopyridine scaffold, was developed using sulfur(VI) fluoride exchange (SuFEx)-based high-throughput medicinal chemistry.^34^ However, SR-0813’s rapid metabolic degradation in mouse liver microsomes (half-life of 9.3 min) limits its potential as an in vivo candidate. Another recent advancement was the development of TDI-11055, an orally available ENL YEATS inhibitor that replaces the benzimidazole moiety in SGC-iMLLT with a pyrrolopyridine group and demonstrates improved efficacy both in vitro and in vivo.^35,36^ Although these compounds effectively highlight the therapeutic potential of targeting the ENL YEATS domain, there remains considerable room for improving their cellular/animal potency and pharmacokinetic (PK) properties.

## Results and Discussion

### Design, Preparation, and Validation of a NanoBRET System for the Evaluation of ENL YEATS Domain Inhibitors

**Figure 2:**
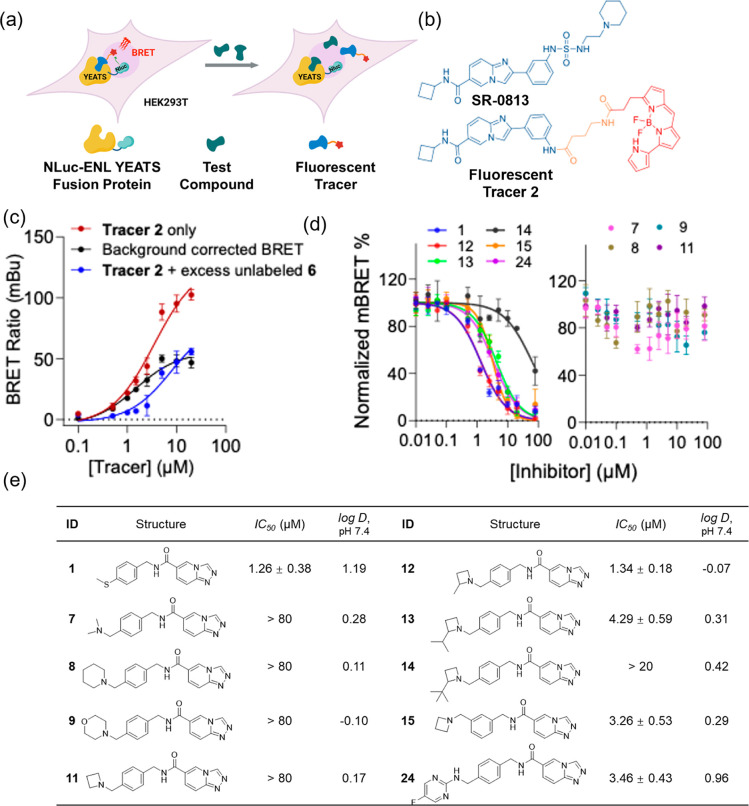
ENL-NanoBRET assay and its applications in assessing cellular potencies of ENL inhibitors. (a) Schematic representation of intracellular target engagement in a designed ENL-NanoBRET assay. A cell-permeable fluorescence tracer binds dynamically to its intracellular target protein, ENL YEATS, which is fused to NLuc, resulting in a measurable BRET signal. (b) Structure of SR-0813 and its derived **Tracer 2**, in which the SR-0813 core and the BODIPY 590 moiety are shown in blue and red, respectively, with the 4-aminobutyric acid linker depicted in orange. (c) Apparent affinity of **Tracer 2** for the NLuc-ENL YEATS fusion protein in HEK293T cells. (d) Normalized NanoBRET signal response of ENL inhibitors in HEK293T cells expressing NLuc-ENL YEATS. (e) *IC*_50_ and *log*D values, along with the structures of corresponding inhibitors are presented as the mean ± SD, *n* = 3.

In one of our previous studies, we reported multiple ENL YEATS domain inhibitors.^37^ To prioritize these inhibitors for in vivo tests, we proceeded to characterize them further. Recent studies have highlighted the potential of NanoBRET in probing the direct interactions between small molecules and intracellular targets, offering a way to confirm that cellular efficacy is due to interactions between a molecule and its target protein within the cell.^38^ For AML inhibitors, the traditional evaluation of cellular antiproliferation effects typically requires long incubation periods (one to 2 weeks), resulting in delayed results and less accurate assessments of the cellular permeability of these inhibitors. This extended timeline can also hinder the rapid development of new molecules. In contrast, the NanoBRET assay, which only requires 2 h incubation, allows for the quick elimination of compounds with low intracellular target engagement. This significantly accelerates the drug development process and provides a more accurate platform for evaluating cellular permeability. With this in mind, we developed an ENL-NanoBRET system. As shown in Figure 2a, the NanoBRET assay consists of two main components: a target protein expressed as a fusion with NLuc and a cell-permeable fluorescent tracer with specific affinity for the target protein. In our design, the ENL YEATS domain was genetically fused to the C-terminus of NLuc, and the fusion protein is recombinantly expressed in HEK293T cells. Due to the small size of the ENL YEATS domain (∼16 kDa), the active sites of NLuc and the ENL YEATS protein are in close proximity. When a fluorescent tracer binds to the ENL YEATS domain, it aligns closely with furimazine, which binds to NLuc, generating a strong BRET signal. This BRET signal will diminish when a test compound binds to the ENL YEATS protein. Upon the binding of a test compound to the ENL YEATS domain, the tracer is displaced, reducing the BRET signal, as illustrated in Figure 2a. To address the batch-to-batch inconsistencies previously observed with transiently expressed NanoBRET constructs,^39^ we developed HEK293T cell lines with stable NLuc-ENL YEATS expression (Figure S4). This approach ensures greater consistency in the ENL-NanoBRET assay.

Initially, we designed NanoBRET **Tracer 1** as shown in Scheme 3 based on inhibitor **1**, by coupling the widely utilized dye BODIPY590 to inhibitor **1** via a butyl linker. The intracellular binding capacity of this tracer to the ENL YEATS domain was then evaluated. However, **Tracer 1** exhibited a limited affinity profile for the NLuc-ENL YEATS fusion protein (Figure S5a), resulting in low background-corrected BRET values, which restricts its further use. Inspired by SR-0813 (Figure 2b, top), we designed a cell-permeable fluorescent **Tracer 2** (Figure 2b, bottom) to serve as a NanoBRET acceptor. This was achieved by tethering BODIPY590 (highlighted in red) to the core structure of SR-0813 (highlighted in blue) via a butyl chain (shown in orange) using two sequential amidation reactions. Subsequent characterization showed a concentration-dependent, saturable BRET signal curve for **Tracer 2** (Figure 2c). To refine the BRET data, nonspecific binding signal was removed by coincubating **Tracer 2** with 80 μM of unlabeled compound **20** (Scheme 3). After refinement, **Tracer 2** yielded an *EC*_50_ value of 1.46 μM. As with other competitive binding assays, the concentration of the tracer in the NanoBRET assay impacts the apparent affinity of competing compounds for the ENL YEATS domain. Both the tracer and test compounds compete for binding to the NLuc-ENL YEATS protein, and higher tracer concentrations can increase apparent *IC*_50_ values of tested compounds, as described by the Cheng–Prusoff relationship.^40^ To optimize the tracer concentration for the ENL-NanoBRET assay, we measured the *IC*_50_ value of inhibitor **24** for the ENL YEATS domain in a competitive assay using varying concentrations of **Tracer 2** (Figure S5b). The assay quality was determined by comparing the raw fold change in the BRET ratio at 1 μM of **Tracer 2** with the BRET ratio in the presence of a saturating dose of unlabeled inhibitor **6**. This defined the assay window (value: 3.59) and demonstrated a high *Z* factor (value: 0.62). Ultimately, 1 μM was selected as the optimal **Tracer 2** concentration for the ENL-NanoBRET assay.

**Scheme 1:**
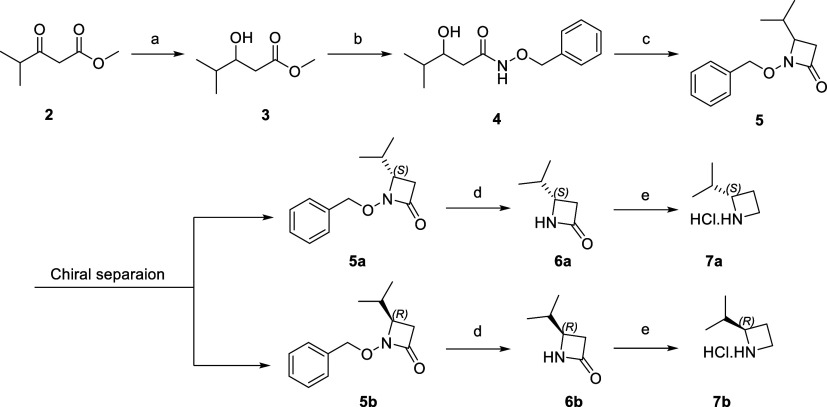
Synthesis of Intermediates for Two Enantiomers of Inhibitor **13** and **SR-C-107** Reagents and conditions: (a) NaBH_4_, MeOH, −10 °C, 30 min; (b) OBHA*HCl, Al (CH_3_)_3_, DCM, 0 °C—rt, 16 h; (c) TPP, CCl_4_, TEA, ACN, 0 °C—rt, 24 h; (d) Raney Ni, MeOH, rt, 16 h; (e) LAH, TMS-Cl, THF, 0 °C—rt, 48 h, 2 M HCl.

**Scheme 2:**
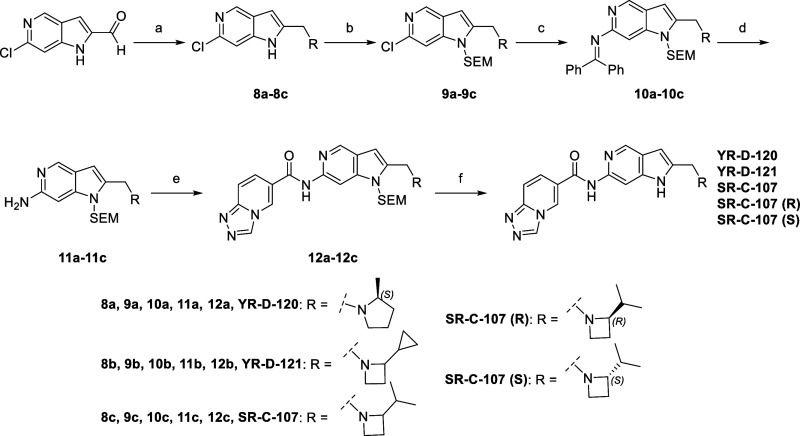
Synthesis of Target Compounds Reagents and conditions: (a) R = amine, NaBH(OAc)_3_, DCE, cat. AcOH, 16 h; (b) SEM-Cl, NaH, 0 °C-rt, 2 h; (c) BINAP/Pd_2_(dba)_3_, NaOtBu, toluene, 110 °C, 4 h; (d) HCl, THF/H_2_O, 16 h; (e) [1,2,4]triazolo[4,3-a]pyridine-6-carboxylic acid, EDCI, pyridine, 50 °C, 12 h; (f) TFA, DCM, 12 h.

With optimized assay conditions in place, we conducted ENL-NanoBRET assays on a selection of previous developed ENL YEATS inhibitors (inhibitors **1**, **7–9**, **11–15** and **24**). This involved coincubating various concentrations of an inhibitor with 1 μM of **Tracer 2** in HEK293T cells stably expressing NLuc-ENL YEATS for 2 h. Among reported inhibitors, **1** and **12** showed significant reduction in the BRET signal, with *IC*_50_ values of 1.26 and 1.34 μM, respectively, indicating strong cellular permeability and high potency. **13**, **15**, and **24** exhibited comparable inhibition potencies, with *IC*_50_ values of 4.29, 3.26, and 3.46 μM, respectively. Interestingly, inhibitor **7**, which previously showed strong potency in inhibiting MV4-11 and MOLM-13 cells over 6 day incubation, did not demonstrate high potency in the 2 h NanoBRET assay.^37^ This is likely due to its low cellular permeability, contributing to its potency over extended incubation but reducing its efficacy in shorter assays. The positive charge on inhibitor **7** may also cause it to accumulate in cells during longer incubation periods. Since our goal was to identify inhibitors with high cellular permeability for further improvement, we did not prioritize **7** further for optimization. Our findings for the other inhibitors were consistent with previously published in vitro biochemical affinity results.^37^ Notably, inhibitor **14**, despite being analogous to inhibitor **13**, displayed significantly lower cellular inhibition potency toward ENL YEATS, with an *IC*_50_ exceeding 20 μM (Figure 2d, left). This was further supported by its weak activity in MOLM-13 cells, with only ∼30% growth inhibition at 64 μM (Figure S8f). Additionally, while **8**, **9**, and **11** showed strong potency in the in vitro AlphaScreen assay, they exhibited much lower potencies in the ENL-NanoBRET assay. As seen in Figure 2d, these inhibitors produced minimal reductions in NanoBRET signal, even at concentrations up to 80 μM. The relatively low partition coefficients between *n*-octanol and PBS (pH 7.4) for these inhibitors (Figure 2e) suggest poor cellular permeability and possible active efflux.

### Structure–Activity Relationship Exploration of Inhibitor **13**

**Figure 3:**
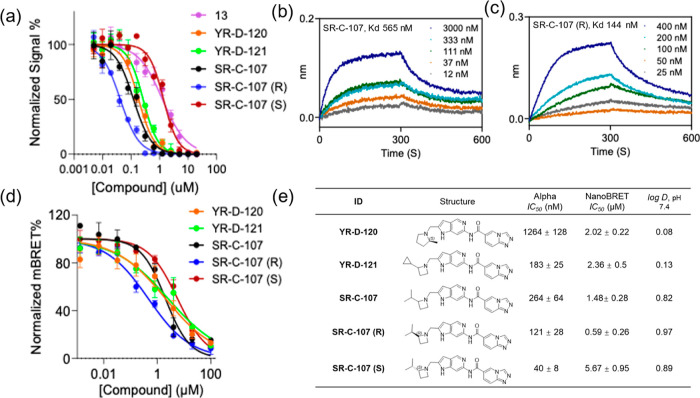
Newly developed inhibitors and their in vitro characteristics. (a) AlphaScreen analysis (top) showing the inhibition of ENL YEATS binding to the ENL-S1 peptide by various molecules. *IC*_50_ values for corresponding inhibitor structures are given as the mean ± SD, *n* = 3 (bottom). Binding kinetics of (b) **SR-C-107** (top) and (**c**) **SR-C-107 (R)** (bottom) to the ENL YEATS domain were characterized using BLI with varying concentrations of inhibitors. (d) Normalized NanoBRET signal response of newly developed ENL inhibitors in HEK293T cells expressing NLuc-ENL YEATS. (e) Determined characteristics of newly developed ENL inhibitors.

Inhibitor **13** emerged as one of four inhibitors displaying strong potency in the ENL-nanoBRET assay. Given its demonstrated in vivo efficacy, as we will discuss later, we proceeded with its structure–activity relationship exploration. Our goal was to rapidly optimize inhibitor **13** to enhance its drug-like properties for in vivo application, while preserving its potent and stable profile. As discussed previously,^37^ inhibitor **13** likely binds to the ENL YEATS domain in a manner similar to the interaction between the Kac group in a native histone peptide and the ENL YEATS Kac binding channel, forming hydrogen bonds with Y78 and S58. However, the relatively flat surface on both sides of the ENL YEATS Kac binding channel limits the space available for chemical modification of inhibitor **13**.^37^ Inspired by the successful conversion of SGC-iMLLT to TDI-11055, where a nitrogen atom was introduced into the ring system and one of the benzimidazole nitrogen atoms was removed,^35^ we applied a similar approach to optimize inhibitor **13**. By replacing the benzyl ring with 1*H*-pyrrolo[3,2-c]pyridine, we developed **SR-C-107**. This modification was designed to enhance hydrogen bond interactions and predispose the molecule to adopt the conformation needed for binding to the ENL YEATS domain. Additionally, the nitrogen atom in the azetidine ring of **SR-C-107** is positioned to potentially form a salt bridge with the Glu75 side chain, mirroring the interaction of the pyrrolidine nitrogen atom in SGC-iMLLT. To further investigate the role of the 2-isopropyl azetidine group in binding affinity, we developed **YR-D-120** and **YR-D-121**. Synthetic routes for these molecules are outlined in Scheme 2. The inhibition potency of three compounds toward the ENL YEATS domain was evaluated by an AlphaScreen assay. Instead of a commonly used biotinylated H3K27cr peptide (*K*_D, ENL_ = 34 μM), we employed a recently developed peptide, ENL-S1, which has much higher affinity for ENL (*K*_D, ENL_ = 36.3 nM).^41^ This peptide was biotinylated and, together with a his-tagged ENL YEATS protein, Ni-NTA, and streptavidin α beads, was used to screen inhibitors.^32^ As shown in in Figure 3a, all newly designed inhibitors (**YR-D-120**, **YR-D-121**, and **SR-C-107**) displayed significantly lower *IC*_50_ values compared to inhibitor **13** (*IC*_50_: 1264 ± 128 nM), with *IC*_50_ values of 183 ± 25, 264 ± 64 and 121 ± 28 nM, respectively. Since **SR-C-107** possesses a chiral center and was synthesized as a racemate, it is likely that its S and R isomers exhibit different potency, stability, half-life, clearance and bioavailability. To investigate this, we synthesized the two isomers by restarting the synthesis and performing chiral separation of two enantiomers of an intermediate molecule 1-(benzyloxy)-4-isopropylazetidin-2-one. The two isomers were successfully obtained and confirmed via X-ray diffraction analysis (Scheme 1, Figures S43 and S44). They were then used to generate **SR-C-107 (R)** and **SR-C-107 (S)** (Schemes 1 and 2). *IC*_50_ tests of the two products showed that **SR-C-107 (R)** demonstrated superior inhibitory activity, with an *IC*_50_ value of 40 nM, compared to **SR-C-107 (S)**, which had an *IC*_50_ of 1380 nM. Two isomers of inhibitor **13** were synthesized similarly. However, given the minor difference in *IC*_50_ values between inhibitor **13** (1264 nM, Figure 3a), and its isomer **13 (S)** (970 nM, Figure S2), as well as the challenges in separating these isomers, we opted to continue using the racemate of inhibitor **13** for subsequent studies. We also assessed binding affinities of developed inhibitors to the ENL YEATS domain using biolayer interferometry (BLI). Biotinylated-ENL YEATS was immobilized onto SSA biosensors to measure the binding kinetics of tested molecules at various concentrations. Results, as shown in Figure 3b,c, indicated that **SR-C-107 (R)**, binds strongly to the ENL YEATS domain, with a dissociation constant (*K*_D_) of 144 nM. In contrast, **SR-C-107** exhibits a weaker affinity, with a *K*_D_ value of 565 nM. Weaker binding was also observed for **YR-D-120** and **YR-D-121** with *K*_D_ values of 645 and 1342 nM, respectively (Figure S3). These findings align with their respective *IC*_50_ values determined in the AlphaScreen assay.

### NanoBRET Analysis of Newly Developed Inhibitors

Using the developed ENL-NanoBRET assay, we characterized the three newly synthesized inhibitors and the two enantiomers of **SR-C-107** as well. Among the tested compounds, **SR-C-107 (R)** showed the best potency, with an *IC*_50_ value of 0.59 μM, indicating its excellent cellular permeability and engagement with the ENL YEATS active site (Figure 3d). In contrast, the racemate **SR-C-107** and its S-isomer showed reduced cellular engagement, with *IC*_50_ values as 1.48 and 5.67 μM, respectively. These results are consistent with their corresponding in vitro inhibition activities. The comparable cellular permeability of the two isomers is supported by their similar partition coefficient (**SR-C-107 (R)***log* D: 0.97; **SR-C-107 (S)***log*D: 0.89; Figure 2e). Although **YR-D-120** and **YR-D-121** displayed strong in vitro ENL YEATS binding affinity, their cellular inhibition potencies detected by NanoBRET were moderately lower, with *IC*_50_ values of 2.02 and 2.36 μM, respectively (Figure 2e), this reduction in potency could be attributed to their relatively low lipophilicity (**YR-D-120***log* D: 0.08; **YR-D-121***log* D: 0.13). Based on all NanoBRET results, we prioriitized compounds **1**, **12**, **13**, **15**, **24**, **YR-D-120**, **YR-D-121**, **SR-C-107**, **SR-C-107 (R)**, and **SR-C-107 (S)** for further characterizations.

### Metabolic Stability in Human Plasma and Liver Microsomes

**Figure 4:**
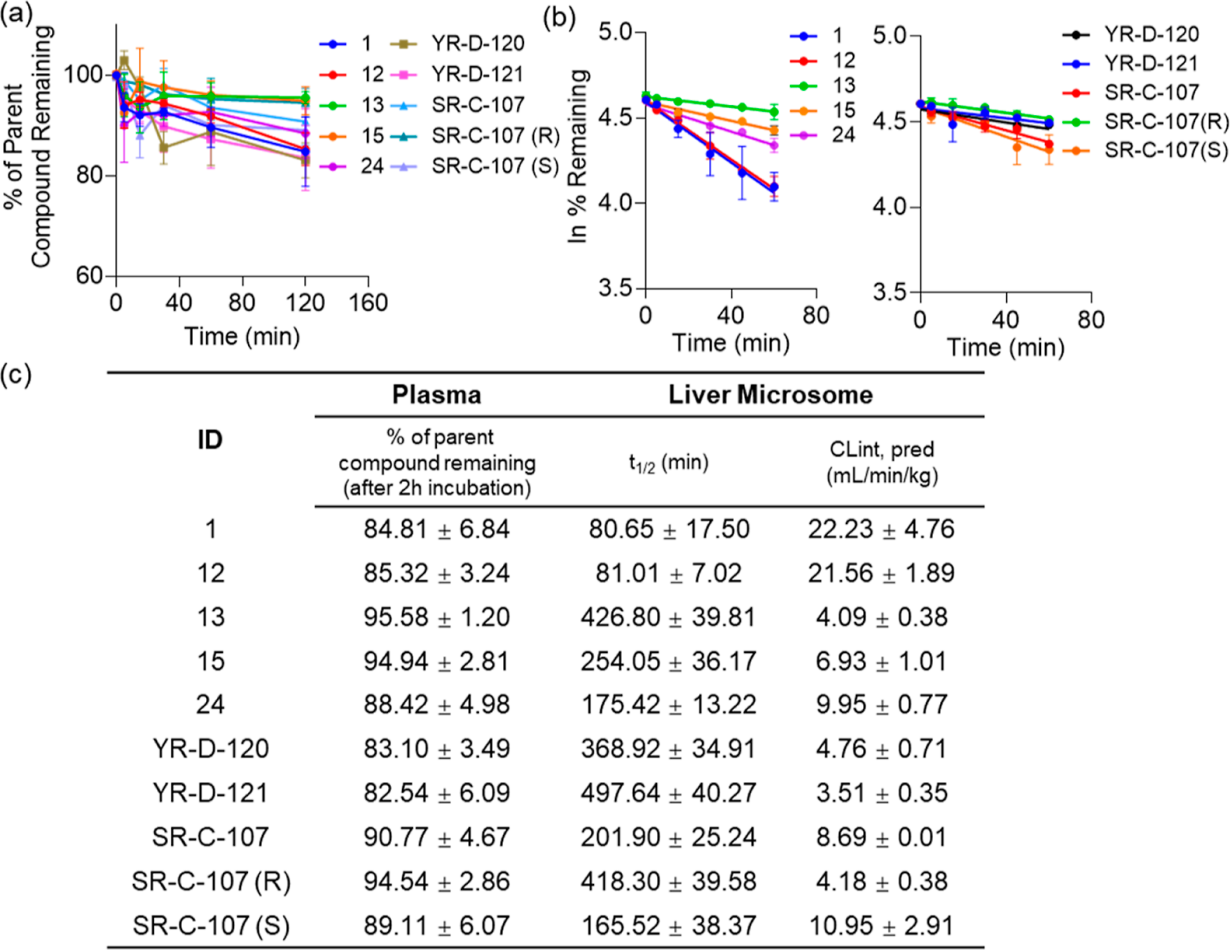
Metabolic stability of ENL inhibitors. Tests done in (a) human plasma and (b) HLM. (c) Corresponding parameters given as the mean ± SD, *n* = 3.

To assess the potential of our prioritized inhibitors for in vivo applications, we conducted a study of their in vitro metabolic properties. Plasma stability testing is a key component of in vitro ADME (absorption, distribution, metabolism, and excretion) screening assays, used to gauge the stability of drug candidates in plasma.^42^ The efficacy of a drug in vivo is generally contingent upon its slow degradation rate in plasma.^43^ As shown in Figure 4a, we evaluated the stability of indicated inhibitors in human plasma. Inhibitors **13**, **15** and **SR-C-107 (R)** exhibited minimal degradation after 2 h incubation in human plasma at 37 °C, retaining approximately 95% of their original concentration in plasma, indicatin high stability (Figure 4c). **SR-C-107 (S)** showed lower plasma stability compared to **SR-C-107 (R)**. Due to this, **SR-C-107** showed quicker elimination than **SR-C-107 (R)**, with 90.77% of **SR-C-107** remaining following 2 h incubation in human plasma (Figure 4c). In contrast, **1**, **12**, **24**, **YR-D-120**, and **YR-D-121** displayed reduced stability in human plasma, with approximately 15% degradation after 2 h incubation (Figure 4a,c).

Hepatic metabolism is a primary pathway for drug elimination. In vitro studies of hepatic metabolism are often conducted using the microsomal incubation technique to determine the intrinsic clearance rate (*CL*_int_).^44^ This assay is essential in drug development as it allows for the screening of lead compounds from a broad array of candidates with similar in vitro potency.^45^ To prioritize our ENL inhibitors, we conducted this assay by incubating each inhibitor with human liver microsomes (HLM), either with or without nicotinamide adenine dinucleotide phosphate (NADPH), the cofactor for flavin monooxygenase-dependent oxidation, at 37 °C. Degradation levels were quantified using liquid chromatography–tandem mass spectrometry (LC–MS/MS). As shown in Figure 4b, the metabolic stability of each inhibitor was determined by plotting the natural logarithm (Ln) of the remaining inhibitor concentration (*y* axis) against incubation time (*x* axis). The slope of this plot ws used to calculate the metabolic rate of each inhibitor, which was then used to determine the in vitro half-life (*t*_1/2_) and intrinsic clearance rate. Results are presented in Figure 4c. All compounds were stable in HLM in the absence of NAPDH (Figure S6). In the presence of NADPH, our results showed that inhibitors **1** and **12** degraded rapidly, with only about 60% remaining after 60 min incubation. In contrast, inhibitor **24** showed moderate degradation but maintained an acceptable intrinsic clearance rate (*CL*_int_ = 9.95 mL/min/kg). Meanwhile, **13**, **15**, **YR-D-120** and **YR-D-121** displayed excellent microsomal stability, with over 90% of parent compounds remaining post 1 h incubation. Their determined intrinsic clearance rates are 4.09, 6.93, 4.76, and 3.51 mL/min/kg, respectively, highlighting their promising metabolic stability profiles. For two enantiomers of **Sr-B-107**, the more potent **SR-C-107 (R)**, with a half-life of 418 min and low clearance rate of 4.18 mL/min/kg, demonstrating superior metabolic durability than **SR-C-107 (S)** (*t*_1/2_ = 165 min, *CL*_int_ = 10.95 mL/min/kg), further supporting its potential for AML treatment.

### In Vitro Anti-Tumor Efficacy of ENL Inhibitors

**Figure 5:**
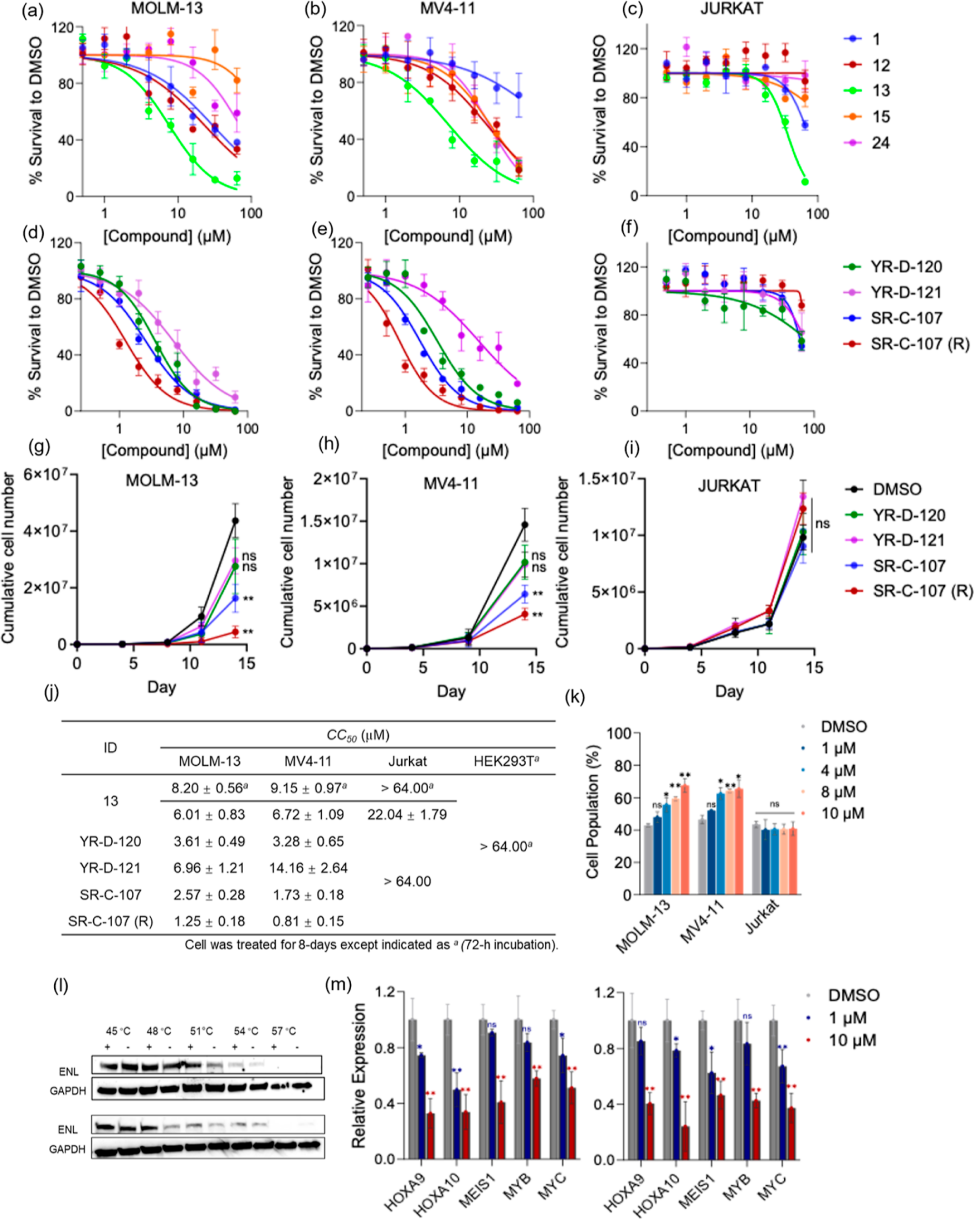
In vitro antitumor potencies of ENL inhibitors. (a) MOLM-13, (b) MV4-11, and (c) Jurkat cell viability post 8 day treatment with previously reported ENL inhibitors. (d) MOLM-13, (e) MV4-11 and (f) Jurkat cell viability post 8 day treatment with newly designed ENL inhibitors. Proliferation of (g) MOLM-13, (h) MV4-11, and (i) Jurkat cells in the presence of 1 μM of ENL inhibitors. (j) Half-maximum cytotoxic concentration (*CC*_50_) values for all compounds across each cell line are shown (*n* = 3). (k) Comparison of the percentage of cells in the G1 phase in various cell lines following 72 h treatment with different concentration of **SR-C-107 (R)**. (l) CETSAs of ENL in MOLM-13 (top) and MV4-11 (bottom) cells treated with 10 μM of **SR-C-107 (R)** (+) or DMSO control (−) at indicated temperatures, with GAPDH as a loading control. (m) qRT-PCR analysis of HOXA9, HOXA10, MEIS1, MYB, and MYC gene expression in MOLM-13 (left) and MV4-11 (right) cells post 72 h treatment with **SR-C-107 (R)** or the DMSO negative control. *P < 0.05, **P < 0.01, not significant (n.s.) *P* > 0.05.

After evaluating the in vitro metabolic stability of inhibitors, we shifted focus on characterizing their efficacy in targeting ENL-dependent tumor cells. We employed two cell lines, MV4-11 and MOLM-13, which are sensitive to ENL depletion, along with Jurkat cells that are insensitive to the loss of ENL, for cellular assessment.^25^ Additionally, HEK293T cells served as a nontumorous control for comparison. We initiated our study by assessing the viability of four selected cell lines following 72 h treatment with previously reported inhibitors **1**, **12**, **13**, **15**, and **24**. Notable cytotoxic effects were observed in MOLM-13 and MV4-11 (Figure S7a,b) cells, particularly with inhibitor **13**, which exhibited the most significant cytotoxicity, with *CC*_50_ values of 8.20 μM for MOLM-13 cells and 9.15 μM for MV4-11 cells (Figure 5j). Relatively milder cytotoxic effects were observed for **1**, **12**, **15**, and **24** (Figure S7). Previous research suggested that ENL acts as a transcriptional activator, dysregulating gene expression and contributing to acute leukemia pathogenesis.^25^ Therefore, prolonging incubation with an ENL inhibitor is likely to yield stronger antileukemic effects. Extending the incubation period to 8 days revealed that exposure to inhibitor **13** led to increased cytotoxicity in a concentration-dependent manner, with *CC*_50_ values of 6.06 μM for MOLM-13 and 6.72 μM for MV4-11 (Figure 5a,b,j). In contrast, **1**, **12**, **15**, and **24** showed no significant increase in cytotoxicity. Furthermore, as shown in Figure 5c,j, the cytotoxicity effects of all tested inhibitors on Jurkat or HEK293T cells were nearly negligible, even at 10 μM of inhibitor **13** (Figure 5c and S7d), with marked cell death only occurring at 16 μM. Remarkably, **SR-C-107 (R)** exhibited outstanding antiproliferative effects in MOLM-13 and MV4-11 cells, with *CC*_50_ values of 1.25 and 0.81 μM, respectively (Figure 5d,e,j). Additionally, **YR-D-120** and **SR-C-107** exhibited comparable cellular inhibition activities, with *CC*_50_ values below 4 μM for both cell lines (Figure 5d,e,j). Unexpectedly, despite having comparable cellular ENL YEATS engagement and metabolic stability to **YR-D-120**, **YR-D-121** displayed weaker cytotoxicity, with *CC*_50_ values as 6.96 and 14.16 μM in two ENL-dependent leukemia cell lines (Figure 5d,e,j). Notably, none of the inhibitors showed antiproliferative activity in Jurkat or HEK293T cells, even at concentrations up to 64 μM (Figures 5c,f,j, and S7d).

We also monitored cell proliferation over approximately 2 weeks in the presence of an ENL YEATS inhibitor. Consistent with our earlier cytotoxicity assays, inhibitor **13** significantly impeded the growth of two ENL-dependent cell lines, MOLM-13 (85% inhibition, Figure S7f) and MV4-11 (75% inhibition, Figure S7g) at a concentration of 10 μM. However, at a lower concentration of 1 μM, the cellular inhibition efficacy was reduced, showing 30% and 41% inhibition for MOLM-13 and MV4-11, respectively (Figure S8a,b). In contrast, both **SR-C-107** and **SR-C-107 (R)** at 1 μM demonstrated strong inhibition of cell proliferation for MOLM-13 and MV4-11. Notably, **SR-C-107 (R)** achieved approximately 90% and 70% inhibition for MOLM-13 and MV4-11 cells, respectively (Figure 5g,h). Co-incubation with all inhibitors had no effect on the growth of Jurkat cells (Figures 5i, S7h and S8c). Further cell cycle analysis indicated an increase in the G1 phase for both MOLM-13 and MV4-11 cells after treatment with inhibitor **13** (Figure S8d) or **SR-C-107 (R)** (Figure 5k), supporting the notion that ENL depletion leads to G1 arrest during cell division.^26^ To confirm that the observed growth inhibition was due to the specific, on-target inhibition of ENL in cells, we conducted a cellular thermal shift assay (CETSA) to assess the thermal stability of ENL in both MOLM-13 and MV4-11 cells treated with **SR-C-107 (R)** or inhibitor **13**. These cells were treated with 10 μM of an inhibitor for 3 and 6 h. In both cell lines, we observed a higher abundance of ENL in samples treated with an inhibitor compared to those treated with DMSO at elevated temperatures (Figures 5l and S7i), indicating that **SR-C-107 (R)** or inhibitor **13** specifically engages with ENL within live cells. Additionally, we examined the expression levels of ENL target genes, including HOXA9, HOXA10, MEIS1, MYB and MYC, in MOLM-13 and MV4-11 cells following 72 h coincubation with **SR-C-107 (R)** or inhibitor **13**. We found that both inhibitors could effectively suppress the expression of these genes at concentrations as low as 1.0 μM (Figures 5m and S7j).

Among five previously reported ENL inhibitors (**1**, **12**, **13**, **15**, and **24**), our data strongly supported the notion that the growth inhibition observed with inhibitor **13** in ENL-dependent leukemia cells is specifically driven by its on-target activity. Moreover, the advanced compound, **SR-C-107 (R)**, which was developed from inhibitor **13**, demonstrated excellent antiproliferation effects in ENL-dependent leukemia cells that align well with the observed strong cell growth inhibition resulting from genetic disruption of ENL.^46^

### In Vivo PK Analysis and Antitumor Efficacy

**Figure 6:**
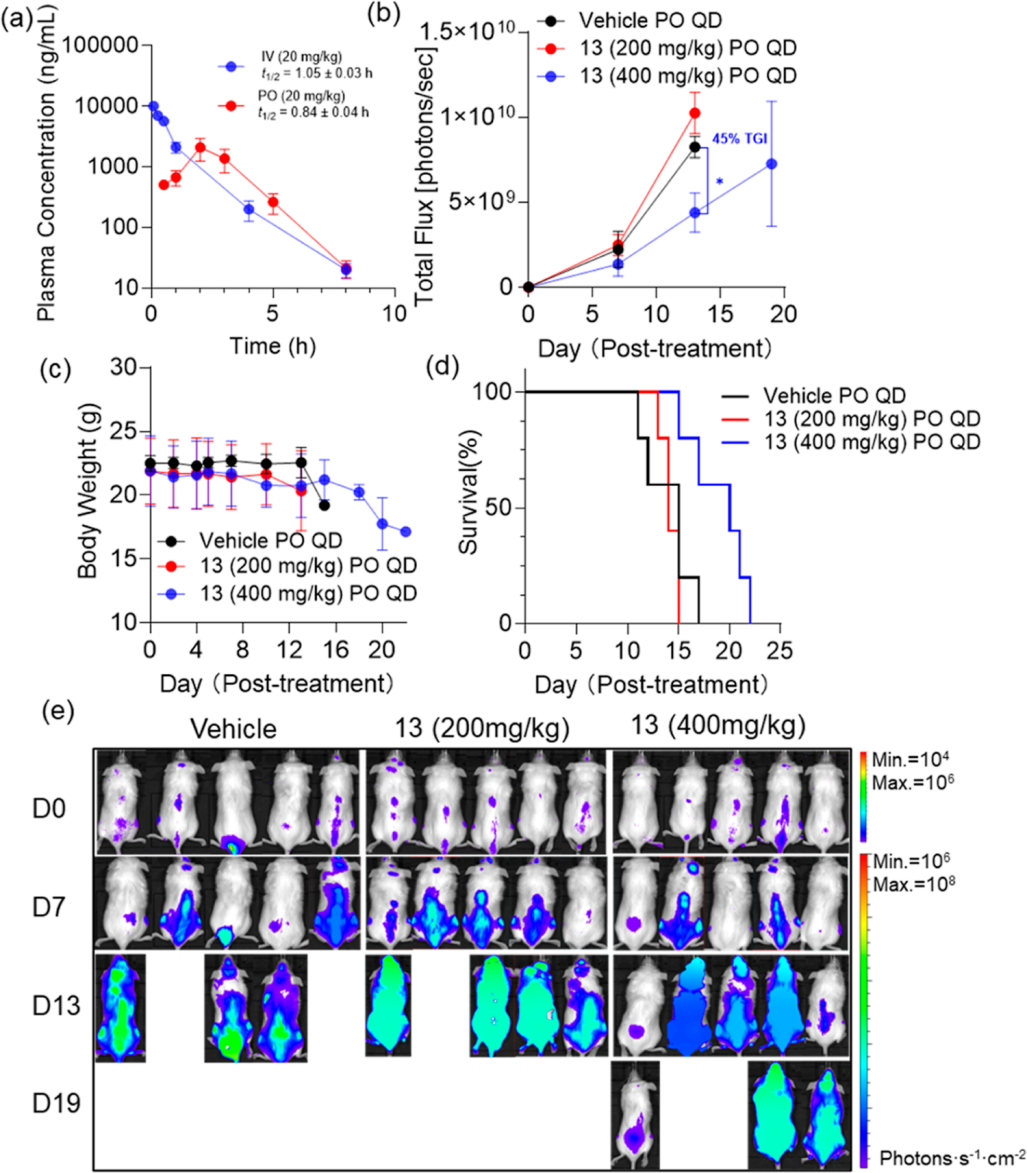
In vivo PK and antitumor analysis of inhibitor 13. (a) In vivo PK profile of inhibitor **13**. (b) Quantification of bioluminescence levels (mean ± SEM) in MOML-13 xenografted mice and (c) body weight of mice (mean ± SD) on the indicated days post treatment with inhibitor **13** or vehicle, tumors were allowed to grow for 7 days prior to treatment (day 0 marked the start of treatment). (d) Kaplan–Meier survival curves of NSG mice transplanted with MOLM-13 cells, treated with either vehicle or inhibitor 13 (*n* = 5). (e) Bioluminescent imaging of NSG mice.

**Figure 7:**
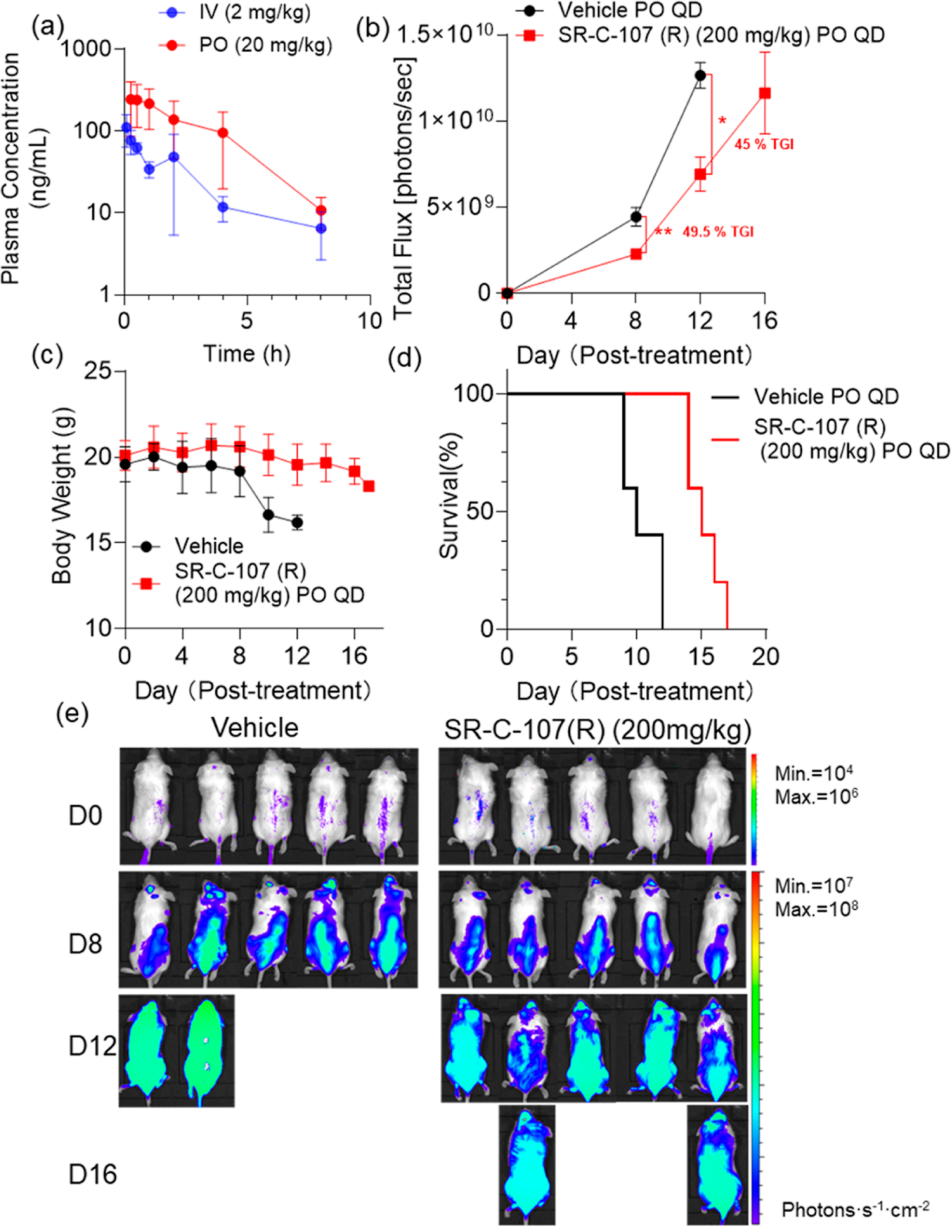
In vivo PK and antitumor analysis of **SR-C-107 (R)**. (**a**) In vivo PK profile of **SR-C-107 (R)**. (b) Quantification of bioluminescence levels (mean ± SEM) in MOML-13 xenografted mice and (c) body weight of mice (mean ± SD) on the indicated days post treatment with **SR-C-107 (R)** or vehicle, tumors were allowed to grow for 7 days prior to treatment (day 0 marked the start of treatment). (d) Kaplan–Meier survival curves of NSG mice transplanted with MOLM-13 cells, treated with either vehicle or inhibitor 13 (*n* = 5). (e) Bioluminescent imaging of NSG mice.

Given their extraordinary metabolic stability and promising cellular efficacy, both inhibitor **13** and **SR-C-107 (R)** wer advanced to in vivo PK and anititumor studies. After oral administration, inhibitor **13** showed excellent systemic exposure (*C*_max_ = 2080 ng/mL and *AUC*_0–inf_ = 5137 ng·h/mL) and a favorable half-life (*t*_1/2_ = 0.84 h) in mice (Figure 6a). A comparable half-life (*t*_1/2_ = 1.41 h) was observed for **SR-C-107 (R)** following oral administration at a dosage of 20 mg/kg (Figure 7a). However, its systemic exposure was lower, with an *AUC*_0–inf_ of 808 ng·h/mL. The oral *C*_max_ (285 ng/mL) of **SR-C-107 (R)** corresponds to an effective concentration of 0.75 μM, which was sufficient to induce antiproliferation effects based on its robust in vitro antiproliferation activity described earlier. These advantageous PK properties suggest that both inhibitors have the potential to achieve high and sustained drug concentrations through oral administration, providing a solid foundation for further animal efficacy studies on their antileukemia effects.

To induce the AML tumor phenotype, we intravenously injected 1 × 10^6^ MOLM-13-Luc cells, which stably expressed luciferase, allowing for bioluminescent imaging in mice. After 6 days of tumor development, the mice were divided into groups and administered daily gavage treatments of inhibitor **13** or **SR-C-107 (R)** (vehicle composed of 47.5% water, 5% DMSO, 45% PEG400, and 2.5% Tween 80). Based on PK results (Figures 6a and 7a) and *in vivo* dosages used for TDI-11055,^47^ we selected a 200 mg/kg oral daily dose (PO, QD) for the treatment. This dosage was well tolerated, with no significant body weight loss observed (Figures 6c and 7c). Tumor growth was monitored at various time points by measuring the luciferase-catalyzed bioluminescence in mice. While no noticeable tumor reduction was observed with 200 mg/kg of **13**, increasing the dose to 400 mg/kg resulted in significant reduction in leukemia burden after 13 days of treatment (corresponding to day 20 post-transplantation). This higher dose showed nearly 45% tumor growth inhibition (TGI) compared to the control group, without significant body weight loss until day 15 (Figure 6b,e). In the control group, all mice succumbed to the disease before the end of the treatment period (day 30 post-transplantation, Figure 6d) and displayed high levels of leukemia cells disseminated throughout their bodies (Figure 6a). In stark contrast, most mice in the 400 mg/kg inhibitor **13**-treated group not only survived but also displayed a marked reduction in AML burden. The overall survival in the 400 mg/kg inhibitor **13**-treated group was significantly improved compared to the control group (Figure 6d, *P* = 0.00185), with a 33.3% increase in median survival (from 15 to 20 post-treatment days). Notably, the in vivo antitumor efficacy of **SR-C-107 (R)** at 200 mg/kg surpassed that of inhibitor **13** at 400 mg/kg. **SR-C-107 (R)** demonstrated significant TGI, achieving 49.5% TGI after 8 days of treatment (*P* = 0.0058) and 45% TGI (*P* = 0.0206) at day 12 (Figure 7b,c,e). Additionally, the median survival time was extended from 10 to 15 days post-treatment. **SR-C-107 (R)** at 200 mg/kg was well tolerated, with no significant body weight loss observed (Figure 7d). Collectively, these findings suggest that **SR-C-107 (R)** has a favorable safety profile and that structural optimization has effectively reduced the efficacious dose compared to inhibitor **13**.

### Chemistry

The synthetic route to prepare key intermediates (**7a**, **7b**) is illustrated in Scheme 1. Compound **2** was treated with NaBH_4_, followed by OBHA·HCl and Al(CH_3_)_3_, to yield compound **4**. **4** was treated with TPP and TEA in CCl_4_ to produce a racemic mixture of compound **5**. Racemic **5** was separated using a CHIRALCEL OD Preparative column to obtain optically pure compounds **5a** and **5b**, which were subsequently treated with Raney Ni, followed by LAH, TMS-Cl, and 2 M HCl, to afford key intermediates **7a** and **7b**. The convergent synthesis of target compounds (Scheme 2) involved primarily reductive amination, SEM protection, Buchwald–Hartwig cross-coupling, and acid-amine coupling. Final deprotection of the SEM group in DCM with TFA yielded corresponding target compounds **YR-D-120**, **YR-D-121**, **SR-C-107**, **SR-C-107 (R)** and **SR-C-107 (S)**.

**Scheme 3:**
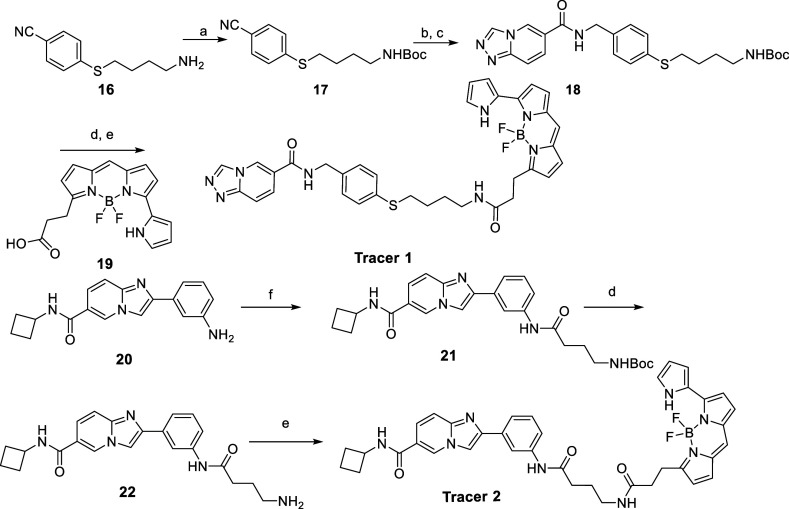
Synthesis of Fluorescence Tracers Reagents and conditions: (a) (Boc)_2_O, Et_3_N, ACN, 16 h; (b) LiAlH_4_, dry THF, 0 °C-rt, 4 h; (c) [1,2,4]triazolo[4,3-a]pyridine-6-carboxylic acid, EDC, DIPEA, DMF, rt, 16 h; (d) 4 M HCl, 1,4-dioxane, 2 h; (e) **19**, DMAP, EDC, DMF, 36 h; (f) 4-((*tert*-butoxycarbonyl)amino)butanoic acid, HATU, DIPEA, DMF, rt, 16 h.

**Tracer 1** was synthesized from compound **16** (Scheme 3), which was obtained through Boc protection, followed by LAH reduction and acid-amine coupling to afford compound **18**. Removal of the *N*-Boc group, followed by a second acid-amine coupling reaction, produced **Tracer 1**. Similarly, **Tracer 2** was synthesized from compound **20** (Scheme 3), which was also obtained via acid-amine coupling. After deprotecting the *N*-Boc group, an additional acid-amine coupling reaction was performed to generate **Tracer 2**.

## Conclusions

In summary, we have successfully engineered an ENL-NanoBRET assay system to assess cellular potency of ENL inhibitors. Utilizing this novel platform, we prioritized five previously developed ENL YEATS inhibitors, **1**, **12**, **13**, **15**, and **24** for in-depth characterizations. Metabolic stability evaluations conducted in human plasma and liver microsomes highlighted inhibitor **13** for its slow elimination rate and enhanced bioavailability compared to other inhibitors. Inhibitor **13** demonstrated strong antiproliferation activity toward ENL-dependent leukemia cells and displayed robust anti-AML effects in xenografted mice. Based on the structure of inhibitor **13**, we developed a series of new compounds. Among them, **SR-C-107 (R)**, compared to inhibitor **13** exhibited superior inhibitory activity against ENL-dependent leukemia both in vitro and xenografted mice. We believe the current research has laid a strong foundation for advancing **SR-C-107 (R)** to further preclinical studies and provides a streamlined strategy for the development of next-generation AML inhibitors.

## Experimental Section

### Chemistry

All reagents and solvents for synthesis were purchased from commercial sources and used without purification. All glassware was flame-dried prior to use. Thin layer chromatography (TLC) was carried out on aluminum plates coated with 60 F254 silica gel. TLC plates were visualized under UV light (254 or 365 nm) or stained with 5% phosphomolybdic acid. Normal phase column chromatography was carried out using a Yamazen Smart Flash AKROS system. Analytical reverse-phase high-pressure liquid chromatography was carried out on a Shimadzu LC20 HPLC system with an analytical C_18_ column. The mobile phases were H_2_O with 0.1% formic acid (A) and acetonitrile with 0.1% formic acid (B) if not mentioned otherwise. Isomer separation was conducted through Preparative HPLC on an Agilent 1260 Infinity II Preparative LC System with a CHIRALCEL OD Preparative column, 50 mm ID, 500 mm length, 20 μm particle size. The mobile phases were isopropyl alcohol (A) and hexane not mentioned otherwise. All isomers were analyzed by HPLC (CHIRALCEL OD-H (4.6 × 250 mm), 5 μ, hexane/2-propanol 50:50, 0.1% DEA, 1 mL/min). NMR spectra were recorded on a Bruker AVANCE Neo 400 MHz or Varian INOVA 300 MHz spectrometer in specified deuterated solvents. High-resolution electrospray ionization mass spectrometry (HRMS-ESI) was carried out on a Themo Scientific QExactive Focus system. All compounds are >95% pure by analytical reverse-phase HPLC.

#### Methyl 3-Hydroxy-4-methylpentanoate (**3**)

The keto ester **2** (25 g, 172.7 mmol) was dissolved in absolute MeOH (100 mL) and cooled to −10 °C under inert atmosphere. To the resulting solution was added sodium borohydride (2.62 g, 69 mmol) portion wise over several minutes and the starting material was consumed (TLC analysis). The reaction was then quenched with acetone and after several minutes concentrated on the rotary evaporator. The residue was dissolved in EtOAc and washed with 1.2 M HCl followed by saturated NaHCO_3_ and brine. The organic phase was dried over sodium sulfate, filtered, concentrated on the rotary evaporator, and the residue was purified by filtration through a pad of silica or flash chromatography to afford **3** as an oily liquid (22.7 g, 90%). ^1^H NMR (400 MHz, CDCl_3_): δ 3.78 (ddd, *J* = 9.6, 5.7, 2.9 Hz, 1H), 2.56–2.35 (m, 2H), 1.70 (pd, *J* = 6.9, 5.9 Hz, 1H), 0.93 (dd, *J* = 11.2, 6.8 Hz, 6H).

#### *N*-(Benzyloxy)-3-hydroxy-4-methylpentanamide (**4**)

To an ice bath cooled suspension of OBHA·HCl (2 equiv) in dry CH_2_Cl_2_ (88 mL) was slowly added AlMe3 (2.0 M in heptane, 2 equiv) via syringe. After the evolution of gas subsided, the resulting solution was then warmed to room temperature and stirred for 1 h. The resulting clear solution was then cooled to 0 °C and a solution of the β-hydroxy ester **3** (22 g 150 mmol) in CH_2_Cl_2_ (88 mL) was added via cannula. The heterogeneous mixture was stirred for 12–15 h at room temperature and then quenched by the addition of 1.2 M HCl. The two layers were separated, and the aqueous layer was extracted (4 × 50 mL) with CH_2_Cl_2_. The extract and the original organic layer were combined and washed with 1.2 M HCl (100 mL), saturated NaHCO_3_ (100 mL), and brine (100 mL). The organic phase was then dried over Na_2_SO_4_, filtered, and concentrated on a rotary evaporator to provide a crude product. The crude product was purified by recrystallization by using dichloromethane and hexane to get pure compound **4** as a white solid (28.8 g, 81%). ^1^H NMR (400 MHz, DMSO-*d*_6_): δ 10.93 (s, 1H), 7.45–7.29 (m, 5H), 4.78 (s, 2H), 4.59 (d, *J* = 5.2 Hz, 1H), 3.65 (dq, *J* = 9.2, 4.7 Hz, 1H), 2.12–1.89 (m, 2H), 1.54 (pd, *J* = 6.8, 4.7 Hz, 1H), 0.82 (dd, *J* = 8.1, 6.8 Hz, 6H). ^13^C NMR (101 MHz, DMSO-*d*_6_): δ 168.37, 136.14, 128.74, 128.27, 128.17, 76.78, 71.52, 37.72, 33.06, 18.77, 17.13.

#### 1-(Benzyloxy)-4-isopropylazetidin-2-one (**5**)

A round-bottom flask was charged with **4** (28.4 g, 120 mmol) and triphenylphosphine (37.77 g, 144 mmol). The flask was then purged with argon and freshly distilled acetonitrile (170 mL) was added by syringe. The resulting slurry was cooled with an ice bath to 0 °C. To this cooled solution was added freshly distilled triethylamine (41.8 mL, 300 mmol) followed by dropwise addition of carbon tetrachloride (13.96 mL, 144 mmol). After 30 min, the ice bath was removed, and the reaction was warmed to room temperature and stirred for 20–26 h. The reaction mixture was then concentrated on a rotary evaporator and the residue was redissolved in EtOAc or diethyl ether and filtered through a pad of silica by vacuum filtration to remove the hydrochloride salts. The filtrate was then concentrated and chromatographed to provide the desired β-lactam **5** as a white solid (21.8 g, 83%). ^1^H NMR (400 MHz, DMSO-*d*_6_): δ 7.47–7.34 (m, 5H), 4.99–4.85 (m, 2H), 3.60 (ddd, *J* = 6.2, 5.3, 2.5 Hz, 1H), 2.61 (dd, *J* = 13.6, 5.4 Hz, 1H), 2.37 (dd, *J* = 13.7, 2.5 Hz, 1H), 1.81 (dq, *J* = 13.4, 6.7 Hz, 1H), 0.92 (d, *J* = 6.8 Hz, 3H), 0.82 (d, *J* = 6.8 Hz, 3H). ^13^C NMR (101 MHz, DMSO-*d*_6_): δ 163.58, 135.27, 129.04, 128.60, 128.39, 76.58, 62.02, 34.32, 29.78, 18.43, 17.40.

#### Chiral Separation of **5**

Compound **5** was separated using a chiral column to get two enantiomers which were isomer-1 (**5a**) and isomer-2 (**5b**). The configuration of isomer-1 (**5a**) and isomer-2 (**5b**) were confirmed by small molecule X-ray crystallography analysis. Briefly, single colorless plate-shaped crystals of compound were used. A suitable crystal with dimensions 0.12 × 0.07 × 0.03 mm^3^ was selected and mounted on a MITIGEN holder on a XtaLAB Synergy X-ray diffractometer. The crystal was kept at a steady *T* = 100.00(10) K during data collection. The structure was solved with the ShelXT solution program using dual methods and by using Olex2 1.5 as the graphical interface. The model was refined with ShelXL 2019/1 using full matrix least-squares minimization on *F*^2^. More data details can be found in the X-ray identification report.

#### (*S*)-4-Isopropylazetidin-2-one (**6a**)

To a solution of compound **5a** (10.0 g, 45.63 mmol) in methanol (150 mL) was added freshly prepared W2 Raney-nickel (suspension in methanol, 60 g), and the mixture was stirred for 16 h under hydrogen atmosphere. The reaction mixture was filtered through a pad of Celite and washed with methanol (2 × 30 mL). The combined organic layers were concentrated under reduced pressure, and the residue was purified by column chromatography using 5–10% methanol/dichloromethane (v/v) as the eluent to afford pure product **6a** as a white solid (4.9 g, 95%). ^1^H NMR (400 MHz, CDCl_3_): δ 6.40 (s, 1H), 3.30 (ddd, *J* = 7.8, 5.0, 2.4 Hz, 1H), 3.02–2.90 (m, 1H), 2.58 (ddd, 1H), 1.78–1.60 (m, 1H), 0.91 (dd, *J* = 17.1, 6.7 Hz, 6H). ^13^C NMR (101 MHz, CDCl_3_): δ 168.74, 54.03, 41.23, 32.69, 18.53, 17.80.

#### (*R*)-4-Isopropylazetidin-2-one (**6b**) was Synthesized through the Same Method as **6a**

^1^H NMR (400 MHz, CDCl_3_): δ 6.12 (s, 1H), 3.31 (ddd, *J* = 7.7, 5.0, 2.4 Hz, 1H), 3.02–2.92 (m, 1H), 2.60 (ddd, *J* = 14.9, 2.5, 1.2 Hz, 1H), 1.77–1.64 (m, 1H), 0.93 (dd, *J* = 15.9, 6.7 Hz, 6H). ^13^C NMR (101 MHz, CDCl_3_): δ 168.57, 54.14, 41.40, 32.78, 18.65, 17.90.

#### (*S*)-2-Isopropylazetidine Hydrochloride (**7a**)

2 M LiAlH_4_ solution in THF (65 mL, 130 mmol) was suspended in dry THF (130 mL) and the resulting solution was cooled to 0 °C. Trimethylsilyl chloride (17.0 mL, 134.4 mmol) was added dropwise, and the solution was stirred for 2 h at the same temperature. The mixture was cooled to −20 °C and the compound **6a** (4.9 g, 43.36 mmol) was added in small portions. The resulting mixture was stirred for 2 days at room temperature, and then the excess of LiAlH_4_ was quenched with 40% aq. NaOH solution. Inorganic precipitates were filtered out. The filtrate was cooled to 0 °C and 2 M hydrochloric acid (43.3 mL, 86.7 mmol) was added to the filtrate. The mixture was stirred for 5–10 min at the same temperature. The tetrahydrofuran was evaporated from the reaction mass and the aqueous layer was evaporated under lyophilization to get pure **7a** as white solid (5.41 g, 39.88 mmol). ^1^H NMR (400 MHz, DMSO-*d*_6_): δ 9.39 (s, 2H), 3.99–3.87 (m, 1H), 3.87–3.74 (m, 1H), 3.65–3.53 (m, 1H), 2.37–2.26 (m, 1H), 2.26–2.16 (m, 1H), 2.16–2.04 (m, 1H), 0.88 (d, *J* = 6.5 Hz, 3H), 0.80 (d, *J* = 6.8 Hz, 3H). ^13^C NMR (101 MHz, DMSO-*d*_6_): δ 65.30, 40.49, 31.14, 23.36, 17.78, 16.42.

#### (*R*)-2-Isopropylazetidine Hydrochloride (**7b**) was Synthesized through the Same Method as **7a**

^1^H NMR (400 MHz, DMSO-*d*_6_): δ 9.41 (s, 2H), 3.99–3.87 (m, 1H), 3.86–3.73 (m, 1H), 3.66–3.53 (m, 1H), 2.36–2.26 (m, 1H), 2.26–2.16 (m, 1H), 2.16–2.06 (m, 1H), 0.87 (d, *J* = 6.5 Hz, 3H), 0.80 (d, *J* = 6.8 Hz, 3H). ^13^C NMR (101 MHz, DMSO-*d*_6_): δ 65.41, 40.61, 31.21, 23.41, 17.82, 16.47.

#### (*S*)-6-Chloro-2-((2-methylpyrrolidin-1-yl)methyl)-1*H*-pyrrolo[3,2-c]pyridine (**8a**)

To a stirred solution of 6-chloro-1*H*-pyrrolo[3,2-c]pyridine-2-carbaldehyde (2.0 g, 11.1 mmol) and (*S*)-2-methylpyrrolidine (2.25 g, 16.6 mmol) in anhydrous DCE (40 mL) at 0 °C was added acetic acid (0.66 g, 11.1 mmol). The mixture was stirred under room temperature for 3 h. Then NaBH(OAc)_3_ was added and the mixture was stirred at same temperature for 12 h. After completion of reaction, the reaction was quenched with saturated NaHCO_3_ solution (50 mL) and extracted with EtOAc (2 × 50 mL). The combined organic layer was washed with brine, dried over MgSO_4_, and concentrated in a vacuum. The residue was then purified with flash chromatography (0–10% MeOH in DCM as the eluent) to afford **8a** as a yellow solid (1.5 g, 60%). ^1^H NMR (400 MHz, DMSO-*d*_6_): δ 11.67 (s, 1H), 8.52 (d, *J* = 0.8 Hz, 1H), 7.33 (t, *J* = 0.9 Hz, 1H), 6.47 (s, 1H), 4.11–4.05 (m, 1H), 3.48 (d, *J* = 14.0 Hz, 1H), 2.88 (ddd, *J* = 9.3, 7.1, 4.0 Hz, 1H), 2.24 (q, *J* = 8.8 Hz, 1H), 1.99–1.92 (m, 1H), 1.65 (tdd, *J* = 8.4, 6.4, 3.4 Hz, 2H), 1.45–1.31 (m, 1H), 1.11 (d, *J* = 6.1 Hz, 3H).

#### 6-Chloro-2-((2-cyclopropylazetidin-1-yl)methyl)-1*H*-pyrrolo[3,2-c]pyridine (**8b**) was Synthesized through the Same Method as **8a**

^1^H NMR (400 MHz, DMSO-*d*_6_): δ 11.50 (s, 1H), 8.44 (d, *J* = 0.9 Hz, 1H), 7.24 (t, *J* = 0.9 Hz, 1H), 6.35 (s, 1H), 3.97 (q, *J* = 7.1 Hz, 1H), 3.75 (d, *J* = 14.0 Hz, 1H), 3.49 (d, *J* = 13.9 Hz, 1H), 3.08 (ddd, *J* = 8.6, 6.6, 2.3 Hz, 1H), 2.68 (ddd, *J* = 9.3, 7.9, 6.5 Hz, 1H), 2.58 (q, *J* = 7.8 Hz, 1H), 1.99–1.87 (m, 1H), 1.82–1.68 (m, 1H), 0.81 (qt, *J* = 8.0, 4.9 Hz, 1H), 0.31–0.13 (m, 2H), 0.06 to −0.06 (m, 2H). HRMS (ESI+) was calcd for C_21_H_22_N_7_O [M + H]^+^, 388.1880; found, 388.1874.

#### 6-Chloro-2-((2-isopropylazetidin-1-yl)methyl)-1*H*-pyrrolo[3,2-c]pyridine (**8c**) was Synthesized through the Same Method as **8a**

^1^H NMR (400 MHz, DMSO-*d*_6_): δ 11.53 (s, 1H), 8.51 (d, *J* = 9.1 Hz, 1H), 7.32 (d, *J* = 3.4 Hz, 1H), 6.42 (s, 1H), 3.94–3.45 (m, 2H), 3.16 (t, *J* = 5.6 Hz, 1H), 3.02–2.66 (m, 2H), 1.95 (dd, *J* = 20.1, 11.6 Hz, 1H), 1.81–1.69 (m, 1H), 1.60 (dd, *J* = 13.3, 6.5 Hz, 1H), 0.81 (dd, *J* = 12.3, 6.7 Hz, 6H).

#### (*S*)-6-Chloro-2-((2-methylpyrrolidin-1-yl)methyl)-1-((2-(trimethylsilyl)ethoxy)methyl)-1*H*-pyrrolo[3,2-c]pyridine (**9a**)

To a stirred solution of **8a** (350 mg, 1.40 mmol) in anhydrous THF (10 mL) at 0 °C was added NaH (4.21 mmol, 0.101 g, 60%). Then SEM-Cl (234 mg, 1.40 mmol) was added to the reaction dropwise at 0 °C. The temperature was raised slowly to room temperature and the reaction mixture was stirred for 2 h. The mixture was then poured into water (10 mL) and extracted with EtOAc (2 × 20 mL). The organic layer was dried over anhydrous Na_2_SO_4_ and then concentrated in vacuo. The residue was then purified with flash chromatography (0–50% EtOAc in hexane as the eluent) to afford **9a** as a yellow gummy solid (300 mg, 56%). ^1^H NMR (400 MHz, CDCl_3_): δ 8.56 (d, *J* = 0.9 Hz, 1H), 7.37 (t, *J* = 0.9 Hz, 1H), 6.44 (s, 1H), 5.78 (d, *J* = 10.9 Hz, 1H), 5.49 (d, *J* = 10.8 Hz, 1H), 4.16 (dd, *J* = 13.4, 1.0 Hz, 1H), 3.49 (dtd, *J* = 24.1, 9.1, 7.3 Hz, 2H), 3.32 (d, *J* = 13.5 Hz, 1H), 2.86–2.75 (m, 1H), 2.42 (ddt, *J* = 13.8, 8.0, 6.1 Hz, 1H), 2.15 (q, *J* = 8.9 Hz, 1H), 2.07–1.91 (m, 1H), 1.66 (tt, *J* = 8.8, 5.8 Hz, 2H), 1.50–1.35 (m, 1H), 1.16 (d, *J* = 6.0 Hz, 3H), 0.99–0.81 (m, 3H), −0.05 (s, 9H).

#### 6-Chloro-2-((2-cyclopropylazetidin-1-yl)methyl)-1-((2(trimethylsilyl)ethoxy)methyl)-1-pyrrolo[3,2-c]pyridine (**9b**) was Synthesized through the Same Method as **9a**

^1^H NMR (400 MHz, CDCl3): δ 8.65–8.57 (m, 1H), 7.45–7.35 (m, 1H), 6.47 (s, 1H), 5.71 (q, *J* = 11.0 Hz, 2H), 4.01 (t, *J* = 13.8 Hz, 1H), 3.63–3.47 (m, 3H), 3.22 (s, 1H), 2.84–2.77 (m, 1H), 2.67 (d, *J* = 9.7 Hz, 1H), 2.19–2.00 (m, 1H), 2.00–1.91 (m, 1H), 1.64 (s, 2H), 1.05–0.86 (m, 1H), 0.45–0.38 (m, 2H), 0.12 (tq, *J* = 13.3, 4.9 Hz, 2H), −0.00 (s, 9H).

#### 6-Chloro-2-((2-isopropylazetidin-1-yl)methyl)-1-((2(trimethylsilyl)ethoxy)methyl)-1*H*-pyrrolo[3,2-c]pyridine (**9c**) was Synthesized through the Same Method as **9a**

^1^H NMR (400 MHz, CDCl3): δ 8.61 (d, *J* = 0.6 Hz, 1H), 7.41 (s, 1H), 6.49 (s, 1H), 5.81 (d, *J* = 11.0 Hz, 1H), 5.58 (d, *J* = 11.0 Hz, 1H), 4.08 (d, *J* = 13.3 Hz, 1H), 3.60–3.48 (m, 3H), 3.22–3.04 (m, 1H), 3.01–2.87 (m, *J* = 15.8, 7.6 Hz, 1H), 2.88–2.68 (m, *J* = 16.5, 8.4 Hz, 1H), 2.10–1.95 (m, 1H), 1.96–1.79 (m, *J* = 17.5, 8.7 Hz, 1H), 1.79–1.66 (m, 1H), 0.96–0.88 (m, 8H), 0.04 to −0.06 (m, 9H).

#### (*S*)-*N*-(2-((2-Methylpyrrolidin-1-yl)methyl)-1-((2-(trimethylsilyl)ethoxy)methyl)-1*H*-pyrrolo[3,2-c]pyridin-6-yl)-1,1-diphenylmethanimine (**10a**)

A stirred solution of **9a** (300 mg, 0.787 mmol), benzophenone imine (213 mg, 1.18 mmol), sodium *tert*-butoxide (150 mg, 1.57 mmol), tris(dibenzylideneacetone)dipalladium(0) (0.017 g, 0.064 mmol) and 2,2′-bis(diphenylphosphino)-1,1′-binaphthyl (0.02 g, 0.064 mmol) in Toluene (5 mL) was degassed and purged with N_2_ for 3 times, and then the mixture was stirred at 110 °C for 4 h. The reaction mixture was filtered and concentrated in vacuo and used in the next step without further purification.

1*H*-Pyrrolo[3,2-c] pyridin-6-yl)-1,1-diphenylmethanimine (**10b**) and *N*-(2-((2-isopropylazetidin-1-yl) methyl)-1-((2-(trimethylsilyl) ethoxy) methyl)-1*H*-pyrrolo[3,2-c] pyridin-6-yl)-1,1-diphenylmethanimine (**10c**) were synthesized through the same method as **10a**.

#### (*S*)-2-((2-Methylpyrrolidin-1-yl)methyl)-1-((2-(trimethylsilyl)ethoxy)methyl)-1*H*-pyrrolo[3,2-c]pyridin-6-amine (**11a**)

To a stirred solution of crude **10a** (300 mg) in THF/H_2_O (1:1, 10 mL) was added 1 M HCl (5 mL) and the reaction solution was stirred at room temperature for 16 h. The mixture was then poured into water (10 mL) and extracted with EtOAc. The aqueous phase was basified using 1 N NaOH to adjust pH to 8. The aqueous layer was extracted with EtOAc (2 × 30 mL). The organic layer was dried over anhydrous Na_2_SO_4_ and then concentrated in vacuo and used in the next step without further purification. ^1^H NMR (400 MHz, DMSO-*d*_6_): δ 8.51 (d, *J* = 0.9 Hz, 1H), 7.88 (t, *J* = 0.9 Hz, 1H), 6.50 (s, 1H), 5.76 (d, *J* = 11.1 Hz, 1H), 5.58 (d, *J* = 11.0 Hz, 1H), 4.21 (d, *J* = 13.5 Hz, 1H), 3.71–3.53 (m, 2H), 3.38 (d, *J* = 13.5 Hz, 1H), 2.89 (ddd, *J* = 9.7, 6.4, 4.0 Hz, 1H), 2.50 (td, *J* = 7.6, 5.8 Hz, 1H), 2.25 (q, *J* = 8.8 Hz, 1H), 2.12–1.99 (m, 1H), 1.71 (q, *J* = 8.3 Hz, 2H), 1.52–1.38 (m, 1H), 1.22 (d, *J* = 5.9 Hz, 3H), 1.04–0.86 (m, 2H), 0.00 (s, 9H).

#### 2-((2-Cyclopropylazetidin-1-yl)methyl)-1-((2-(trimethylsilyl)ethoxy)methyl)-1*H*-pyrrolo[3,2-c]pyridin-6-amine (**11b**) was Synthesized through the Same Method as **11a**

^1^H NMR (400 MHz, DMSO-*d*_6_): δ 8.50 (s, 1H), 7.87 (s, 1H), 6.48 (s, 1H), 5.75–5.61 (m, 2H), 3.99 (d, *J* = 13.5 Hz, 1H), 3.70–3.54 (m, 3H), 3.17 (d, *J* = 7.8 Hz, 1H), 2.80 (dt, *J* = 15.5, 8.2 Hz, 2H), 2.10 (d, *J* = 9.9 Hz, 1H), 1.89 (p, *J* = 9.0 Hz, 1H), 0.96 (t, *J* = 7.9 Hz, 3H), 0.42 (td, *J* = 6.4, 2.4 Hz, 2H), 0.26–0.15 (m, 2H), 0.00 (s, 9H).

#### 2-((2-Isopropylazetidin-1-yl)methyl)-1-((2-(trimethylsilyl)ethoxy)methyl)-1*H*-pyrrolo[3,2-c]pyridin-6-amine (**11c**) was Synthesized through the Same Method as **11a**

^1^H NMR (400 MHz, DMSO-*d*_6_): δ 8.20 (d, *J* = 0.7 Hz, 1H), 6.51 (s, 1H), 6.33 (s, 1H), 5.64 (d, *J* = 11.1 Hz, 1H), 5.47 (d, *J* = 11.1 Hz, 1H), 5.43 (s, 2H), 4.29–4.10 (m, *J* = 28.9, 5.9 Hz, 1H), 3.97 (d, *J* = 13.2 Hz, 1H), 3.60–3.51 (m, 2H), 3.15–3.01 (m, 1H), 3.00–2.88 (m, *J* = 15.5, 7.5 Hz, 1H), 2.86–2.72 (m, *J* = 16.2, 8.5 Hz, 1H), 2.08–1.93 (m, 1H), 1.86–1.73 (m, 1H), 1.73–1.62 (m, *J* = 13.6, 6.8 Hz, 1H), 0.99–0.82 (m, 8H), 0.04 to −0.03 (m, 9H).

#### (*S*)-*N*-(2-((2-Methylpyrrolidin-1-yl)methyl)-1-((2-(trimethylsilyl)ethoxy)methyl)-1*H*-pyrrolo[3,2-c]pyridin-6-yl)-[1,2,4]triazolo[4,3-a]pyridine-6-carboxamide (**12a**)

To a stirred solution of crude **11a** (300 mg) and [1,2,4]triazolo[4,3-a]pyridine-6-carboxylic acid (174 mg, 0.916 mmol) in anhydrous pyridine (5 mL) at 0 °C was added EDCI (280 mg, 1.25 mmol). Reaction mixture was stirred at 50 °C for 12 h. After completion of reaction, the reaction mixture was added with H_2_O (10 mL), extracted with EtOAc (2 × 20 mL), and washed with saturated brine solution (2 × 10 mL) sequentially. The organic layer was dried over anhydrous Na_2_SO_4_ and then concentrated in vacuo. The residue was then purified with flash chromatography (0–10% MeOH in CH_2_Cl_2_ as the eluent) to afford **12a** as a white solid (170 mg, 41%). ^1^H NMR (400 MHz, DMSO-*d*_6_): δ 11.00 (s, 1H), 9.49 (d, *J* = 0.8 Hz, 1H), 9.43 (t, *J* = 1.4 Hz, 1H), 8.67 (d, *J* = 1.0 Hz, 1H), 8.46 (s, 1H), 8.03–7.90 (m, 2H), 6.63 (s, 1H), 5.82 (d, *J* = 11.1 Hz, 1H), 5.69 (d, *J* = 11.1 Hz, 1H), 4.25 (d, *J* = 13.6 Hz, 1H), 3.61 (dtd, *J* = 30.0, 9.2, 6.8 Hz, 2H), 2.92–2.82 (m, 1H), 2.50 (p, *J* = 6.7 Hz, 1H), 2.26 (q, *J* = 8.8 Hz, 1H), 2.04 (dt, *J* = 14.6, 7.2 Hz, 1H), 1.71 (q, *J* = 7.8 Hz, 2H), 1.45 (dq, *J* = 12.2, 8.3 Hz, 1H), 1.22 (d, *J* = 5.9 Hz, 3H), 1.04–0.86 (m, 2H), 0.00 (s, 9H).

#### *N*-(2-((2-Cyclopropylazetidin-1-yl)methyl)-1-((2-(trimethylsilyl)ethoxy)methyl)-1*H*-pyrrolo[3,2-c]pyridin-6-yl)-[1,2,4]triazolo[4,3-a]pyridine-6-carboxamide (**12b**) was Synthesized through the Same Method as **12a**

^1^H NMR (400 MHz, DMSO-*d*_6_): δ 10.99 (s, 1H), 9.48 (d, *J* = 0.8 Hz, 1H), 9.42 (t, *J* = 1.4 Hz, 1H), 8.66 (d, *J* = 0.9 Hz, 1H), 8.44 (s, 1H), 8.00–7.89 (m, 2H), 6.60 (s, 1H), 5.83–5.71 (m, 2H), 4.03 (d, *J* = 13.7 Hz, 1H), 3.76–3.50 (m, 3H), 3.21–3.13 (m, 1H), 2.81 (dq, *J* = 20.0, 7.9 Hz, 2H), 2.15–2.04 (m, 1H), 1.89 (p, *J* = 9.0 Hz, 1H), 1.02–0.88 (m, 3H), 0.41 (ddd, *J* = 9.2, 4.6, 2.9 Hz, 2H), 0.25–0.14 (m, 2H), 0.00 (s, 9H).

#### *N*-(2-((2-Isopropylazetidin-1-yl)methyl)-1-((2-(trimethylsilyl)ethoxy)methyl)-1*H*-pyrrolo[3,2-c]pyridin-6-yl)-[1,2,4]triazolo[4,3-a]pyridine-6-carboxamide (**12c**) was Synthesized through the Same Method as **12a**

^1^H NMR (400 MHz, DMSO-*d*_6_): δ 11.01 (s, 1H), 9.50 (s, 1H), 9.43 (s, 1H), 8.68 (s, 1H), 8.46 (s, 1H), 8.05–7.86 (m, 2H), 6.63 (s, 1H), 5.84 (d, *J* = 11.3 Hz, 1H), 5.71 (d, *J* = 11.2 Hz, 1H), 4.11 (d, *J* = 13.5 Hz, 1H), 3.73–3.55 (m, 3H), 3.14 (s, 1H), 3.02 (s, 1H), 2.82 (d, *J* = 36.5 Hz, 1H), 2.06 (d, *J* = 7.7 Hz, 1H), 1.92–1.65 (m, *J* = 19.9, 15.7, 7.8 Hz, 2H), 0.99–0.88 (m, 8H), −0.00 (s, 9H).

#### (*S*)-*N*-(2-((2-Methylpyrrolidin-1-yl)methyl)-1*H*-pyrrolo[3,2-c]pyridin-6-yl)-[1,2,4]triazolo [4,3-a]pyridine-6-carboxamide (**YR-D-120**)

To a stirred solution of **12a** (120 mg, 0.237 mmol) in anhydrous DCM (5 mL) at 0 °C was added TFA (1 mL). Reaction mixture was stirred at room temperature for 12 h. After completion of reaction, the solvent was removed in vacuo. The residue was then purified with reverse phase flash chromatography (0–100% ACN in H_2_O as the eluent) to afford **YR-D-120** as a white solid (30 mg, 34%). ^1^H NMR (400 MHz, CD_3_OD): δ 9.39 (s, 1H), 9.34 (s, 1H), 8.93 (s, 1H), 8.09–8.01 (m, 2H), 7.94 (d, *J* = 9.6 Hz, 1H), 7.16 (s, 1H), 4.54 (d, *J* = 14.2 Hz, 1H), 3.77–3.56 (m, 2H), 3.47–3.38 (m, 2H), 2.48–2.39 (m, 1H), 2.23–2.01 (m, 2H), 1.90–1.76 (m, 1H), 1.54 (d, *J* = 6.6 Hz, 3H). ^13^C NMR (126 MHz, CD_3_OD): δ 164.60, 161.95, 161.68, 144.55, 141.57, 135.45, 134.44, 127.96, 127.36, 122.60, 121.45, 114.55, 106.81, 97.91, 89.73, 64.61, 53.90, 31.04, 20.92, 14.98. HRMS (ESI+) was calcd for C_22_H_26_N_55_O [M + H]^+^, 376.1880; found, 376.1874.

#### *N*-(2-(2-Cyclopropylazetidin-1-yl)methyl)-1*H*-pyrrolo[3,2-c]pyridin-6-yl)-[1,2,4] triazolo[4,3-a]pyridine-6-carboxamide (**YR-D-121**) was Synthesized through the Same Method as **YR-D-120**

^1^H NMR (400 MHz, CD_3_OD): δ 9.12 (d, *J* = 0.9 Hz, 1H), 9.01 (t, *J* = 1.4 Hz, 1H), 8.44 (d, *J* = 1.0 Hz, 1H), 8.30 (s, 1H), 8.05 (s, 1H), 7.77 (dd, *J* = 9.6, 1.7 Hz, 1H), 7.66 (dt, *J* = 9.7, 1.0 Hz, 1H), 6.54 (d, *J* = 0.9 Hz, 1H), 4.10 (s, 1H), 2.95 (t, *J* = 7.1 Hz, 2H), 2.80 (td, *J* = 8.5, 3.9 Hz, 1H), 1.86–1.61 (m, 2H), 0.75–0.62 (m, 1H), 0.38–0.23 (m, 2H), 0.19–0.04 (m, 1H), 0.04 to −0.04 (m, 1H). ^13^C NMR (126 MHz, CD_3_OD): δ 163.34, 149.29, 142.36, 141.01, 137.79, 127.92, 126.88, 123.79, 123.18, 114.54, 101.68, 97.78, 74.80, 45.82, 44.70, 34.31, 17.16, 2.35, 1.43.

#### *N*-(2-(2-Isopropylazetidin-1-yl)methyl)-1*H*-pyrrolo[3,2-c]pyridin-6-yl)-[1,2,4] Triazolo[4,3-a]pyridine-6-carboxamide (**SR-C-107**) was Synthesized through the Same Method as **YR-D-120**

^1^H NMR (400 MHz, CD_3_OD): δ 9.22 (d, *J* = 0.6 Hz, 1H), 9.14–9.06 (m, 1H), 8.45 (t, *J* = 2.5 Hz, 1H), 8.06 (s, 1H), 7.92–7.83 (m, *J* = 9.6, 4.6, 1.7 Hz, 1H), 7.76 (d, *J* = 9.6 Hz, 1H), 6.40 (s, 1H), 3.91 (d, *J* = 13.7 Hz, 1H), 3.59 (t, *J* = 13.0 Hz, 1H), 3.19 (s, 1H), 2.96–2.80 (m, 2H), 2.06–1.94 (m, 1H), 1.82–1.58 (m, 2H), 0.89–0.80 (m, 3H), 0.75 (t, *J* = 6.1 Hz, 3H). ^13^C NMR (101 MHz, DMSO-*d*_6_): δ 162.98, 148.84, 145.00, 141.88, 140.21, 139.20, 138.07, 127.80, 127.57, 123.39, 121.91, 114.75, 99.24, 97.13, 72.49, 56.17, 50.75, 49.06, 40.60, 40.39, 40.18, 39.97, 39.76, 39.55, 39.34, 33.68, 20.92, 19.12, 17.95.HRMS (ESI+) was calcd for C_21_H_24_N_7_O [M + H]^+^, 390.2037; found, 390.2029. **SR-C-107 (R)** and **SR-C-107 (S)** were synthesized through the same method as **SR-C-107** by using **7a** and **7b**.

#### (*R*)-*N*-(2-((2-Isopropylazetidin-1-yl)methyl)-1*H*-pyrrolo[3,2-c]pyridin-6-yl)-[1,2,4]triazolo[4,3-a]pyridine-6-carboxamide (**SR-C-107 (R)**)

^1^H NMR (400 MHz, CD_3_OD): δ 9.32 (s, 1H), 9.19 (s, 1H), 8.54 (s, 1H), 8.16 (s, 1H), 7.96 (d, *J* = 9.7 Hz, 1H), 7.84 (d, *J* = 9.7 Hz, 1H), 6.49 (s, 1H), 4.02 (d, *J* = 13.7 Hz, 1H), 3.68 (d, *J* = 13.7 Hz, 1H), 3.30–3.28 (m, 1H), 3.10–2.89 (m, 2H), 2.17–2.06 (m, 1H), 1.94–1.81 (m, 1H), 1.80–1.66 (m, 1H), 0.95 (d, *J* = 6.6 Hz, 3H), 0.84 (d, *J* = 6.7 Hz, 3H). ^13^C NMR (101 MHz, DMSO-*d*_6_): δ 162.34, 148.19, 144.36, 141.23, 139.59, 138.41, 137.42, 127.14, 126.92, 122.73, 121.26, 114.10, 98.67, 96.48, 71.86, 55.45, 50.09, 33.01, 20.27, 18.46, 17.29. HRMS (ESI+) was calcd for C_21_H_23_N_7_O [M + H]^+^, 390.2000; found, 390.2029.

#### (*S*)-*N*-(2-((2-Isopropylazetidin-1-yl)methyl)-1*H*-pyrrolo[3,2-c]pyridin-6-yl)-[1,2,4]triazolo[4,3-a]pyridine-6-carboxamide (**SR-C-107 (S)**)

^1^H NMR (400 MHz, CD_3_OD): δ 9.32 (d, *J* = 0.8 Hz, 1H), 9.19 (t, *J* = 1.4 Hz, 1H), 8.54 (d, *J* = 1.1 Hz, 1H), 8.15 (t, *J* = 1.0 Hz, 1H), 7.97 (dd, *J* = 9.7, 1.6 Hz, 1H), 7.85 (dt, *J* = 9.6, 1.0 Hz, 1H), 6.48 (d, *J* = 0.9 Hz, 1H), 3.99 (d, *J* = 13.6 Hz, 1H), 3.65 (d, *J* = 13.7 Hz, 1H), 3.30–3.26 (m, 1H), 3.04–2.88 (m, 2H), 2.16–2.03 (m, 1H), 1.92–1.80 (m, 1H), 1.79–1.65 (m, 1H), 0.95 (d, *J* = 6.7 Hz, 3H), 0.83 (d, *J* = 6.7 Hz, 3H). ^13^C NMR (101 MHz, DMSO-*d*_6_): δ 163.47, 149.32, 145.50, 142.36, 140.72, 139.54, 138.55, 128.28, 128.05, 123.86, 122.39, 115.23, 99.80, 97.61, 72.99, 56.58, 51.22, 34.13, 21.40, 19.59, 18.42. HRMS (ESI+) was calcd for C_21_H_23_N_7_O [M + H]^+^, 390.2000; found, 390.2027.

(*R*)-*N*-(4-((2-Isopropylazetidin-1-yl)methyl)benzyl)-[1,2,4]triazolo[4,3-a]pyridine-6-carboxamide **13 (R)** and (*S*)-*N*-(4-((2-isopropylazetidin-1-yl)methyl)benzyl)-[1,2,4]triazolo[4,3-a]pyridine-6-carboxamide **13 (S)**, **7a** and **7b** were then used for the synthesis of **13 (R)** and **13 (S)** with reported method.

**13 (S)**, ^1^H NMR (400 MHz, CD_3_OD): δ 9.30 (s, 1H), 9.08 (t, *J* = 1.3 Hz, 1H), 7.95–7.66 (m, 2H), 7.49–7.26 (m, 4H), 4.63 (s, 2H), 4.61 (s, 1H), 4.08 (d, *J* = 12.7 Hz, 1H), 3.73 (d, *J* = 12.6 Hz, 1H), 3.32–3.16 (m, 2H), 2.30–2.12 (m,1H), 2.02–1.93 (m,1H), 1.90–1.80 (m,1H), 0.97 (d, *J* = 6.6 Hz, 3H), 0.86 (d, *J* = 6.7 Hz, 3H). ^13^C NMR (101 MHz, CD_3_OD): δ 164.76, 148.98, 137.50, 136.39, 129.21, 127.39, 127.36, 126.39, 122.45, 114.20, 73.27, 63.12, 49.79, 48.06, 47.85, 47.64, 47.42, 47.21, 47.00, 43.10, 34.48, 21.64, 18.38, 16.73. HRMS (ESI+) was calcd for C_21_H_25_N_5_O [M + H]^+^, 364.2000; found, 364.2122.

**13 (R)**, ^1^H NMR (400 MHz, CD_3_OD): δ 9.38–9.28 (m, 1H), 9.13 (d, *J* = 1.2 Hz, 1H), 8.00–7.80 (m, 2H), 7.59–7.43 (m, 4H), 4.68 (d, *J* = 4.0 Hz, 2H), 4.47 (d, *J* = 13.1 Hz, 1H), 4.36 (d, *J* = 13.1 Hz, 1H), 4.22–3.97 (m, 2H), 3.92–3.78 (m, 1H), 2.65–2.51 (m, 1H), 2.37–2.21 (m, 1H), 2.19–2.03 (m, 1H), 1.00–0.89 (m, 6H). ^13^C NMR (101 MHz, CD_3_OD): δ 164.86, 149.00, 139.59, 137.52, 129.97, 127.85, 127.40, 126.44, 122.37, 114.23, 74.49, 61.57, 49.58, 42.95, 32.88, 21.14, 17.75, 16.12. HRMS (ESI+) was calcd for C_21_H_25_N_5_O [M + H]^+^, 364.2000; found, 364.2124.

#### *tert*-Butyl (4-((4-Cyanophenyl)thio)butyl)carbamate (**17**)

4-((4-Aminobutyl)thio)benzonitrile (**16**) (2.43 mmol, 500 mg) was dissolved in ACN (25 mL) under nitrogen atmosphere. A solution of Boc_2_O (2.67 mmol, 581 mg) in dry ACN (25 mL) was added dropwise at 0 °C under stirring. The solution was then stirred overnight at room temperature. The solvent was removed under reduced pressure and the residue was then purified by column chromatography (silica gel, 10% EtOAc/hexane as the eluent) to yield **17** as a white solid (578 mg, 78%). ^1^H NMR (400 MHz, chloroform-*d*): δ 7.52 (d, *J* = 8.6 Hz, 1H), 7.29 (d, *J* = 8.6 Hz, 1H), 4.53 (s, 1H), 3.15 (q, *J* = 5.9 Hz, 2H), 2.99 (t, *J* = 7.1 Hz, 1H), 1.76–1.70 (m, 2H), 1.68–1.61 (m, 2H), 1.43 (s, 5H).

#### *tert*-Butyl (4-((4-(([1,2,4]Triazolo[4,3-a]pyridine-6-carboxamido)methyl)phenyl)thio)butyl) Carbamate (**18**)

LiAlH_4_ (2.08 mmol, 77 mg) was suspended in dry THF (10 mL) under nitrogen gas and was cooled under 0 °C. The solution of **17** (1.90 mmol, 578 mg) in dry THF (10 mL) was added dropwise at 0 °C under stirring. The solution was then stirred for 4 h at room temperature. Water (3 mL) was added dropwise, followed by 2 M aqueous NaOH solution (3 mL) and then water (3 mL). The precipitate was filtered and washed with THF. The combined filtrate was then dried with anhydrous Na_2_SO_4_ and evaporated in vacuo. The residue was directly dissolved in DMF (10 mL), then 1,2,4-triazolo[4,3-a] pyridine-6-carboxylic acid (2 mmol, 326 mg), DIPEA (4 mmol, 516 mg) and EDCI (2.4 mmol, 460 mg) were added. The resulting solution was stirred at room temperature overnight. Then, the solution was diluted with EtOAc (40 mL) and washed with saturated NaHCO_3_ solution (2 × 50 mL), 1 M HCl (2 × 50 mL), and saturated brine (50 mL). The organic layers were then dried with anhydrous Na_2_SO_4_ and then concentrated. The residue was purified by column chromatography (silica gel, 10% MeOH/DCM as the eluent) to yield **18** as a light-yellow solid (446 mg, 54%). ^1^H NMR (400 MHz, chloroform-*d*): δ 8.91 (d, *J* = 1.6 Hz, 1H), 8.77 (s, 1H), 7.59 (t, *J* = 8.2 Hz, 2H), 7.47–7.39 (m, 1H), 7.17 (s, 4H), 4.54 (d, *J* = 5.5 Hz, 3H), 4.24–4.15 (m, 5H), 3.07–2.93 (m, 2H), 2.82 (t, *J* = 6.7 Hz, 2H). HRMS (ESI): calcd for C_23_H_29_N_5_O_3_S [M + H]^+^, 456.2064; found, 456.2055.

#### *N*-(4-((4-(3-(5,5-Difluoro-7-(1*H*-pyrrol-2-yl)-5*H*-4l4,5l4-dipyrrolo[1,2-c:2′,1′-f][1,3,2]diazaborinin-3-yl)propanamido)butyl)thio)benzyl)-[1,2,4]triazolo[4,3-a]pyridine-6-carboxamide (**Tracer 1**)

To a solution of **18** (0.1 mmol, 46 mg) in 5 mL of 1,4-dioxane was added 5 mL of 4 M HCl solution in 1,4-dioxane. The resulting solution was stirred at room temperature for 2 h. Then, the reaction mixture was concentrated to dryness in vacuo. The residue was directly dissolved in anhydrous DMF (2 mL). **19** (0.10 mmol, 36 mg) and DMAP (0.20 mmol, 24 mg) were then added. The mixture was cooled to 0 °C and EDCI (0.15 mmol, 29 mg) was subsequently added. The resulting mixture was then stirred at room temperature for 36 h. The reaction mixture was finally purified by column chromatography (silica gel, 5% methanol/DCM as the eluent) to yield fluorescence **Tracer 1** as a purple solid (6.2 mg, 9.3%). 1H NMR (400 MHz, chloroform-*d*): δ 10.30 (s, 1H), 8.74 (d, *J* = 0.8 Hz, 1H), 8.71 (t, *J* = 1.4 Hz, 1H), 7.68–7.63 (m, 1H), 7.54–7.50 (m, 1H), 7.40–7.34 (m, 2H), 7.29 (s, 1H), 7.23–7.21 (m, 1H), 7.12 (d, *J* = 8.2 Hz, 1H), 7.08–7.02 (m, 2H), 6.94 (s, 1H), 6.89 (d, *J* = 4.7 Hz, 1H), 6.81 (d, *J* = 8.2 Hz, 1H), 6.75 (d, *J* = 4.0 Hz, 1H), 6.43–6.36 (m, 2H), 5.90 (t, *J* = 6.1 Hz, 1H), 4.56 (dd, *J* = 5.4, 3.5 Hz, 2H), 4.43 (d, *J* = 4.1 Hz, 2H), 3.31–3.18 (m, 2H), 2.97–2.86 (m, 2H), 2.74–2.55 (m, 2H), 1.61 (d, *J* = 6.8 Hz, 4H). ^11^B NMR (128 MHz, CDCl_3_): δ 1.42, (t, *J* = 36.5 Hz). HRMS (ESI): calcd for C_34_H_32_BF_2_N_8_O_2_S [M – H]^−^ 665.2425; found, 665.2452.

#### *tert*-Butyl (4-((3-(6-(Cyclobutylcarbamoyl)imidazo[1,2-a]pyridin-2-yl)phenyl)amino)-4-oxobutyl)carbamate (**21**)

To a solution of *N*-(*tert*-butoxy carbonyl)-4-aminobutyric acid (1.57 mmol, 225 mg) in anhydrous DMF (15 mL), DIPEA (3.15 mmol, 407 mg) was added. The solution was stirred for 30 min at room temperature and then HATU (1.25 mmol, 475 mg) and compound **20** (2.1 mmol, 530 mg) were added. The resulting solution was stirred at room temperature overnight. The reaction mixture was then diluted with EtOAc (50 mL) and then washed with saturated NaHCO_3_ solution (2 × 50 mL), 0.5 M HCl (2 × 10 mL), and brine (50 mL). The organic layers were dried with anhydrous Na_2_SO_4_ and concentrated in vacuo. The residue was then purified by column chromatography (silica gel, 5% methanol/DCM as the eluent) to yield **21** as a white solid (420 mg, 68%). ^1^H NMR (400 MHz, DMSO-*d*_6_): δ 9.99 (s, 1H), 9.07 (dd, *J* = 1.8, 1.0 Hz, 1H), 8.73 (d, *J* = 7.5 Hz, 1H), 8.43 (s, 1H), 8.27 (t, *J* = 1.9 Hz, 1H), 7.68 (dd, *J* = 9.5, 1.8 Hz, 1H), 7.64–7.56 (m, 3H), 7.36 (t, *J* = 7.9 Hz, 1H), 6.85 (t, *J* = 5.8 Hz, 1H), 2.98 (q, *J* = 6.6 Hz, 2H), 2.32 (t, *J* = 7.5 Hz, 2H), 2.24 (dd, *J* = 8.7, 2.9 Hz, 2H), 2.15–2.02 (m, 2H), 1.75–1.65 (m, 4H), 1.38 (s, 9H). ^13^C NMR (101 MHz, DMSO-*d*_6_): δ 171.47, 163.60, 156.09, 146.03, 145.37, 140.25, 134.39, 129.53, 128.69, 124.02, 120.91, 120.37, 119.15, 116.88, 116.11, 110.50, 77.93, 45.09, 30.56, 28.74, 26.05, 15.23. HRMS (ESI): calcd for C_27_H_34_N_5_O_4_ [M + H]^+^, 462.2606; found, 462.2596.

#### 2-(3-(4-Aminobutanamido)phenyl)-*N*-cyclobutylimidazo[1,2-a]pyridine-6-carboxamide (**22**)

To a solution of **21** (0.5 mmol, 245 mg) in 5 mL of 1,4-dioxane, 10 mL of 4 M HCl solution in 1,4-dioxane was added. The resulting solution was stirred at room temperature for 2 h. Then, the reaction mixture was concentrated to dryness in vacuo to yield **22** as a brown solid (178 mg, 91%).^1^H NMR (400 MHz, DMSO-*d*_6_): δ 10.42 (s, 1H), 9.34 (s, 1H), 9.09 (d, *J* = 7.4 Hz, 1H), 8.68 (s, 1H), 8.33 (t, *J* = 1.9 Hz, 1H), 8.16 (d, *J* = 9.3 Hz, 1H), 8.01 (s, 3H), 7.90 (d, *J* = 9.4 Hz, 1H), 7.71 (dt, *J* = 7.8, 1.3 Hz, 1H), 7.65 (dt, *J* = 8.2, 1.3 Hz, 1H), 7.50 (t, *J* = 7.9 Hz, 1H), 4.47 (p, *J* = 8.1 Hz, 1H), 2.87 (h, *J* = 5.9 Hz, 2H), 2.32–2.22 (m, 2H), 2.19–2.05 (m, 2H), 1.91 (p, *J* = 7.3 Hz, 2H), 1.72 (tt, *J* = 10.5, 5.1 Hz, 2H). ^13^C NMR (101 MHz, DMSO-*d*_6_): δ 171.27, 162.14, 141.72, 140.56, 137.85, 131.16, 130.29, 124.25, 117.18, 112.40, 112.07, 72.62, 70.98, 66.82, 63.26, 60.64, 45.32, 38.80, 33.50, 30.36, 23.47, 15.30. HRMS (ESI): calcd for C_22_H_26_N_5_O_4_ [M + H]^+^, 392.2082; found, 392.2075.

#### *N*-Cyclobutyl-2-(3-(4-(3-(5,5-difluoro-7-(1*H*-pyrrol-2-yl)-5*H*-4l4,5l4-dipyrrolo[1,2-c:2′,1′-f][1,3,2]diazaborinin-3yl)propanamido)butanamido)phenyl)imidazo[1,2-a]pyridine-6-carboxamide (**Tracer 2**) was Synthesized through the Same Method as **Tracer 1**

^1^H NMR (400 MHz, chloroform-*d*): δ 10.41 (s, 1H), 8.85–8.72 (m, 1H), 8.68 (s, 1H), 8.15 (s, 1H), 7.91 (s, 1H), 7.72 (d, *J* = 5.4 Hz, 1H), 7.60 (t, *J* = 8.3 Hz, 3H), 7.38 (d, *J* = 9.3 Hz, 2H), 7.12 (d, *J* = 3.6 Hz, 1H), 7.01 (d, *J* = 4.6 Hz, 1H), 6.96 (d, *J* = 4.4 Hz, 1H), 6.82 (dd, *J* = 8.3, 4.2 Hz, 2), 6.35–6.33 (m, 1H), 6.30–6.24 (m, 2H), 6.08 (s, 1H), 4.64–4.55 (m, 1H), 3.39–3.35 (m, 4H), 2.74 (t, *J* = 7.4 Hz, 2H), 2.49–2.41 (m, 2H), 2.32–2.24 (m, 2H), 2.00–1.94 (m, 2H), 1.87–1.77 (m, 4H). ^11^B NMR (128 MHz, CDCl_3_): δ 1.54 (t, *J* = 36.7 Hz). HRMS (ESI): calcd for C_38_H_38_BF_2_N_8_O_3_ [M + H]^+^, 703.3123; found, 703.3109.

### Crystal Structure Determination

A single crystal of **5a** (CCDC 2361323)/**5b** (2361324) was prepared by recrystallization in hexane. A suitable crystal with dimensions 0.12 × 0.07 × 0.03 mm^3^/0.12 × 0.12 × 0.02 mm^3^ was selected and mounted on a MITIGEN holder on a XtaLAB Synergy X-ray diffractometer. The crystal was kept at a steady *T* = 100.00(10) K during data collection. The structure was solved using the ShelXT 2018/2 solution program by dual methods and by using Olex2 1.5 as the graphical interface. The model was refined with ShelXL 2019/1 using full matrix least-squares minimization on *F*^2^.

#### 5a

C_13_H_17_NO_2_, *M*_r_ = 219.27, orthorhombic, *P*2_1_2_1_2_1_ (no. 19), *a* = 5.57030(10) Å, *b* = 9.88460(10) Å, *c* = 22.1842(2) Å, *a* = *b* = *g* = 90°, *V* = 1221.47(3) Å^3^, *T* = 100.00(10) K, *Z* = 4, *Z*′ = 1, *m* (Cu Kα) = 0.642, 24,286 reflections measured, 2633 unique (*R*_int_ = 0.0390) which were used in all calculations. The final w*R*_2_ was 0.0733 (all data) and *R*_1_ was 0.0275 (*I* ≥ 2*s*(*I*)).

#### 5b

C_13_H_17_NO_2_, *M*_r_ = 219.27, orthorhombic, *P*2_1_2_1_2_1_ (no. 19), *a* = 5.57010(10) Å, *b* = 9.88810(10) Å, *c* = 22.1770(2) Å, *a* = *b* = *g* = 90°, *V* = 1221.46(3) Å^3^, *T* = 100.00(10) K, *Z* = 4, *Z*′ = 1, *m* (Cu Kα) = 0.642, 12,058 reflections measured, 2298 unique (*R*_int_ = 0.0211) which were used in all calculations. The final w*R*_2_ was 0.0598 (all data) and *R*_1_ was 0.0228 (*I* ≥ 2*s*(*I*)).

### AlphaScreen Assay with Biotinylated-ENL-S1

The AlphaScreen assay was carried out in 384-well plates. Manual assay setup was performed in a 40 μL reaction buffer (50 mM HEPES pH 7.4, 100 mM NaCl, 0.1% bovine serum albumin, and 0.05% CHAPS) with final concentrations of 100 nM His-ENL YEATS, 100 nM Biotin-ENL-S1, and 20 μg/mL of AlphaScreen donor and acceptor beads. The protein, peptide, and compounds were mixed and incubated for 1 h at room temperature and then incubated for 30 min in dark after adding the α beads. AlphaScreen-signals were detected by a multimode microplate reader (BioTeK Synergy Neo2) equipped with an Alpha-laser (PerkinElmer).

### Biolayer Interferometry

Superstreptavidin biosensors were first incubated in an assay buffer (20 mM Tris, pH 7.8, 300 mM NaCl, 0.05% Tween 20, 0.1% DMSO) for 15 min. Sensors were coated with 40–60 μg/mL of biotinylated ENL for 15 min at room temperature. Inhibitors were serially diluted across a 96-well plate in a volume of 200 μL. Association of samples to either ENL- or uncoated reference sensors was measured over 300 s and dissociation over 300 s with baseline measurements (buffer only) for 300 s. All assays were run with continuous 1000 rpm shaking. Data were analyzed using the OctetRED data analysis software with reference subtraction in which reference uncoated sensors and reference samples were subtracted. Then, global kinetic fit was performed for all sonogram curves with a 1:1 model to get *K*_D_ values.

### Log *D* at pH 7.4

Log *D* at 7.4, which is a partition coefficient between *n*-octanol and aqueous buffer pH 7.4 of the compounds was measured on the chromatographic procedure whose condition was developed based on a published method.^35^

### Cell Culture

MV4-11 (IMDM supplemented with 10% nonheat inactivated FBS, penicillin, and streptomycin), MOLM-13 (RPMI 1640 supplemented with 10% heat inactivated FBS, penicillin, and streptomycin), JURKAT (RPMI 1640 supplemented with 10% heat inactivated FBS, penicillin, and streptomycin), HEK 293T (DMEM supplemented with 10% heat inactivated FBS, penicillin, and streptomycin) were maintained in a humidified 37 °C incubator with 5% CO_2_. Cells were obtained from ATCC.

### Generation of Stable Nluc-ENL YEATS Expressed HEK293T Cell Line

DNA encoding the ENL YEATS domain (aa 1–148) was cloned into an NLuc containing plasmid pCDH-EF1-NL uc TAA stop codon to afford pCDH-EF1-Nluc-ENL YEATS. The plasmid for lentivirus packaging was prepared using the EndoFree Plasmid Midi Kits (Omega, D6915-03) according to the manufacturer’s protocol. For packaging NLuc-ENL YEATS lentivirus particles, HEK293T/17 cells were cultured in 10 cm^2^ dishes to 70–80% confluency and then cotransfected with three plasmids pCDH-EF1-NLuc-ENL, psPAX2 and PMD2.G using polyethylenimine as described previously.^48^ The expression of NLuc-ENL YEATS was then confirmed by immunoblotting using an NLuc luciferase antibody (R&D, MAB10026-SP).

### NanoBRET Assay. Apparent Tracer Affinity

Approximately 1.8 × 10^4^ HEK293T cells (100 μL) stably expressing NLuc-ENL YEATS were seeded into a white nonbinding 96-well assay plate and incubated overnight at 37 °C in a humidified 5% CO_2_ atmosphere. After removal of the medium, the cells were added with a tracer (with or without excess of unlabeled compound) in the Opti-MEM with 0.1% DMSO. After incubation at 37 °C for 2 h the assay plate was equilibrated at room temperature for 15 min. For BRET detection, the NanoGlo Substrate and the extracellular NanoLuc inhibitor (Promega, N2160) were diluted with Opti-MEM as described in protocol to afford detection solution. 50 μL of the detection solution was added per well and the plate was incubated for 3 min at room temperature. The donor emission was measured at 450 nm and the acceptor emission at 610 nm using a BioTek SYNERGY neo2 multimode reader. The BRET ratio was calculated according to the following formula: BRET ratio = [(acceptor_sample_/donor_sample_) – (acceptor_no-tracer control_/donor_no-tracer control_)] × 1000.

### ENL Inhibitor Competitive Assay

Sample procedures were followed for cell seeding as described in the previously described tracer affinity assay. After removal of the medium, the cells were added with a tracer in the medium (1.1 μM, 100 μL) and varied concentrations of a test compound in 0.1% DMSO (10 μL). The plate was thoroughly mixed on an orbital shaker for 15 s at 900 rpm. Then the cells were incubated at 37 °C, 5% CO_2_ for 2 h for BRET ratio detection as described previously.

### *In Vitro* Stability Assay in Human Plasma

The *in vitro* stability analysis of a test compound was initiated by the addition of the test compound to 90 μL of prewarmed (37 °C) human plasma with a final concentration of 5 μM. All assays were performed in a plate shaker at 37 °C and conducted in triplicate. At 0, 5, 15, 30, 60, and 120 min, 400 μL acetonitrile (with the internal standard diclofenac as 10 μg/mL) was added to deproteinize the plasma and terminate the reaction, the remaining compound was then analyzed by HPLC-MS/MS.

### *In Vitro* Metabolic Stability Assay in HLM

The *in vitro* metabolic stability profile of an ENL inhibitor, including CL_int, pred_ and *t*_1/2_ was determined by the estimation of the remaining compound concentration after incubation with HLM (0.5 mg·mL^–1^), NADPH (cofactor, 1 mM), and MgCl_2_ (5 mM) in a 0.1 M phosphate buffer (pH 7.4). At 0, 5, 15, 30, 45, and 60 min, 200 μL acetonitrile (with the internal standard diclofenac as 10 μg/mL) was added to terminate the reaction, the remaining compound was analyzed by HPLC-MS/MS.

### Cell Toxicity Assay

Approximately 1 × 10^4^ JURKAT, MOLM-13, MV4-11 and HEK293T cells in RPMI 1640 (no phenol red) (100 μL) were added to a 96-well plate and treated with DMSO or a compound at indicated concentrations for 72 h or 8 days. Cell viability was measured using the CCK-8 kit (Abcam, ab228554) according to the manufacturer’s instructions.

### Anti-Proliferation Assay

Cell proliferation assays were carried out in a 96-well tissue culture plate at 2 × 10^4^ cells/well for all cell lines except in 200 μL with a compound added as 1:1000 dilution of DMSO stocks in triplicate. Culture density was determined every 3–4 days using the countess automated cell counter, after which 2 × 10^4^ live cells were reseeded in fresh media and compound. The cumulative cell count was achieved by back calculation.

### Cell Cycle Analysis

For cell cycle analysis, 5 × 10^6^ cells were cultured at 2 mL/well in a 6-well plate treated in a ratio of 1:1000 with compound stocks in DMSO or 1:1000 with DMSO in triplicate. Cell cycle staining was performed by using a cell cycle analysis kit (Abcam, ab287852) and treated according to the protocol provided. The signal was analyzed by flow cytometry and the cytometry results were analyzed by CytExpert software.

### Cellular Thermal Shift Assay

MOLM-13 and MV4-11 were incubated with 10 μM of inhibitor **13** for 3 and 6 h, respectively. The cells were then collected and washed with PBS 3 times. The cell pellets were resuspended in PBS-containing protease inhibitors and aliquoted into PCR microtubes (approximately 3 million cells in 54 μL). Cells were heated at indicated temperatures for 3 min in a thermal cycler (Bio-Rad) and then incubated at room temperature for 2 min. A total of 6 μL of 10× cell lysis buffer (8% NP-40, 50% glycerol, and 10 mM dithiothreitol) was added to each sample before subjecting the sample to three freeze–thaw cycles by liquid nitrogen and 37 °C water bath incubations to lyse the cells. The cell lysates were centrifuged at 13,000 rpm at 4 °C for 10 min, and the supernatants were analyzed using SDS-PAGE and Western blotting.

### RNA Extraction and qRT-PCR

Total RNA was extracted using the RNeasy plus kit (Qiagen, 74034) and reverse-transcribed using an iScript cDNA synthesis kit (Bio-Rad, 1708890). Quantitative real-time PCR (qRT-PCR) analyses were performed as described previously using PowerUp SYBR Green PCR Master Mix (Thermo Fisher, A25742) and the Bio-Rad CFX96 real-time PCR detection system. Gene expressions were calculated following normalization to β2-microglobulin (B2M) levels using the comparative Ct (cycle threshold) method. The primer pairs are as follows. HOXA9: forward 5′-GTATAG-GGGCACCGCTTTTT-3′, reverse 5′-AATGCTGAGAATGAGAGCGG-3′. MEIS1: forward 5′-CACGCTTTTTGTGACGCTT-3′, reverse 5′ GGACAACAGCAGTG-AGCAAG-3′. MYB: forward 5′-GATGTGTGACCATGACTATG-3′, reverse 5′-GCACTGCACATCTGTTCGAT-3′, MYC: forward 5′-CACCGAGTCGTAGTCG-AGGT-3′, reverse 5′-TTT-CGGGTAGTGGAAAACCA-3′. B2M: forward 5′-AATGTCGGATGGATGAAACC-3′ reverse 5′-TAGCTGTGCTCGCGCTACT-3′.

### PK Analysis

Male CD-1 mice were used in the PK study. A compound was dissolved in a mixed solution containing 75% PEG300 and 25% D5W (5% dextrose in distilled water) or another solution containing 0.5% methyl cellulose and 0.5% Tween 80 in water at indicated dosage for i.v. and p.o. administrations, respectively. Three mice were used for each administration. Blood samples were taken from a vein at different time points up to 24 h after dosing, collected in tubes coated with an anticoagulant, and centrifuged at 1.5 × 10^4^ g for 5 min to obtain plasma samples. Acetonitrile-containing internal standard (Labetalol, 100 ng/mL) was added to the plasma to precipitate proteins. The samples were subjected to vortex mixing for 10 min and then centrifugation at 4 °C for 15 min at 3220*g*. Then clear supernatants were analyzed by HPLC-MS/MS.

### Antitumor Analysis in Xenografted Mice

Animal studies were approved by the Texas A&M’s Institutional Animal Care and Use Committees. The 5 week-old female NSG mice (NOD.Cg-PrkdcscidIl2rgtm1Wjl/SzJ, Jackson Laboratory #005557) were ordered and kept under pathogen-free conditions for 1 week with free access to food and water after arrival for acclimation. Tumor models in these mice were then built by engrafting 1 × 10^6^ MOLM-13-Luc cells (BPS Bioscience, 78372) through tail vein injection. Tumors were allowed to grow for 6 days, and then applied for treatment by oral gavage daily (day 0 was the beginning of treatment). Dosage of 200 or 400 mg/kg (vehicle of 5% DMSO, 45% PEG400, 2.5% Tween 80, and 47.5% water) was applied. Tumor growth was evaluated by bioluminescent imaging (BLI) using an IVIS *In Vivo* Imaging System by intraperitoneal injection of 150 mg/kg D-Luciferin potassium salt (MedChemExpress, HY-12591B). Their body weights were recorded every 2–3 days. Surviving mice were counted every day and the mortality was calculated. No animal was excluded from any of the analyses. The investigators were not blinded to allocation during experiments and outcome assessment.

## Supporting Information Available

(i) Binding affinity data for inhibitor **13**, **YR-D-120** and **YR-D-121**, (ii) validation data of NanoBRET assay, (iii) *in vitro* microsome stability data, (iv) biological data, (v) ^1^H NMR, ^13^C NMR and HPLC profile of compound, and (vi) crystal data of compound **5a** and **5b** (PDF)
Molecular formula strings (CSV)
X-ray identification of IV A S-isomer (PDF)
X-ray identification of IV B R-isomer (PDF)

## Abbreviations

AML: acute myeloid leukemia
BLI: biolayer interferometry
CETSA: cellular thermal shift assay
ENL: eleven-nineteen-leukemia
MLL: mixed-lineage leukemia gene
NADPH: nicotinamide adenine dinucleotide phosphate hydrogen
PADLE: phage-assisted active site-directed ligand evolution
PK: pharmacokinetics
SEC: super elongation complex
SSA: super streptavidin
SuFEx: sulfur(VI) fluoride exchange
TGI: tumor growth inhibition
