# Supplementary material for: Prioritization of Eleven-Nineteen-Leukemia Inhibitors as Orally Available Drug Candidates for Acute Myeloid Leukemia
Source: J Med Chem. 2024 Nov 12;67(22):20100–17. doi: 10.1021/acs.jmedchem.4c01337 (PMC11613437; doi:10.1021/acs.jmedchem.4c01337)
Supplement: Supplementary file 1 — jm4c01337_si_001.pdf [file jm4c01337_si_001.pdf]

Supporting Information

for

Prioritization of Eleven-Nineteen-Leukemia Inhibitors as Orally Available Drug Candidates for Acute Myeloid Leukemia

Xuejiao Shirley Guo[a, †], Sandeep Atla [a, †], Satyanarayana Nyalata[a], Yugendar R. Alugubelli[a], Peng-Hsun Chase Chen[a], Shiqing Xu[a,b,*], Wenshe Ray Liu[a,b,c,d,e,*]

[a]Texas A&M Drug Discovery Center, Department of Chemistry, Texas A&M University, College Station, TX 77843, USA

[b]Department of Pharmaceutical Sciences, Irma Lerma Rangel College of Pharmacy, Texas A&M University, College Station, TX 77843, USA

[c]Institute of Biosciences and Technology and Department of Translational Medical Sciences, College of Medicine, Texas A&M University, Houston, TX 77030, USA

[d]Department of Biochemistry and Biophysics, Texas A&M University, College Station, TX 77843, USA

[e]Department of Cell Biology and Genetics, College of Medicine, Texas A&M University, Bryan, TX 77807, USA

[†]Contributed equally to the paper.

*Correspondence should be addressed to Shiqing Xu: shiqing.xu@tamu.edu and Wenshe Ray Liu: wsliu2007@tamu.edu

Table of Contents

Figure S1. Disrupting the interactions between ENL YEATS domain and acetylated histones inhibit leukemia progress.. .... S3

Figure S2. Alpha profile for two inhibitor **13** isomers. S4

Figure S3. The binding kinetics of **YR-D-120** and **YR-D-121** to ENL YEATS were characterized with Biolayer Interferometry with varying concentrations inhibitors. S5

Figure S4. Expression of Nluc-ENL in Nluc-ENL+ stable HEK293T cells and WT HEK293T cell. S6

Figure S5. (a) Apparent affinity of **Tracer 1** for NLuc-ENL YEATS fusion protein in HEK293T cells. (b) NanoBRET curves of ENL inhibitor **24** in NLuc-ENL YEATS$^+$ HEK293T cells in the presence of various concentrations of **Tracer 2** and corresponding IC_{50} values.. S7

Figure S6. ln% remaining of ENL inhibitors in human liver microsome in the absence of NADPH. S8

Figure S7-S8. Biological data S9

Figure S9-S42. ^{1}H NMR, ^{13}C NMR and HPLC profile of compound. S11

Figure S43-S44. Crystal Data of compound **5a** and **5b**. S50

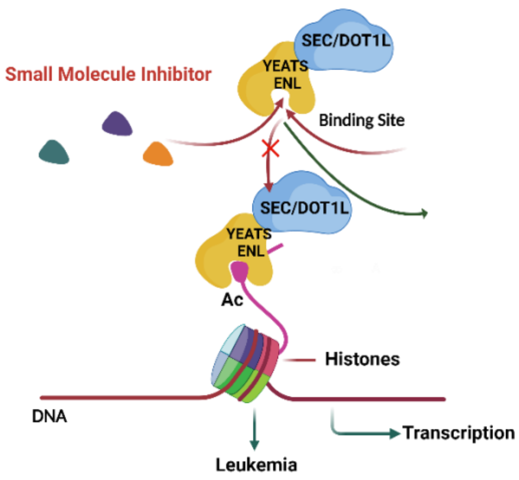

Figure S1. Disrupting the interactions between ENL YEATS domain and acetylated histones inhibit leukemia progress (Created with BioRender.com).

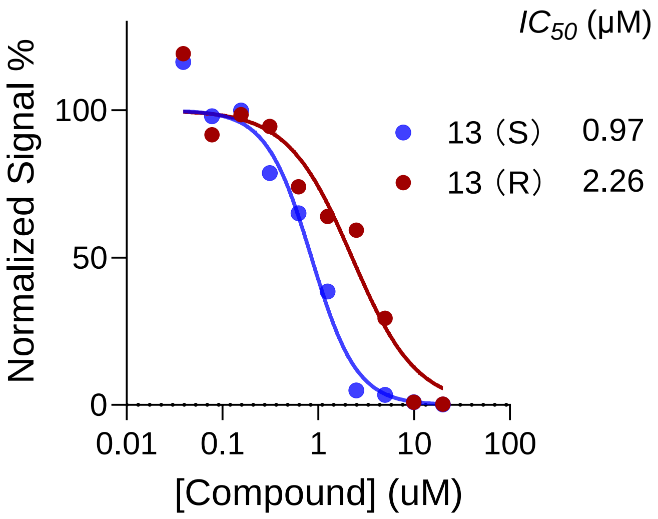

Figure S2. Alpha profile for two inhibitor **13** isomers.

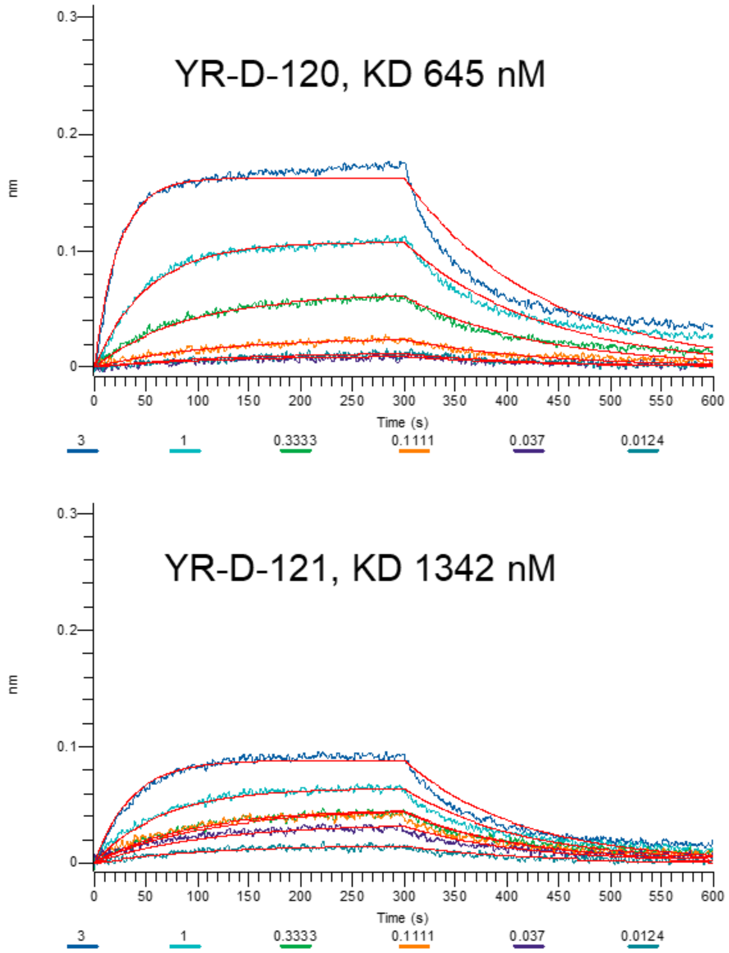

Figure S3. The binding kinetics of **YR-D-120** and **YR-D-121** to ENL YEATS were characterized with Biolayer Interferometry with varying concentrations inhibitors.

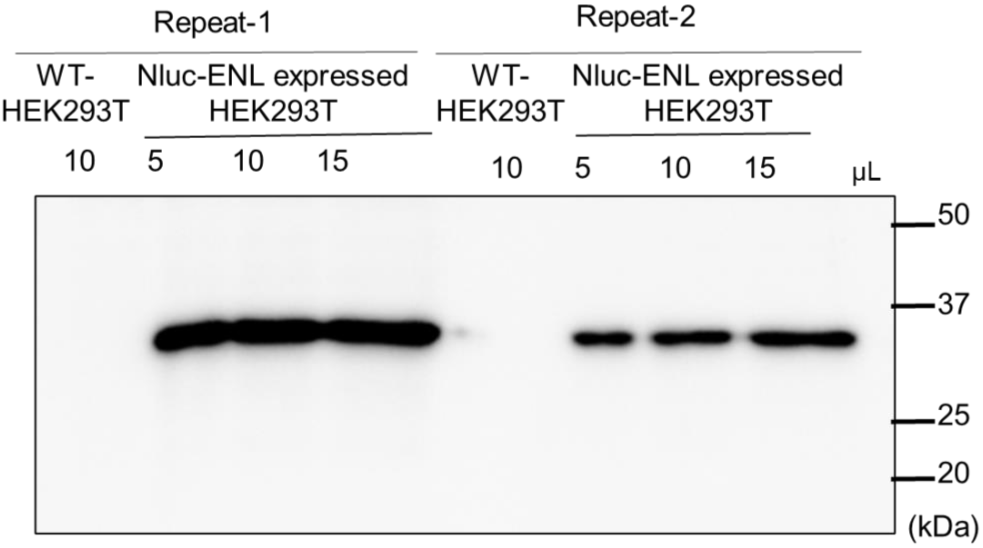

Figure S4. Expression of Nluc-ENL in Nluc-ENL+ stable HEK293T cells and WT HEK293T cell.

(a)

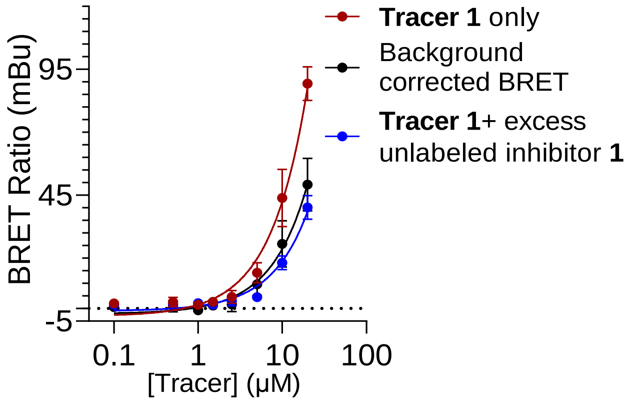

(b)

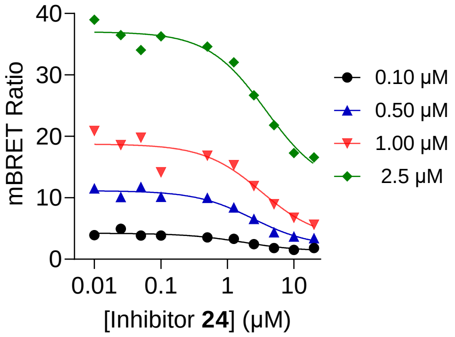

Tracer (μM)	IC_{50} (μM)
0.10	1.74
0.50	2.43
1.00	3.25
2.50	3.78

Figure S5. (a) Apparent affinity of **Tracer 1** for NLuc-ENL YEATS fusion protein in HEK293T cells. (b) NanoBRET curves of ENL inhibitor **24** in NLuc-ENL YEATS^{+} HEK293T cells in the presence of various concentrations of **Tracer 2** and corresponding IC_{50} values.

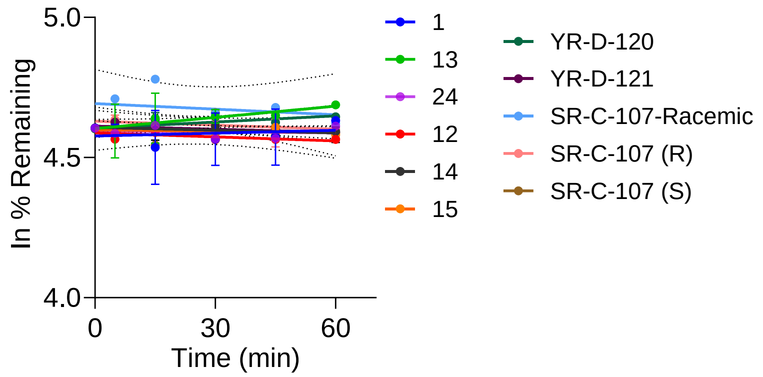

Figure S6. ln% remaining of ENL inhibitors in human liver microsome in the absence of NADPH.

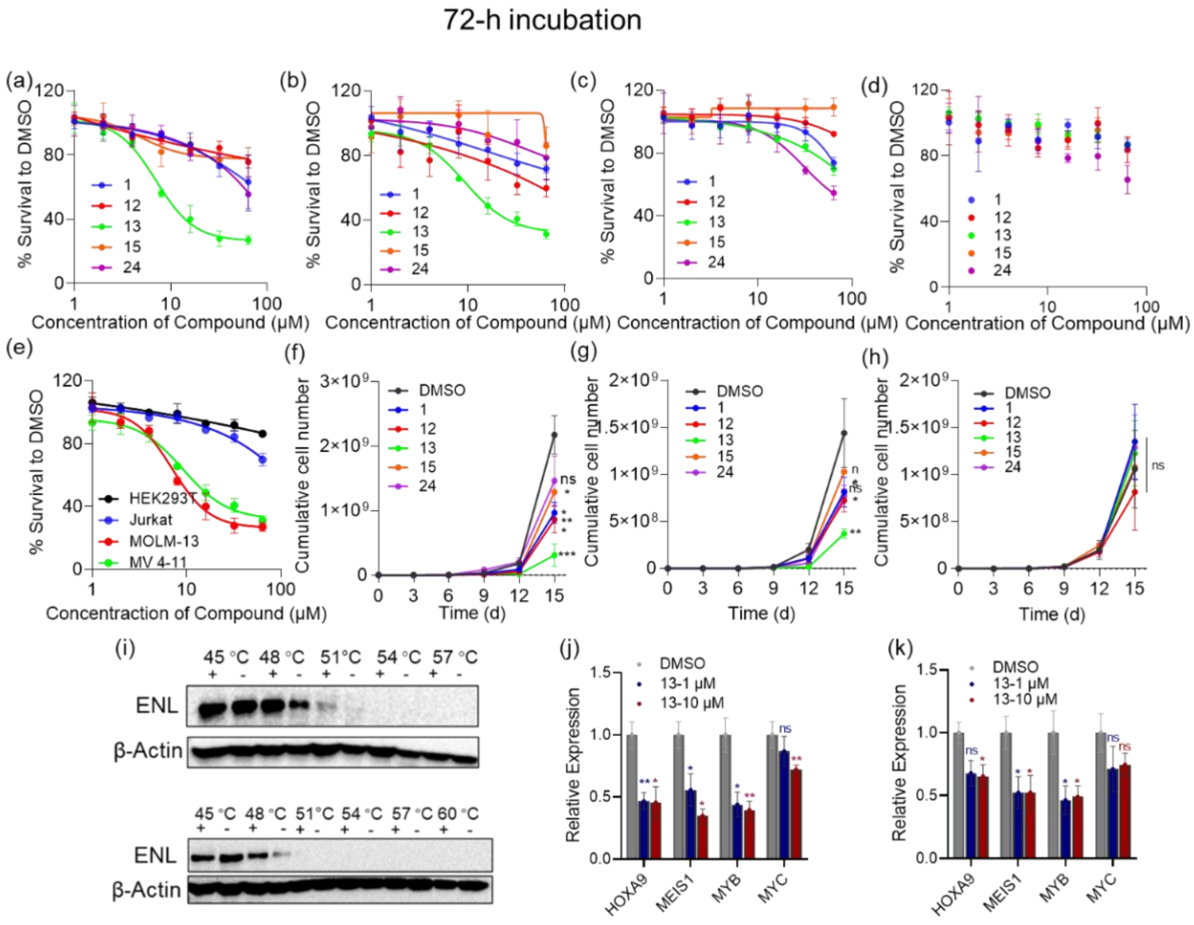

Figure S7. (a) MOLM-13, (b) MV4-11, (c) Jurkat, and (d) HEK293T cell viability post 72-h treatment with ENL inhibitors. (e) Comparison of cell viability for MOLM-13, MV4-11, Jurkat, and HEK293T cells post 72-h treatment with inhibitor **13**. Proliferation of (f) MOLM-13, (g) MV4-11, and (h) Jurkat cells in the presence of 10 μM of inhibitor **13**. (i) CETSAs of ENL in MOLM-13 (top) and MV4-11 (bottom) cells treated with 10 μM of inhibitor **13** (+) or the DMSO control (-) at indicated temperatures. *β*-Actin was used as a loading control. qRT-PCR analysis of HOXA9, MEIS1, MYB, and MYC gene expressions in (i) MOLM-13 and (k) MV4-11 cells treated with inhibitor **13** or the DMSO negative control. *$P < 0.05$, **$P < 0.01$, ***$P < 0.001$, ****$P < 0.0001$. Not significant (n.s.) $P > 0.05$.

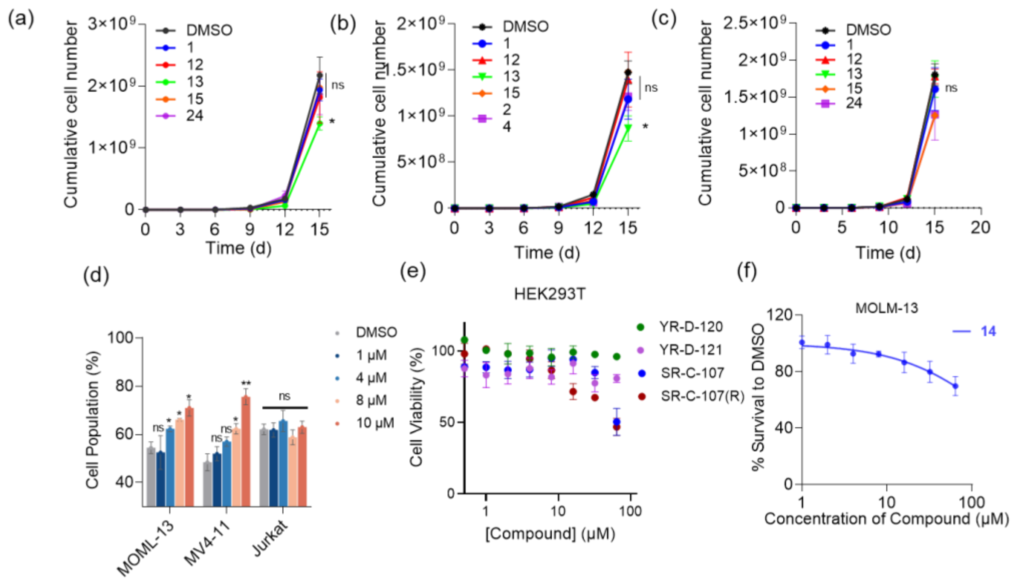

Figure S8. Proliferation of (a) MOLM-13, (b) MV4-11 and (c) Jurkat cell lines in response to different ENL inhibitors at 1 μM. (d) Comparison of percentage of G1 phase cells for different cell lines post 72 h treatment with different concentrations of inhibitor **13**. (e) HEK293T cell viability post 72-h treatment with ENL inhibitors. (f) Cell viability of MOLM-13 after 72-h treatment with compound 14. * $P < 0.05$, ** $P < 0.01$, Not significant (n.s.) $P > 0.05$.

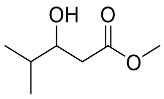

3

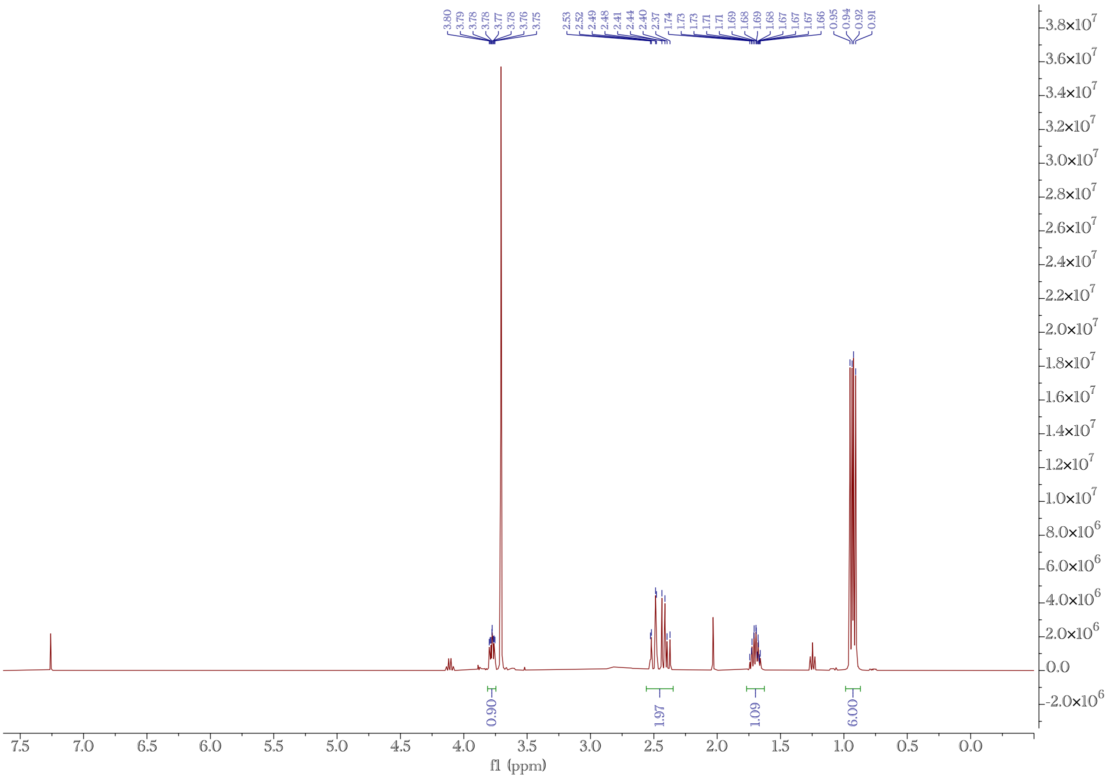

Figure S9. ^{1}H NMR of **3** in Chloroform-d.

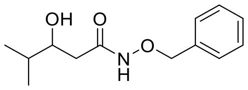

4

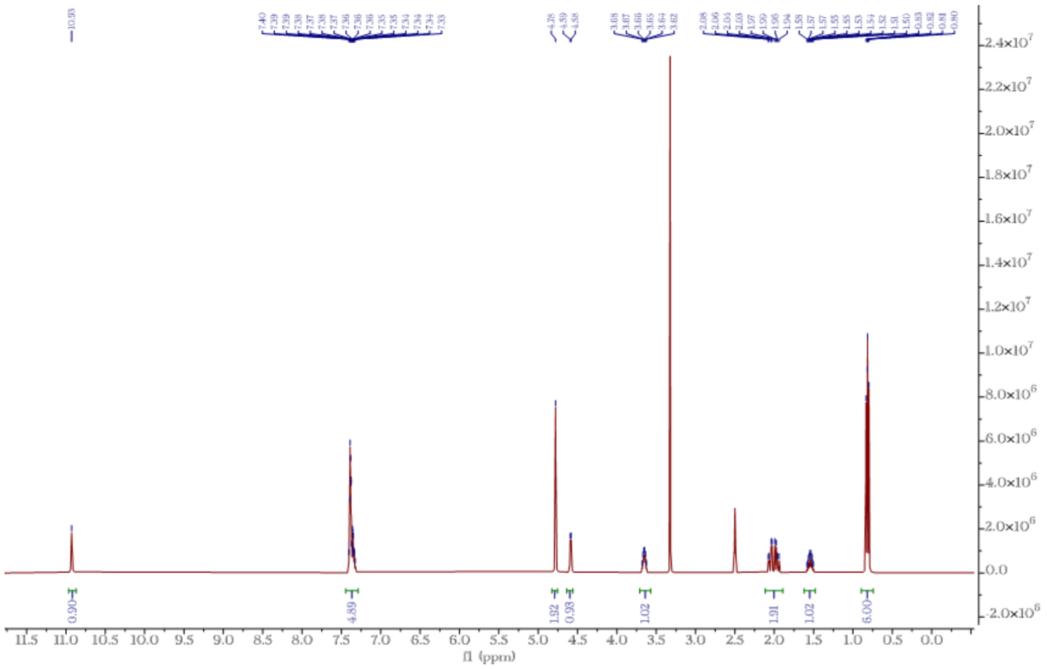

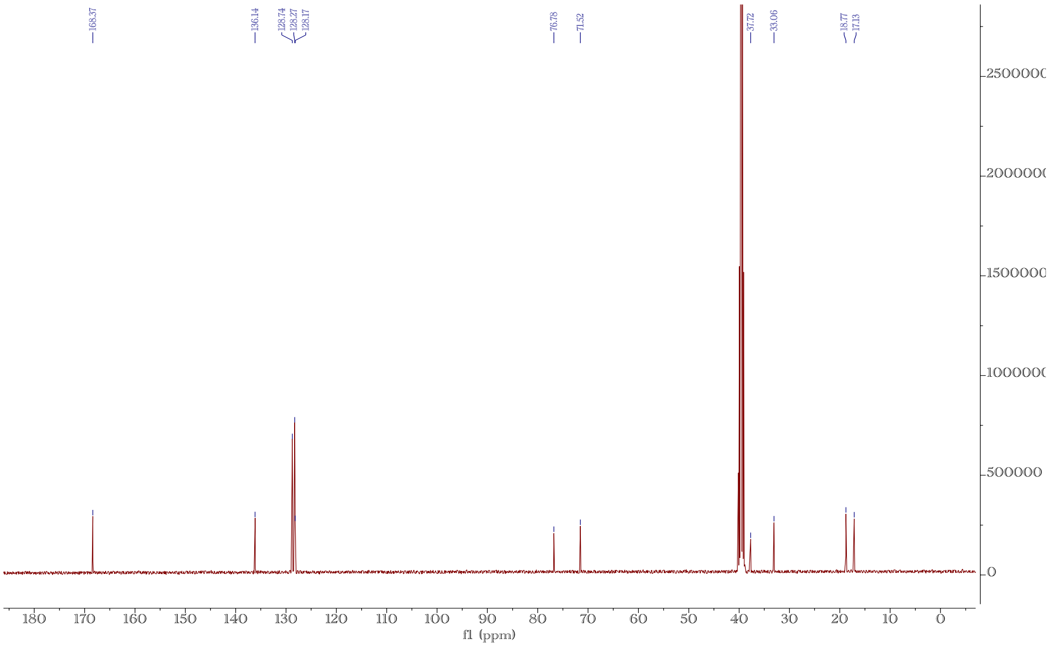

Figure S10. ^{1}H and ^{13}C NMR of **4** in DMSO-d_6.

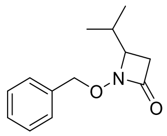

5

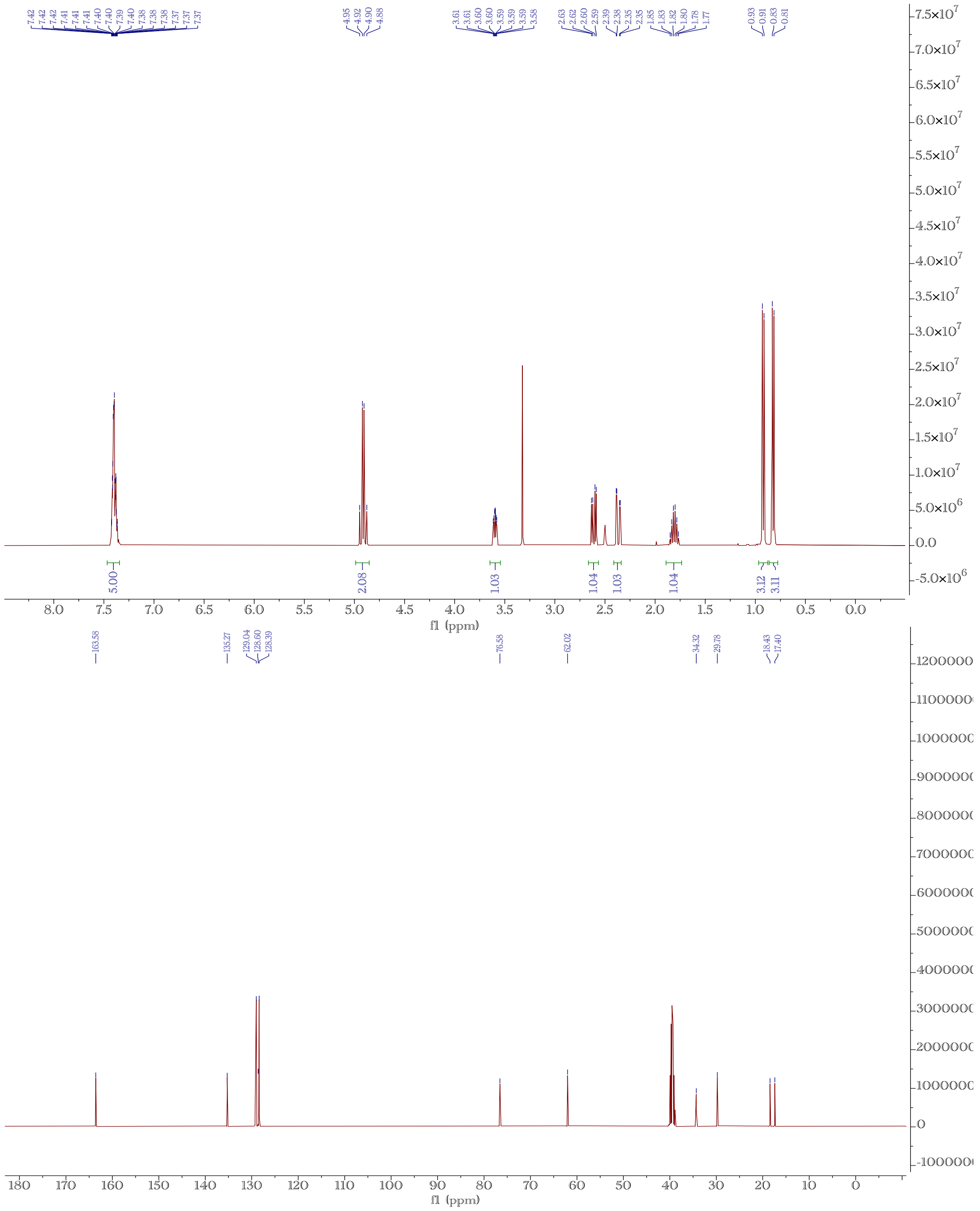

Figure S11. ^{1}H and ^{13}C NMR of **5 racemate** in DMSO-d_6.

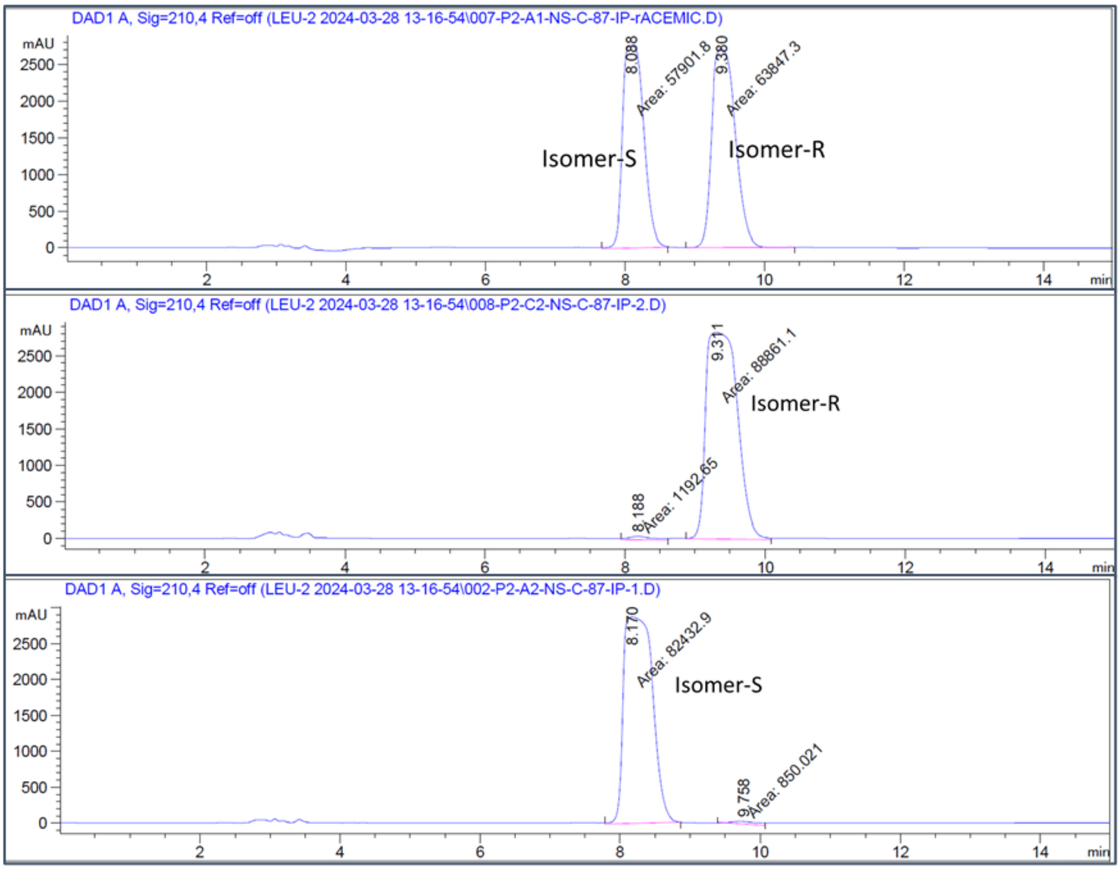

Figure S12.Chiral HPLC profile of **5, 5a (Isomer-S)** and **5b (Isomer-R).**

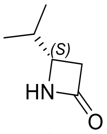

6a

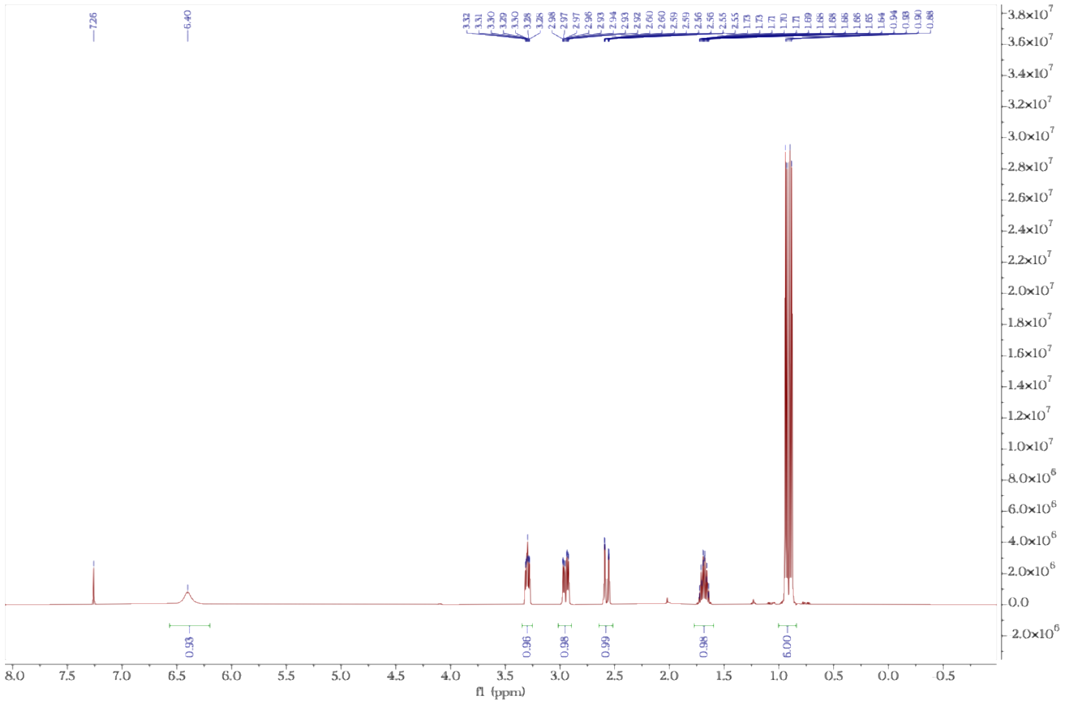

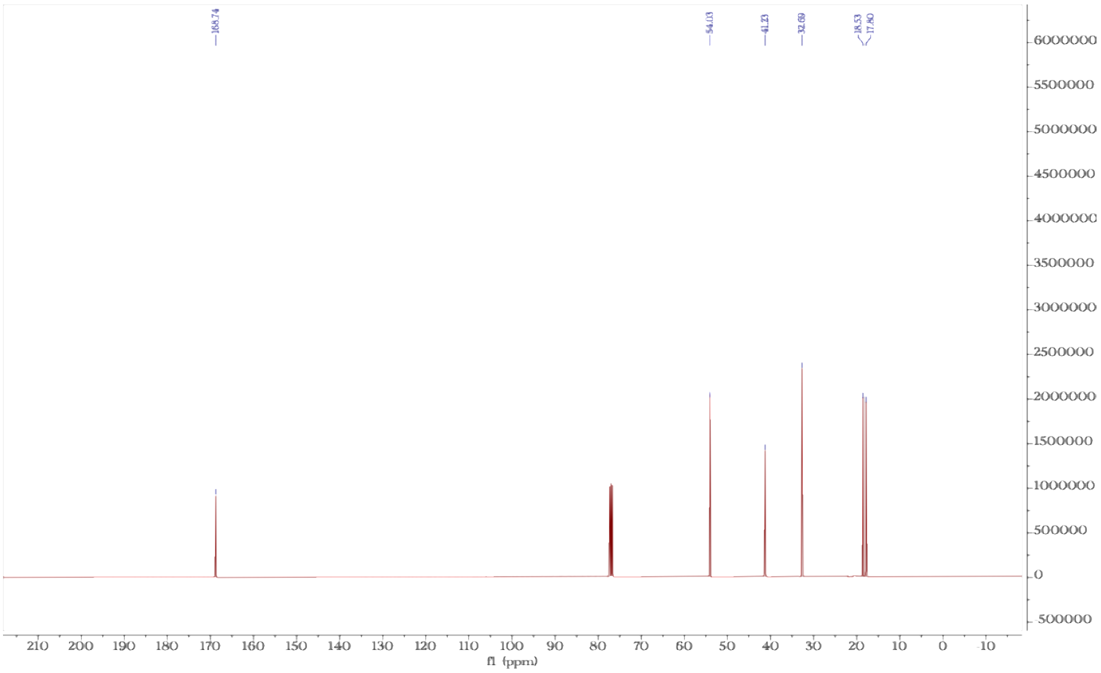

Figure S13. ^{1}H and ^{13}C NMR of **6a** in Chloroform-d.

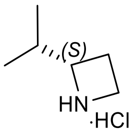

7a

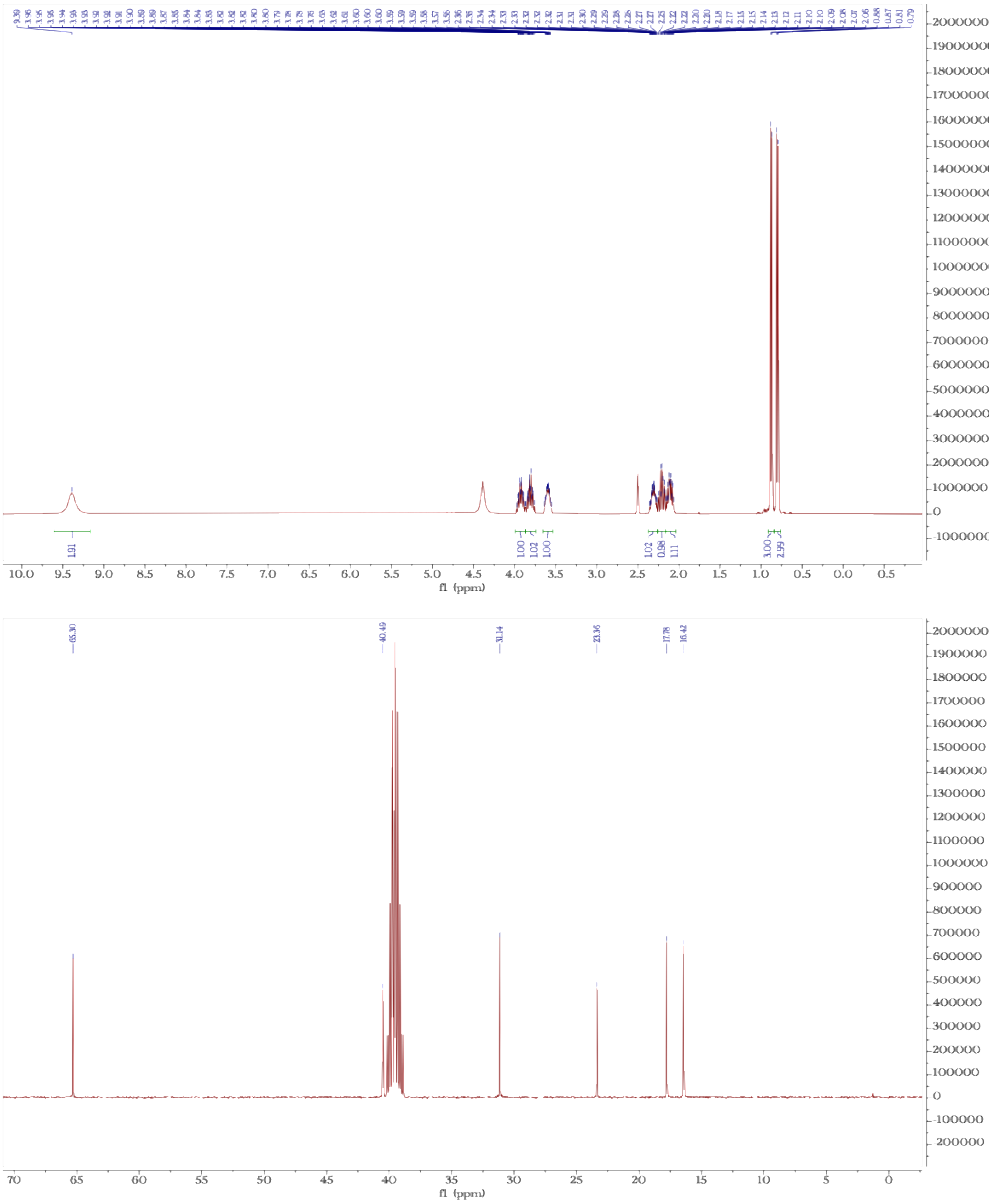

Figure S14. ^{1}H and ^{13}C NMR of **7a** in DMSO-d_6.

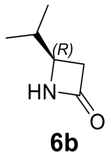

6b

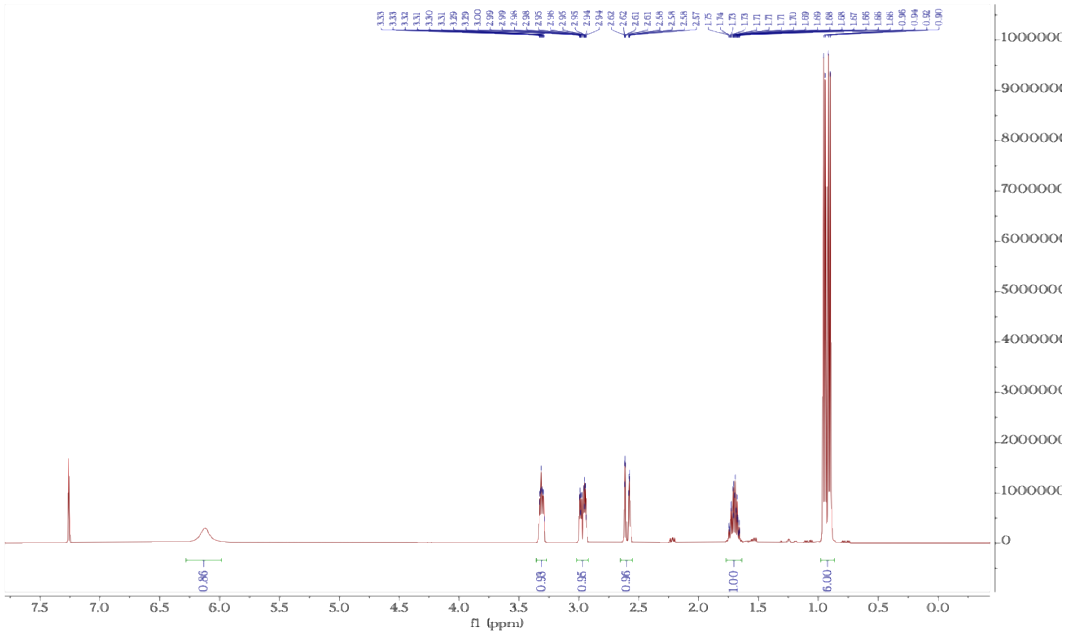

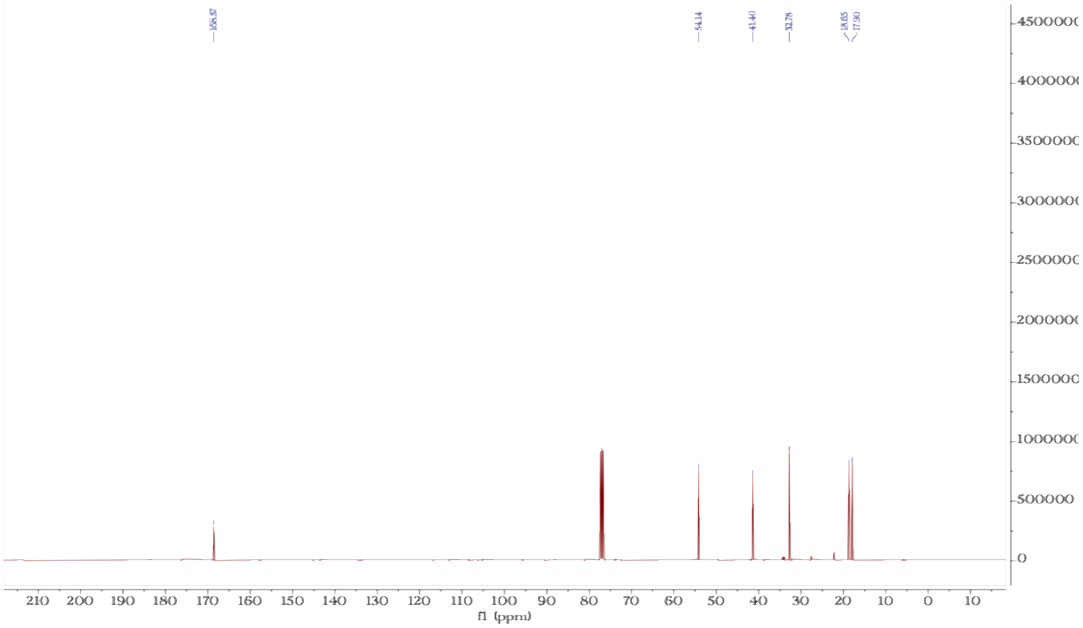

Figure S15. ^{1}H and ^{13}C NMR of **6b** in Chloroform-d.

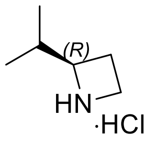

7b

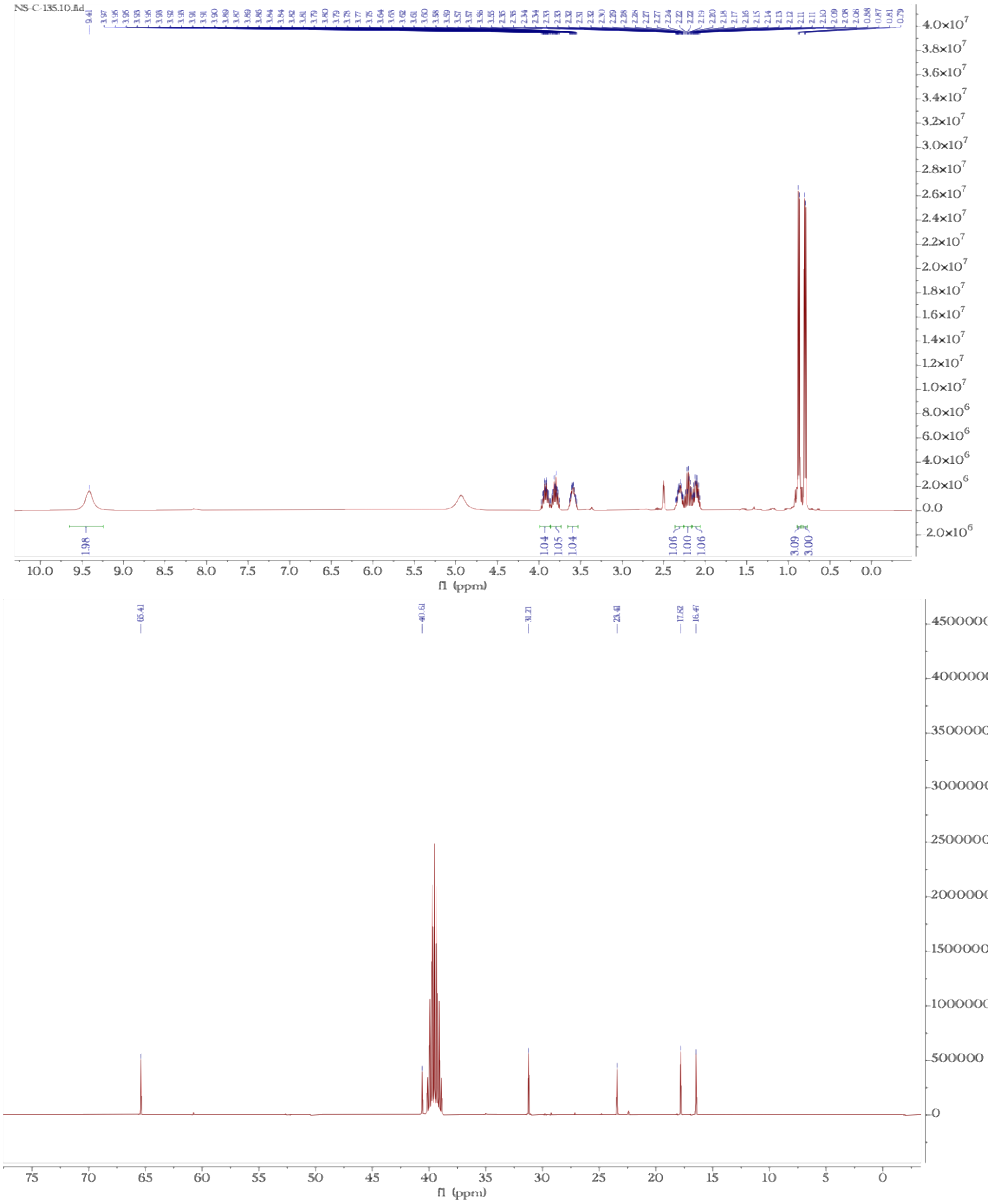

Figure S16. ^{1}H and ^{13}C NMR of **7b** in DMSO-d$_6$.

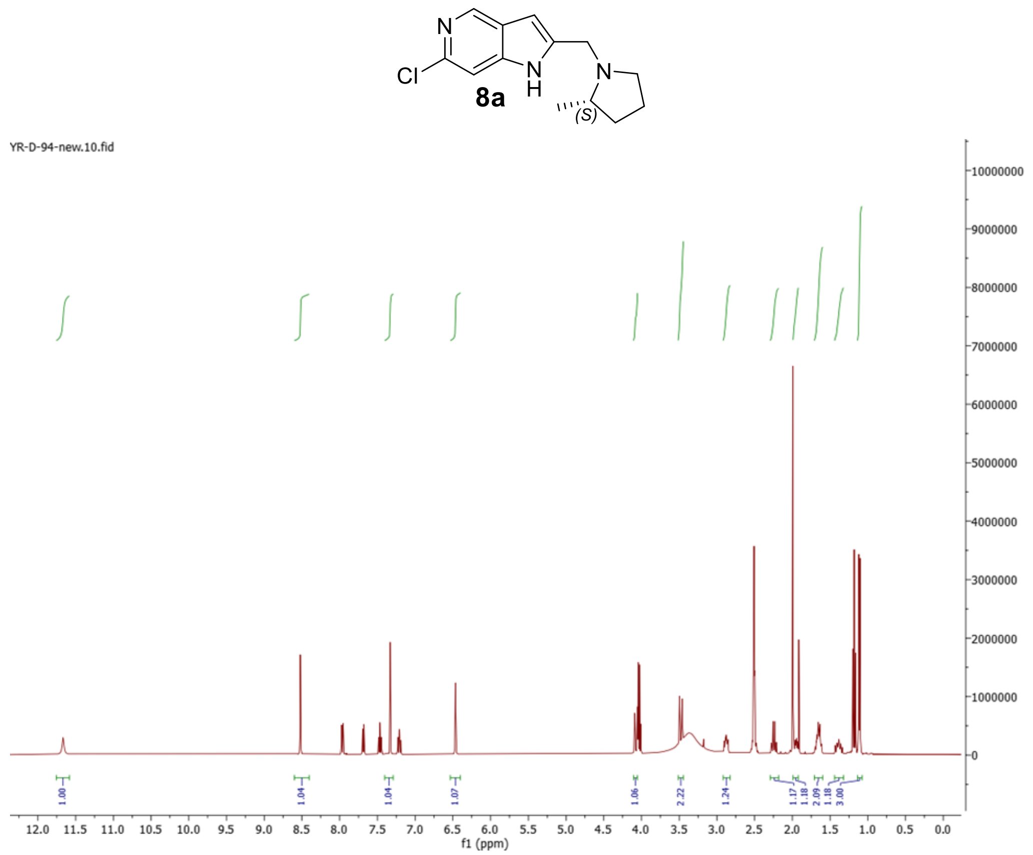

Figure S17. ^{1}H NMR of **8a** in DMSO-d_6.

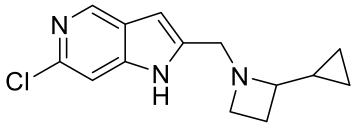

8b

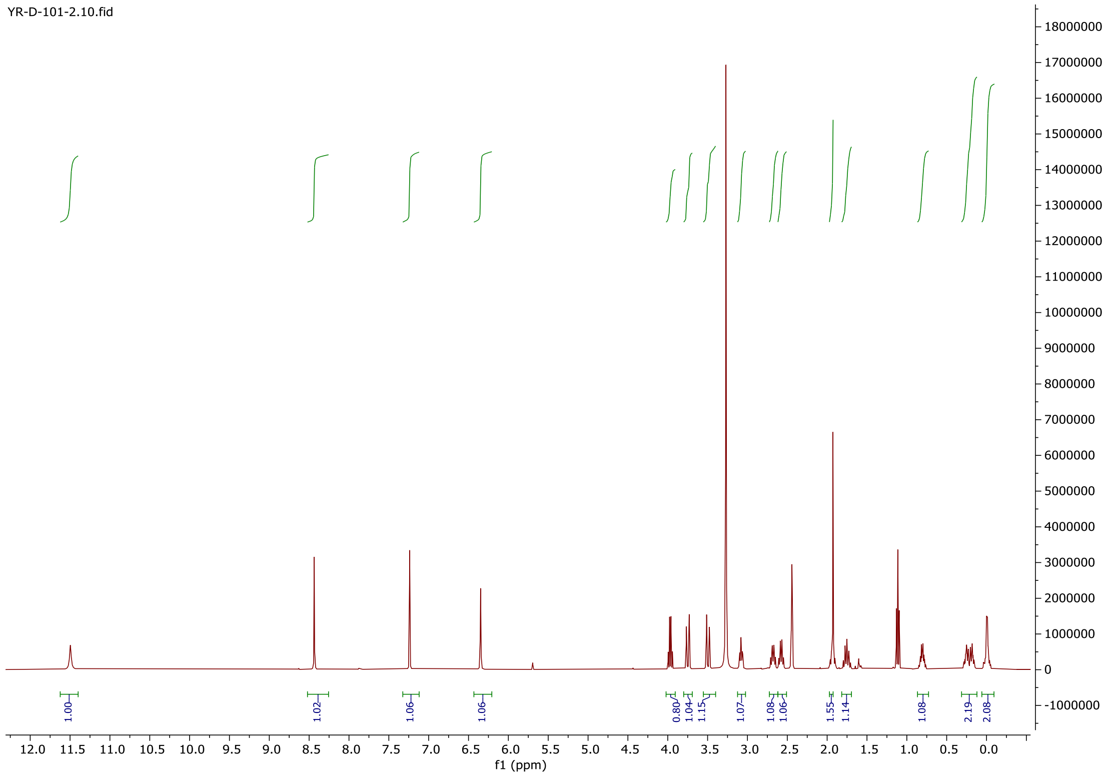

Figure S18. ^{1}H NMR of **8b** in DMSO-d$_6$.

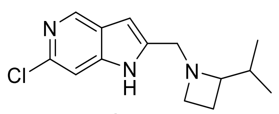

8c

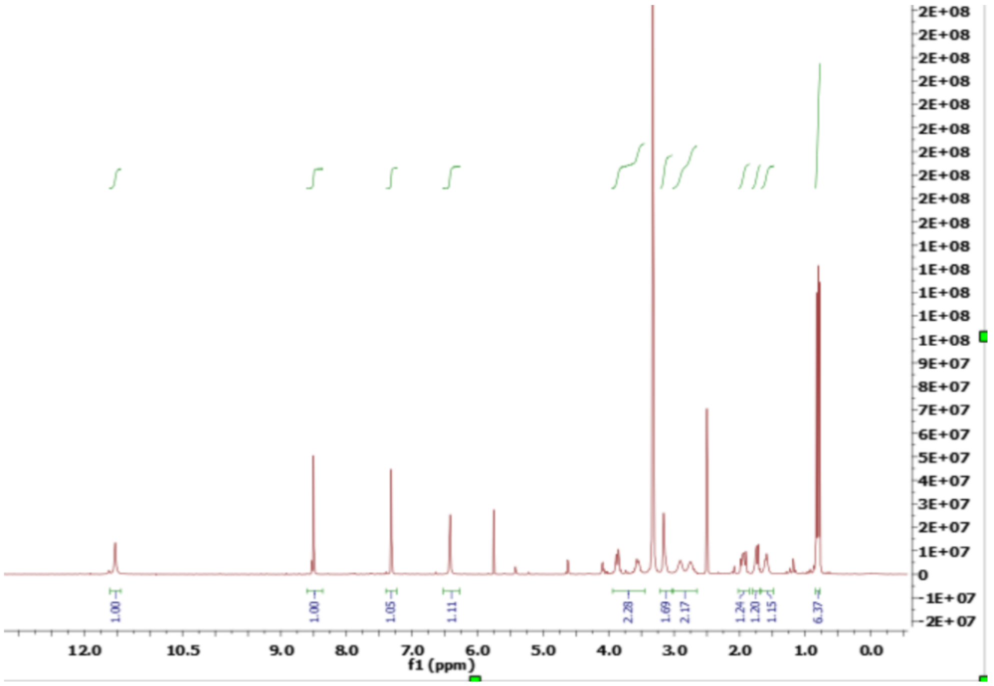

Figure S19. ^1H NMR of **8c** in DMSO-d_6.

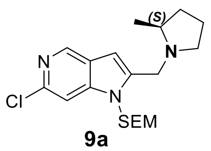

9a

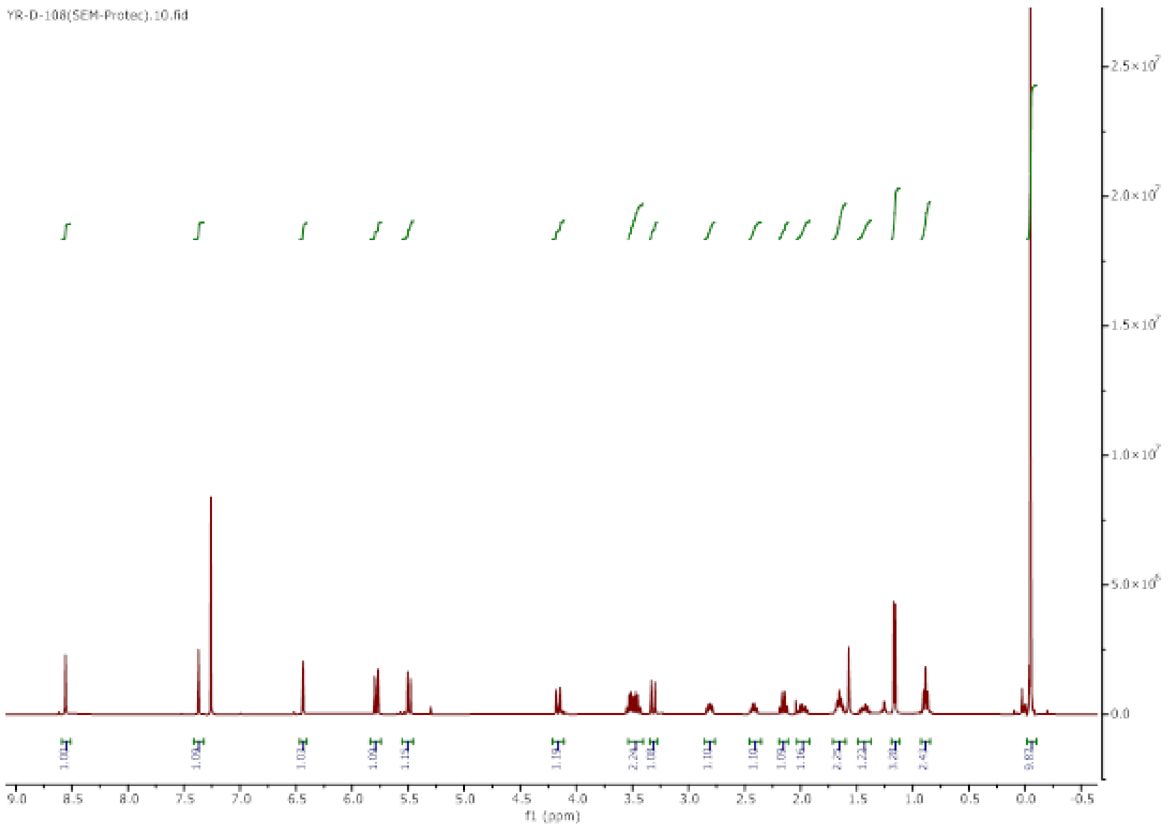

Figure S20. ^{1}H NMR of **9a** in $CDCl_3$

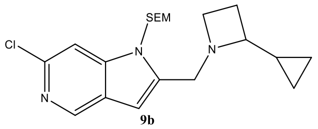

9b

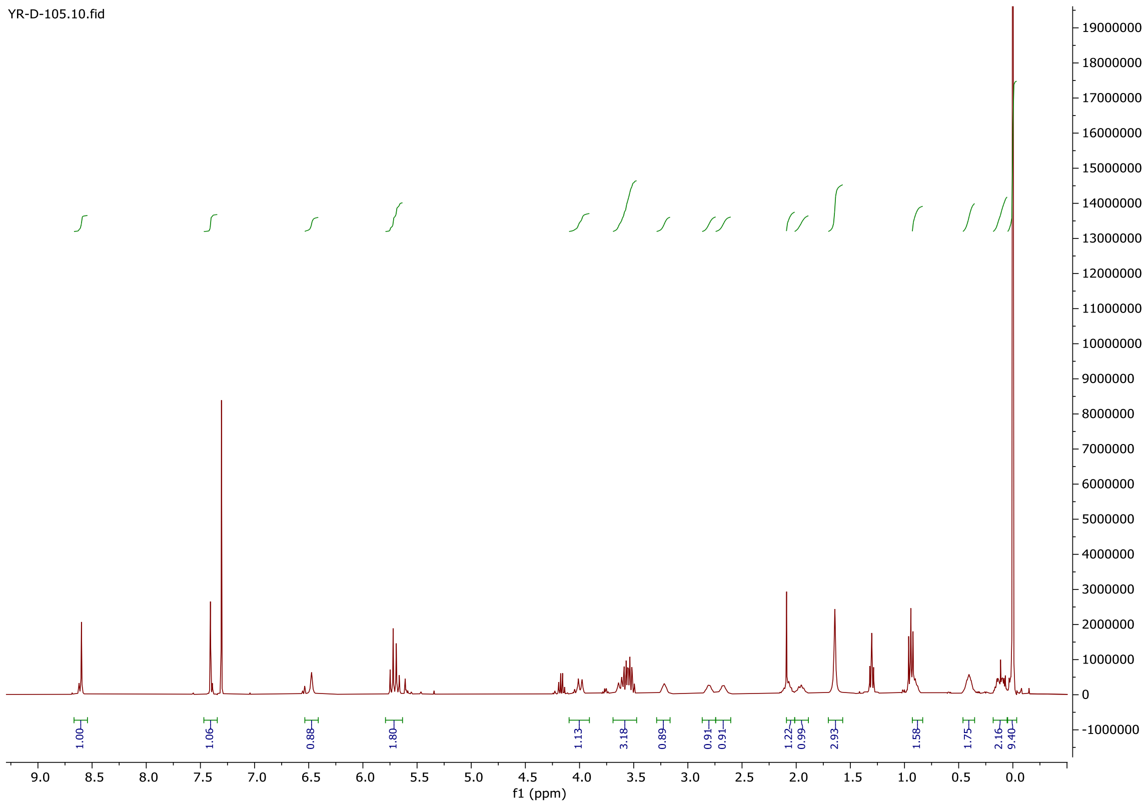

Figure S21. ^{1}H NMR of **9b** in Chloroform-d.

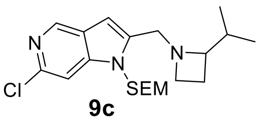

9c

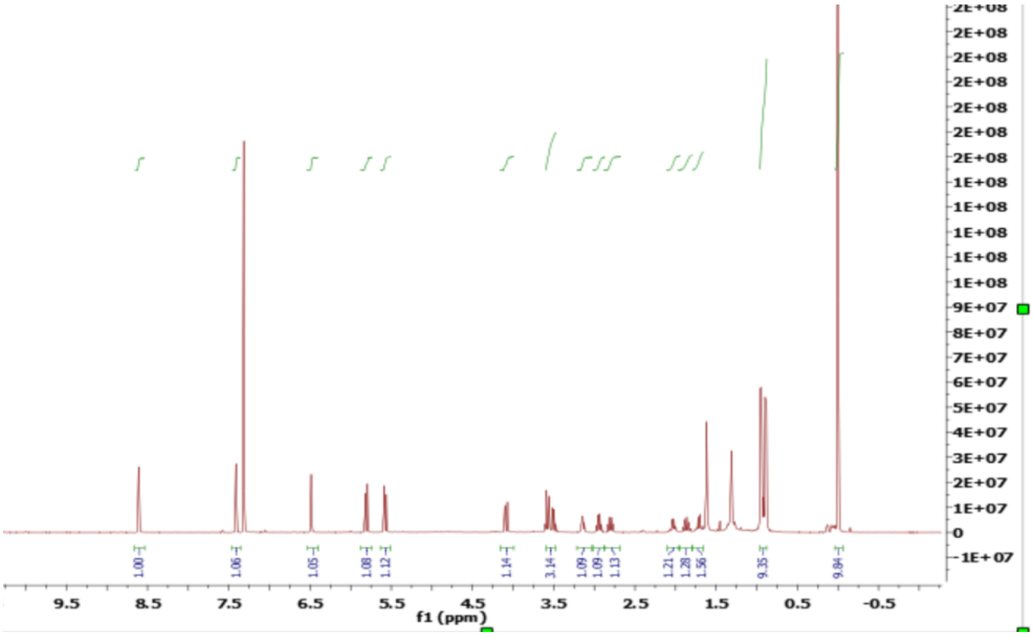

Figure S22. ^{1}H NMR of **9c** in Chloroform-d.

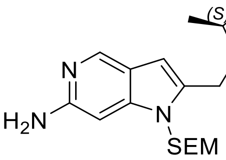

11a

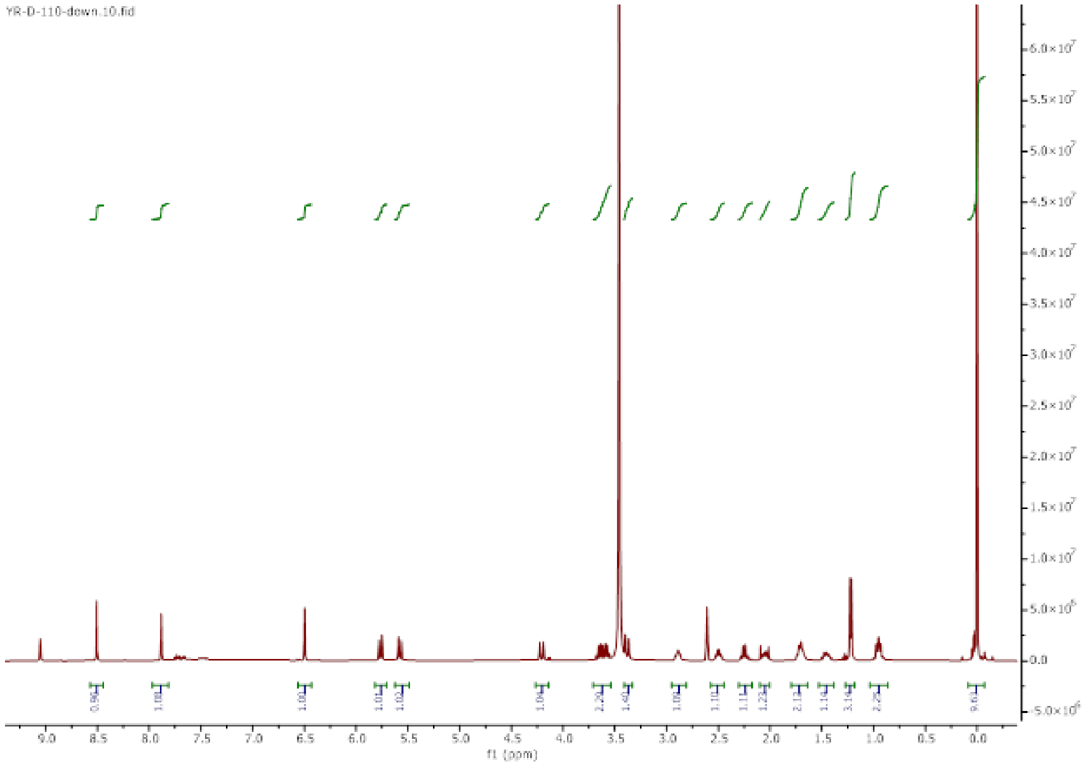

Figure S23. ^{1}H NMR of **11a** in Chloroform-d.

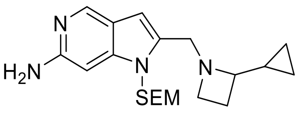

11b

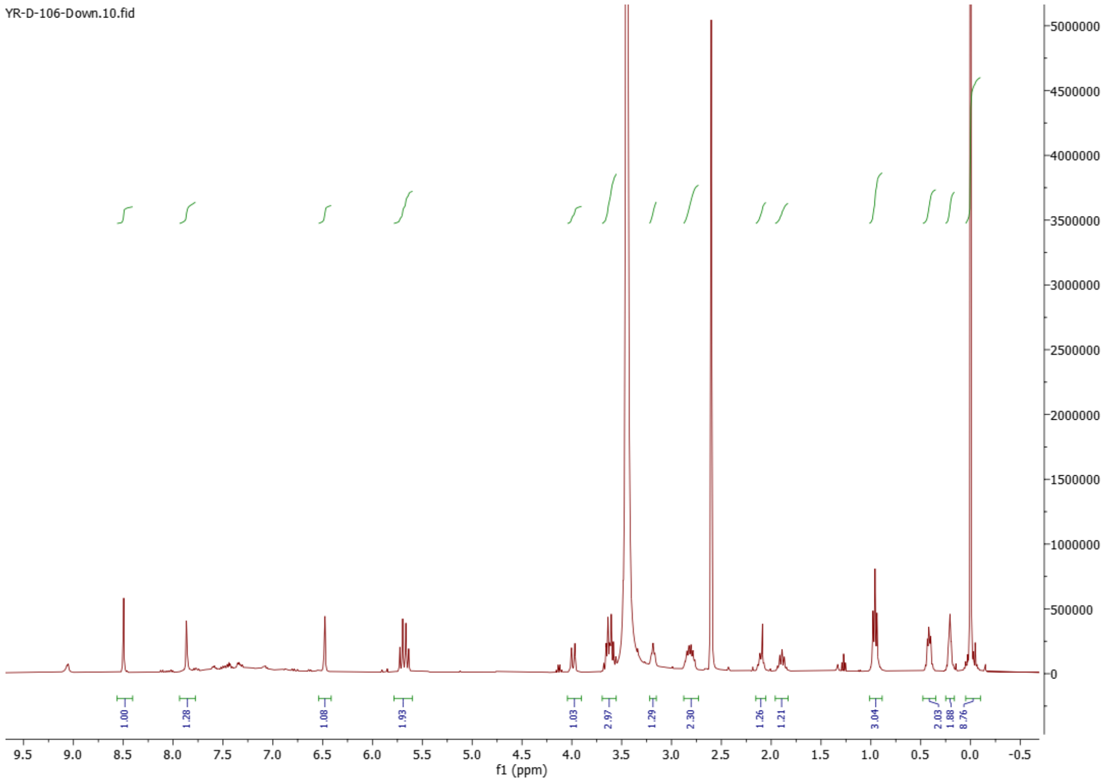

Figure S24. ^{1}H NMR of **11b** in DMSO-d6.

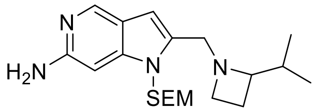

11c

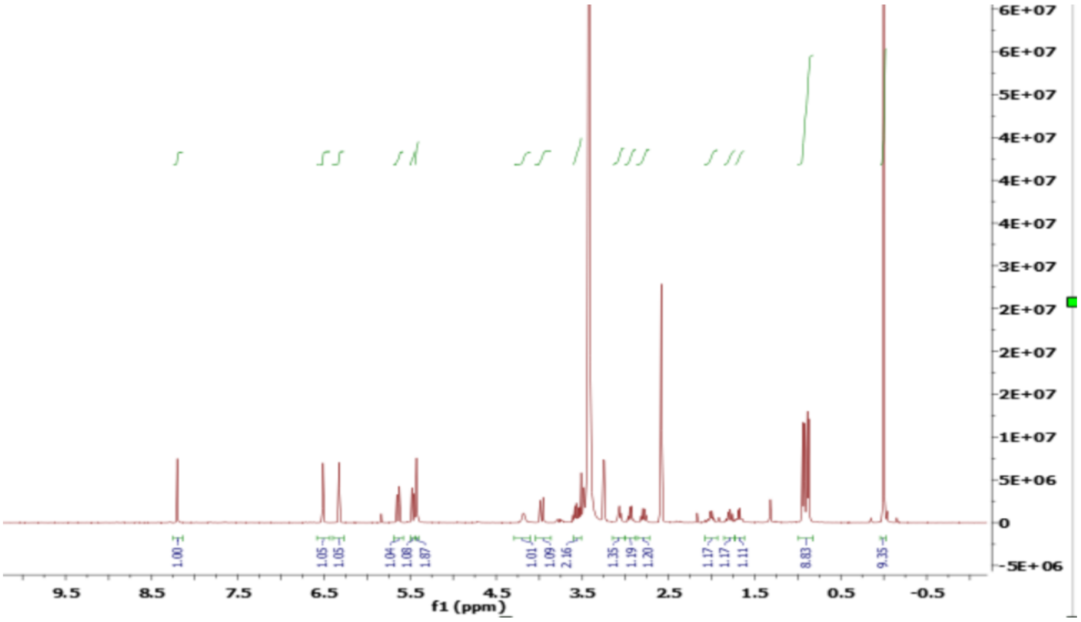

Figure S25. ^{1}H NMR of **11c** in DMSO-d_6.

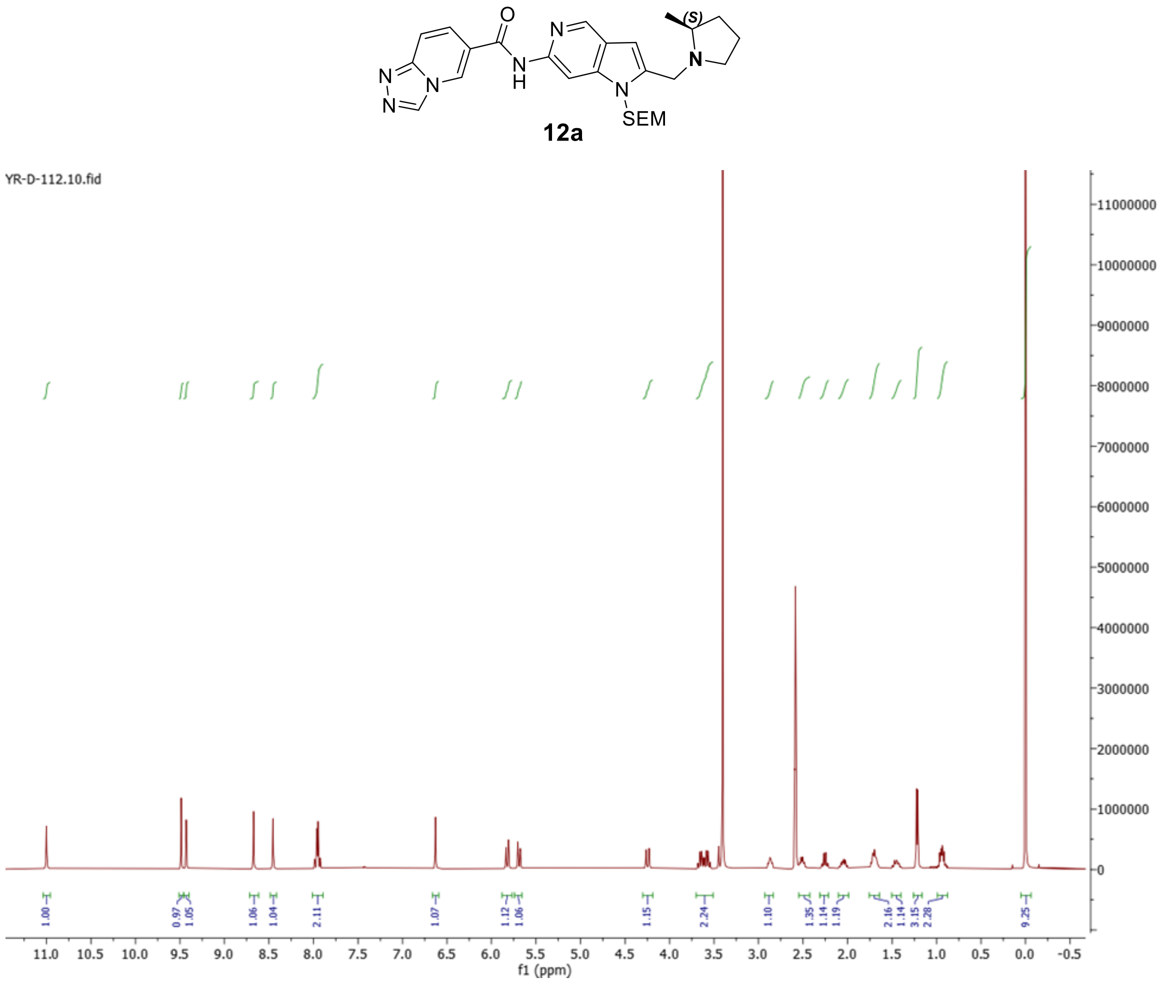

Figure S26. ^{1}H NMR of **12a** in DMSO-d$_6$.

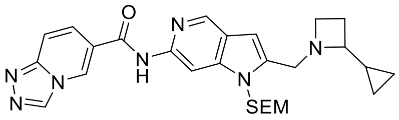

12b

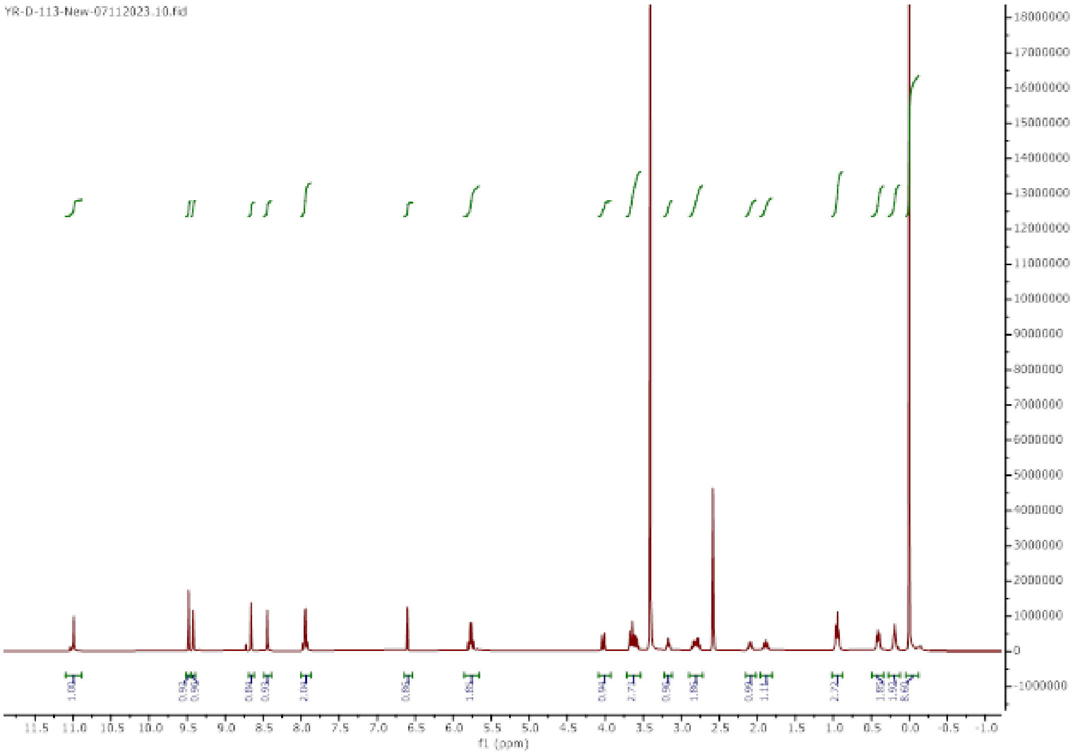

Figure S27. ${}^{1}H$ NMR of **12b** in DMSO-d_6.

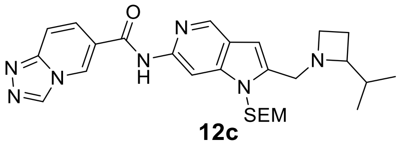

12c

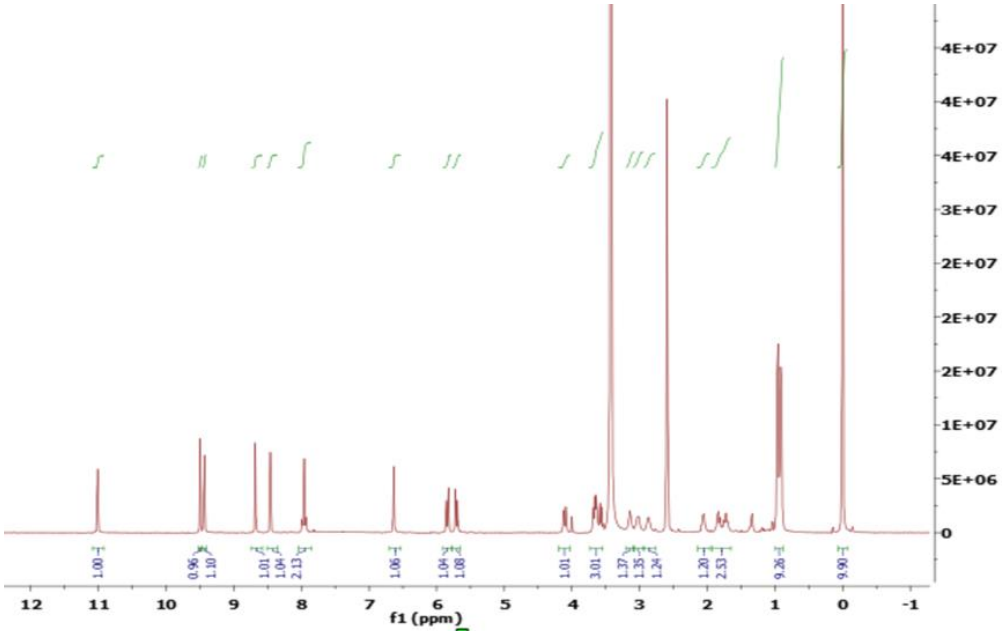

Figure S28. ^{1}H NMR of **12c** in DMSO-d6.

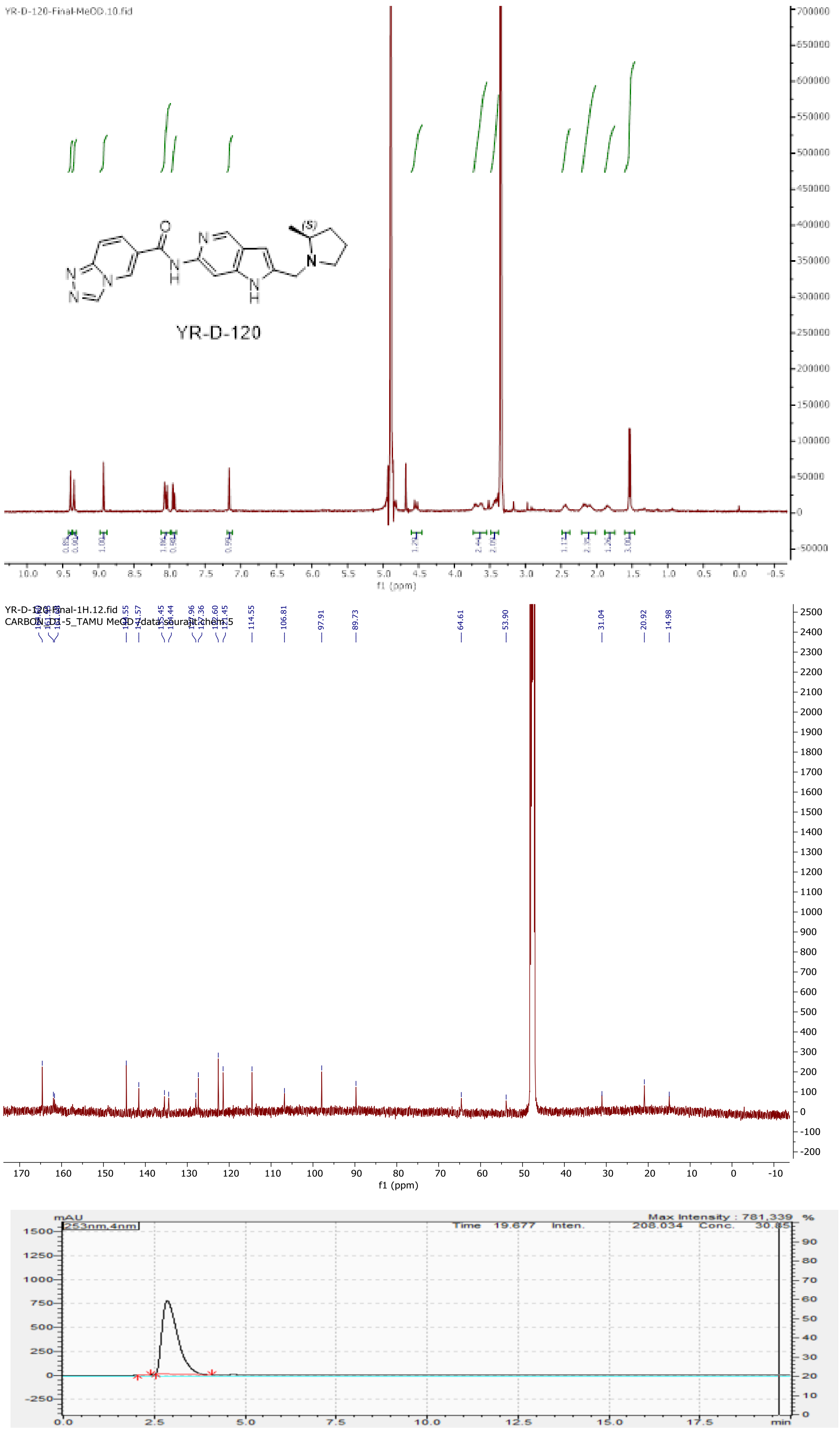

Figure S29. ^{1}H NMR and ^{13}C NMR of **YR-D-120** in CD_3OD and HPLC-profile of **YR-D-120** (Purity = 99.96%) analyzed by reverse-HPLC.

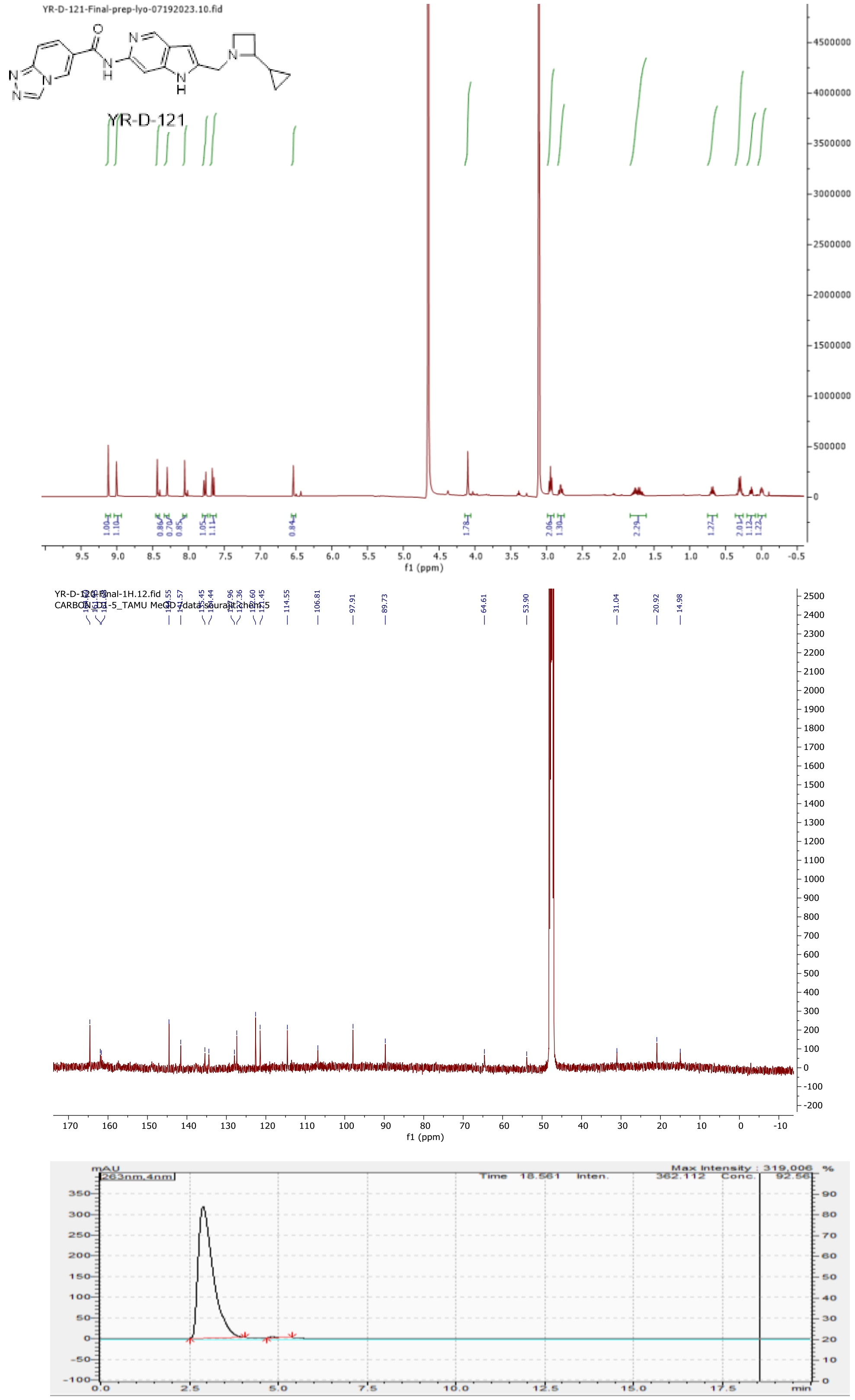

Figure S30. ^{1}H NMR and ^{13}C NMR of **YR-D-121** in CD_3OD and HPLC-profile of **YR-D-121** (Purity = 99.41%) analyzed by reverse-HPLC.

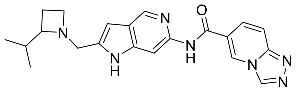

SR-C-107

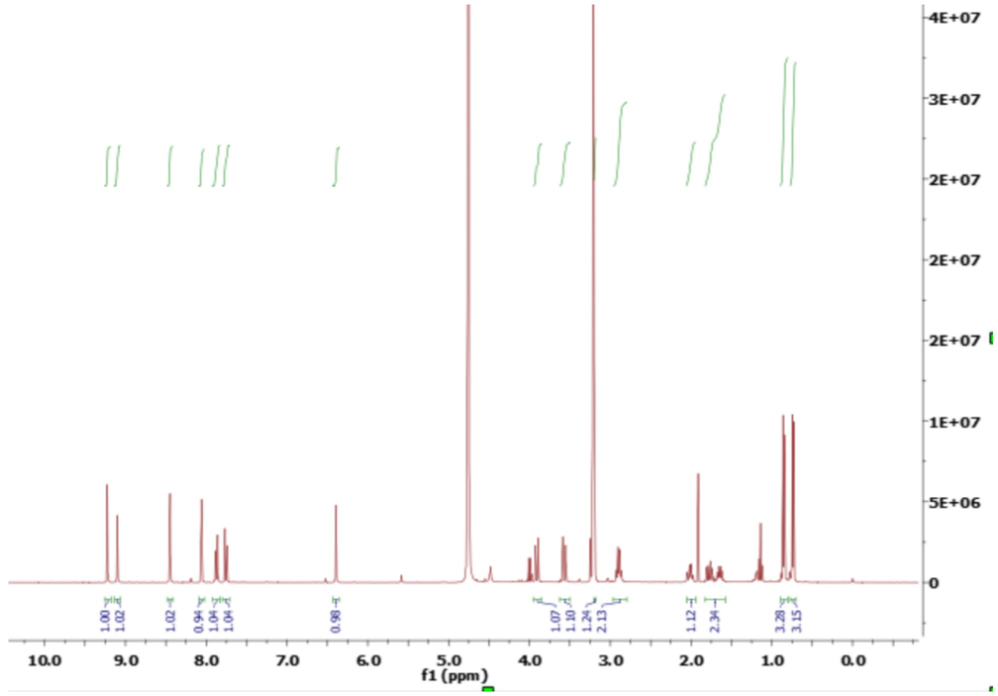

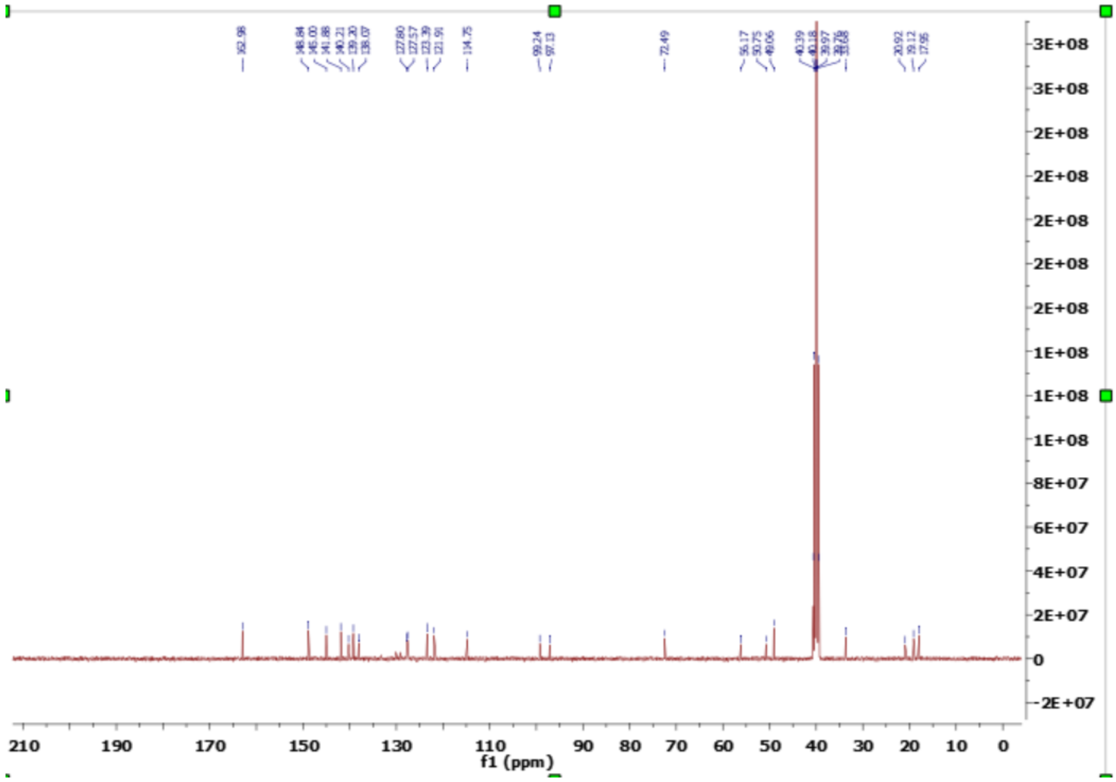

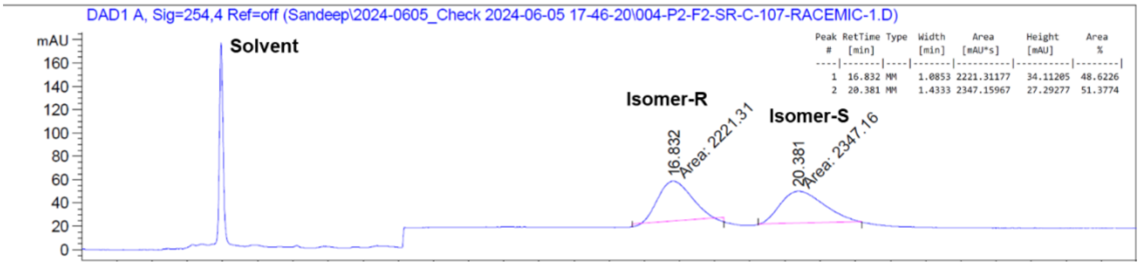

Figure S31. ^{1}H NMR of **SR-C-107** racemate in CD_3OD. ^{13}C NMR of **SR-C-107** in DMSO-d_6 and chiral HPLC profile.

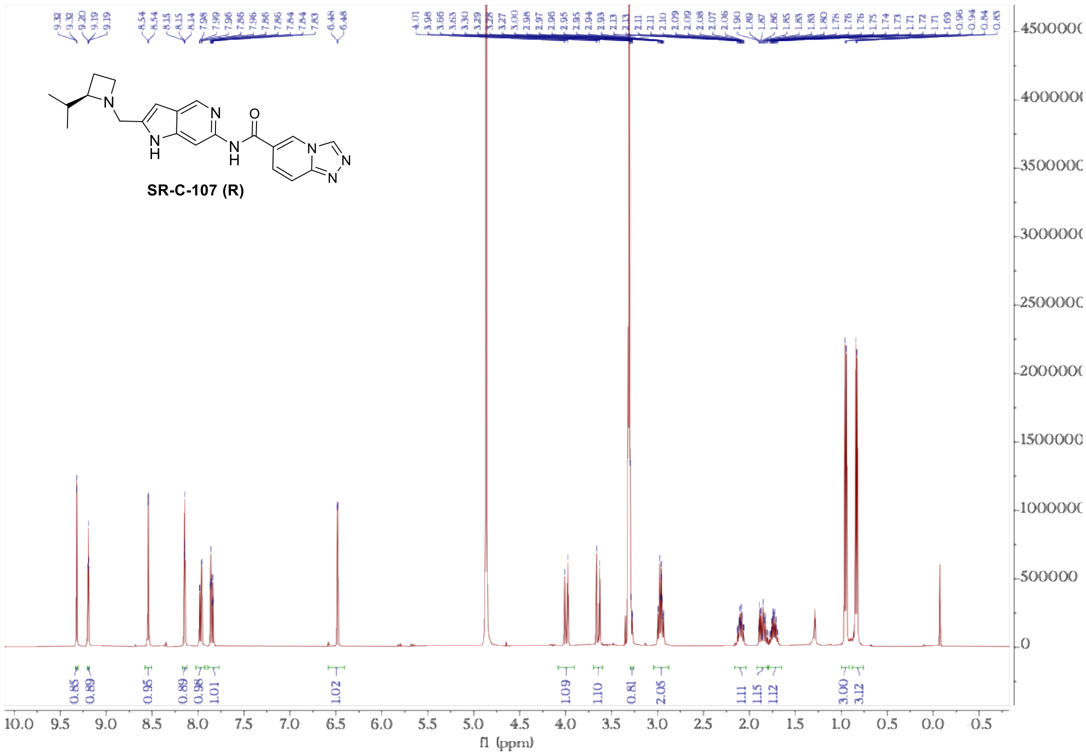
SR-C-107 (R)
f1 (ppm)

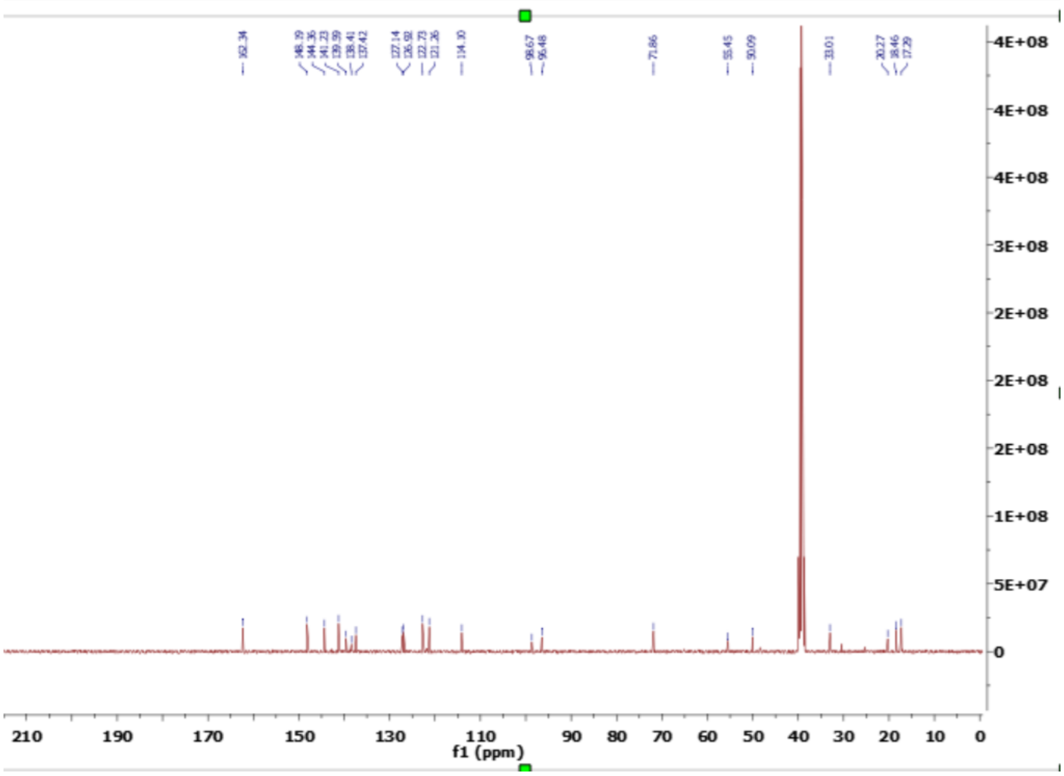
f1 (ppm)

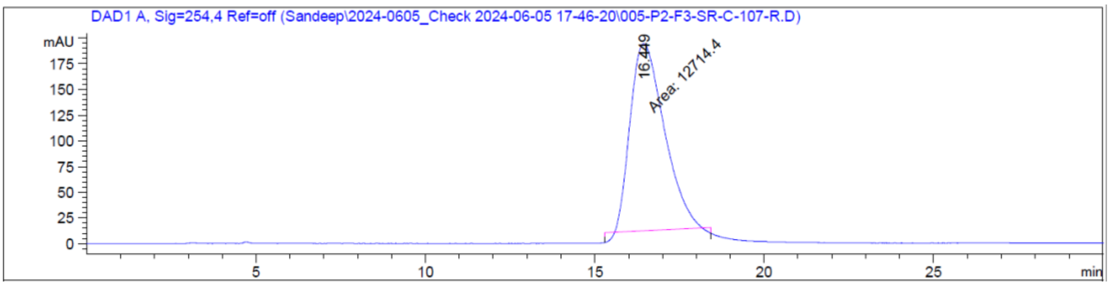

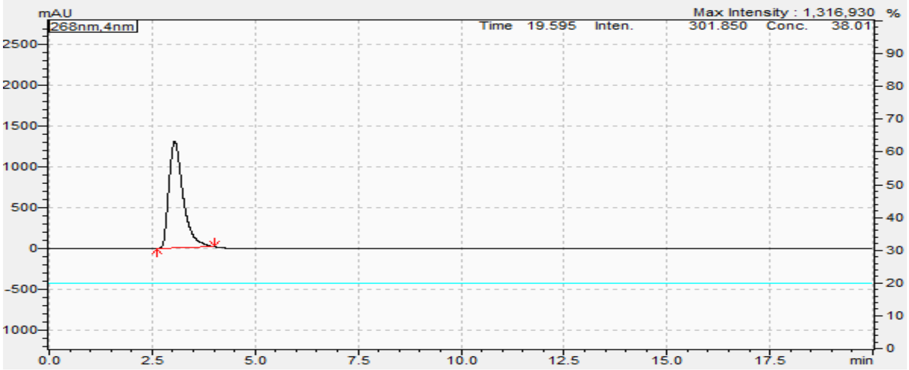

Figure S32. ^{1}H NMR of **SR-C-107 (R)** in CD_3OD. ^{13}C NMR of **SR-C-107 (R)** in DMSO-d_6. Chiral HPLC profile (top) and reverse phase HPLC profile (bottom, purity > 98%) of **SR-C-107 (R)**.

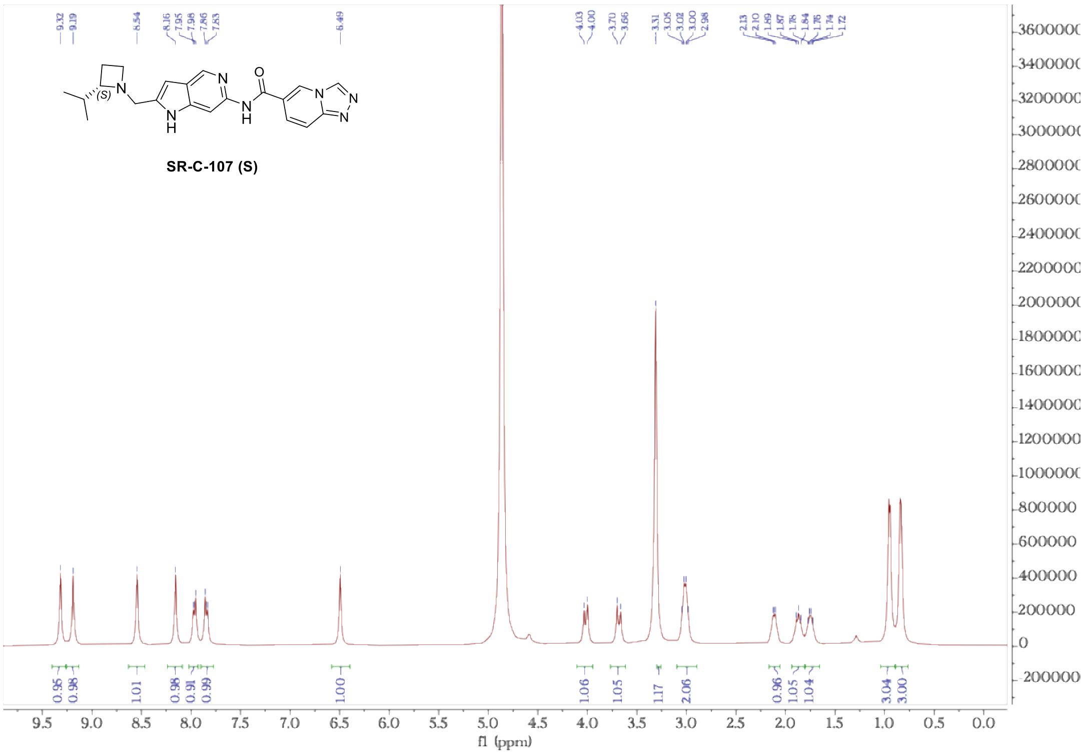
SR-C-107 (S)
f1 (ppm)

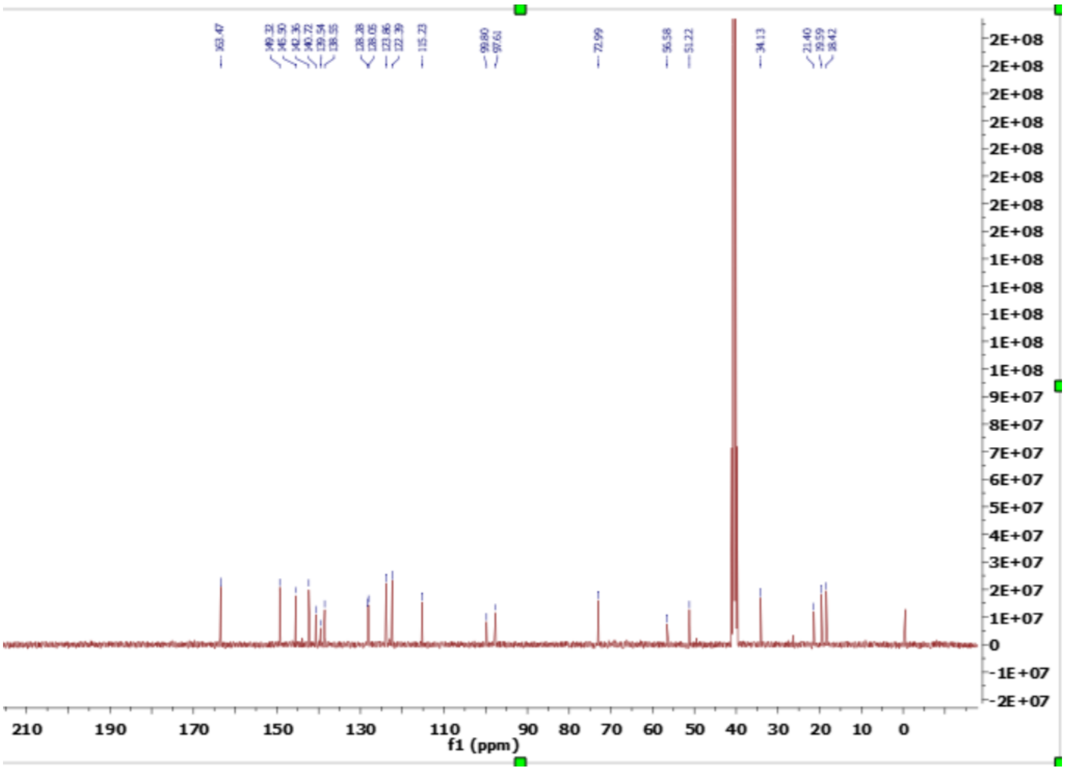
f1 (ppm)

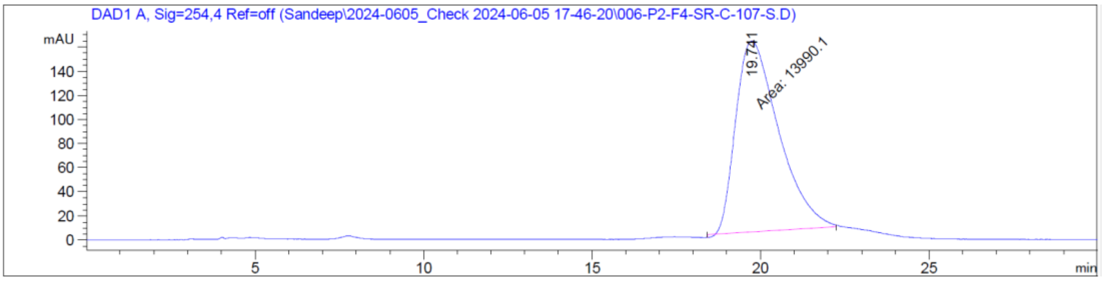

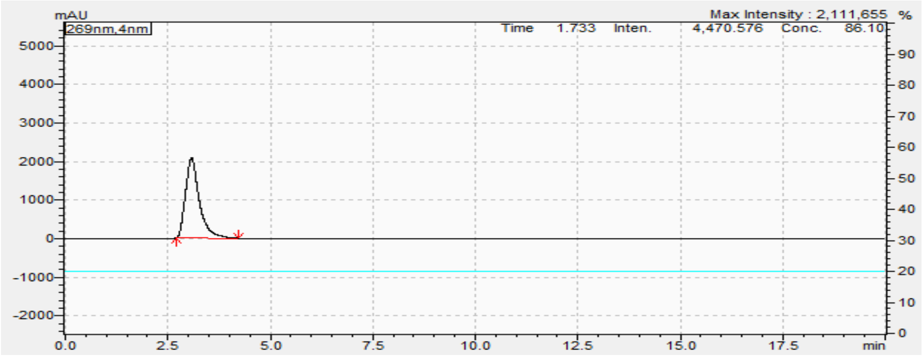

Figure S33. ^{1}H NMR of **SR-C-107 (S)** in CD_3OD. ^{13}C NMR of **SR-C-107 (S)** in DMSO-d6. Chiral HPLC profile (top) and reverse phase HPLC profile (bottom, purity)

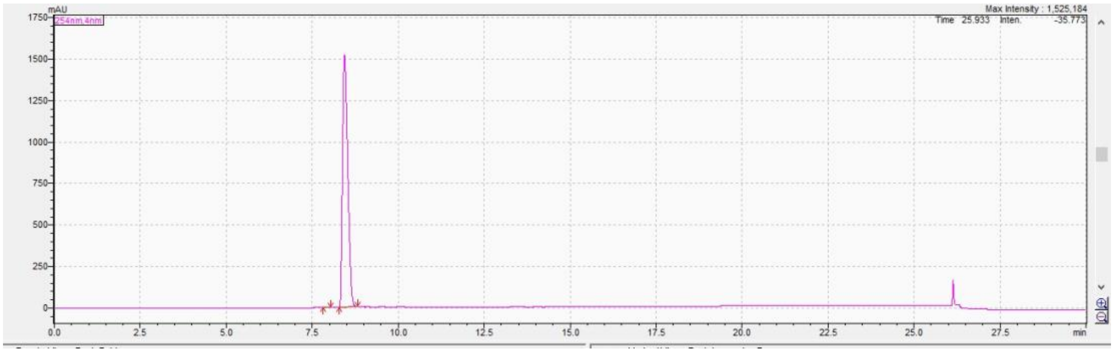

Figure S34. HPLC-profile of **13** (Purity = 99.89%) analyzed by reverse-HPLC.

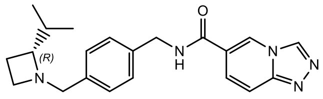

O
N
H
N
N
N
N
(R)

13 (R)

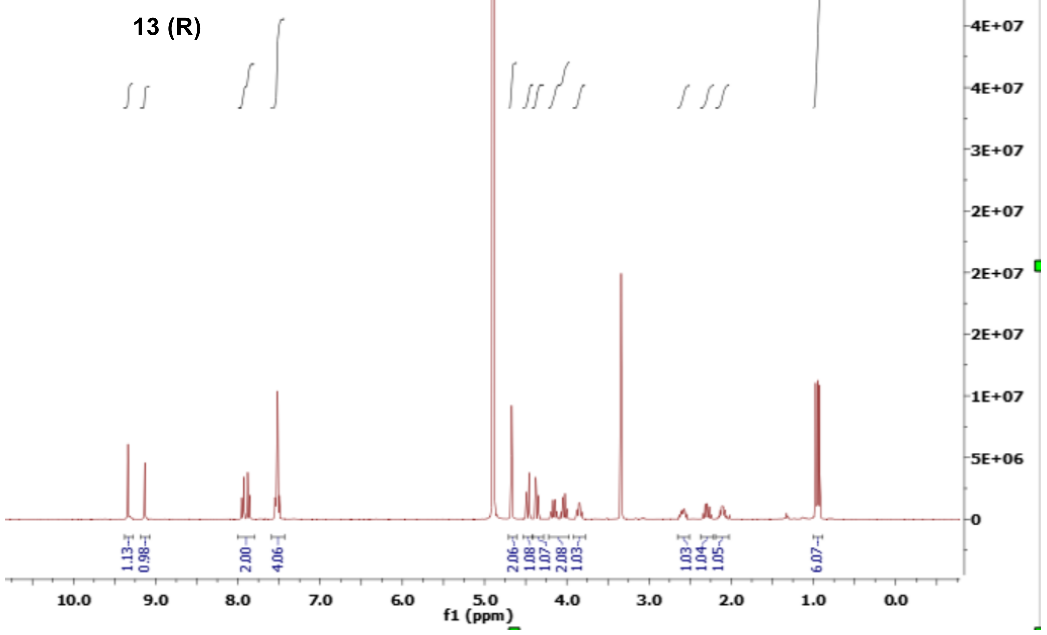

1.13
0.98
2.00
4.06
2.06
1.08
1.07
1.07
2.08
1.03
1.03
1.04
1.05
6.07
f1 (ppm)

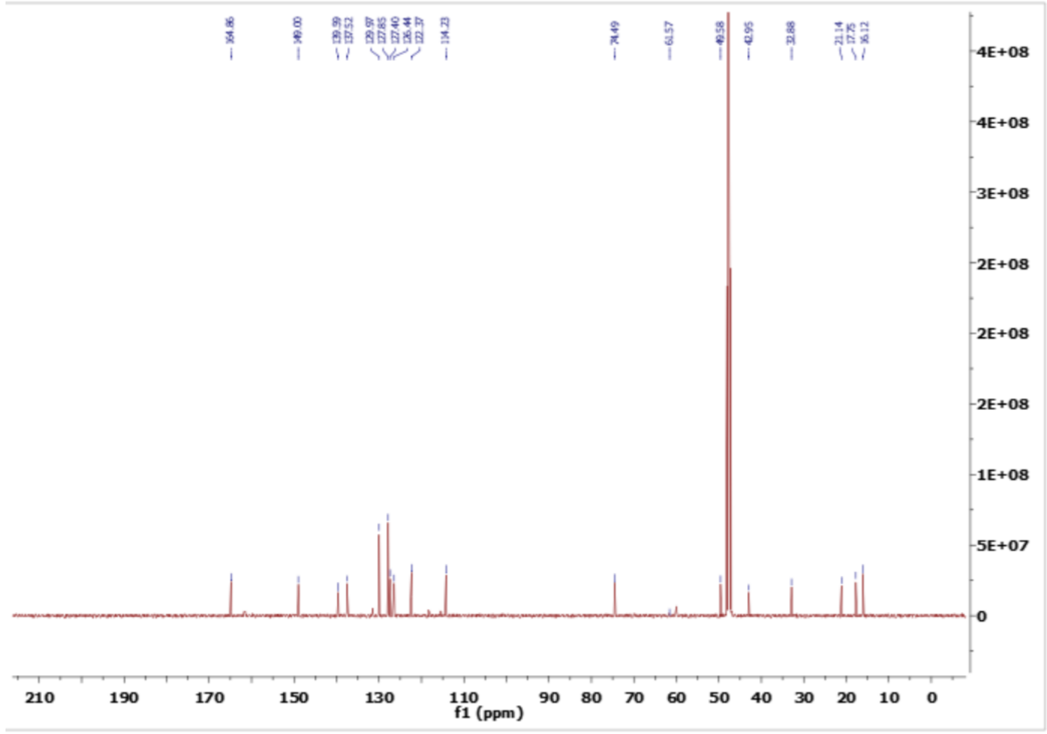

164.86
149.00
139.59
137.52
129.97
127.85
127.40
126.44
122.37
114.23
74.49
61.57
49.58
42.95
32.88
21.14
17.75
16.12
f1 (ppm)

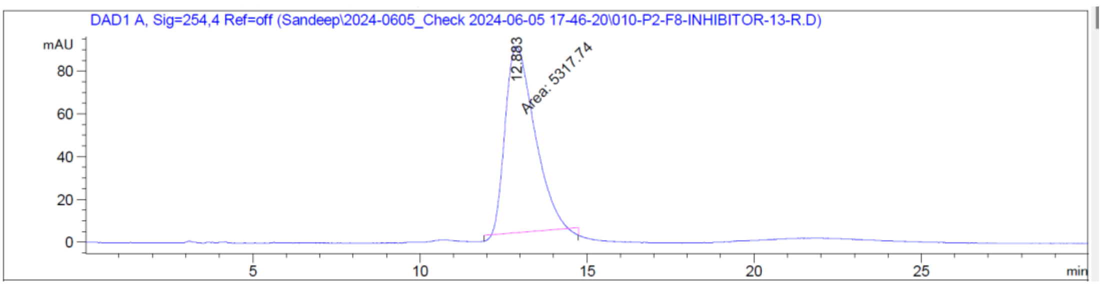

DAD1 A, Sig=254,4 Ref=off (Sandeep\2024-0605_Check 2024-06-05 17-46-20\010-P2-F8-INHIBITOR-13-R.D)
mAU
12.883
Area: 5317.74
min

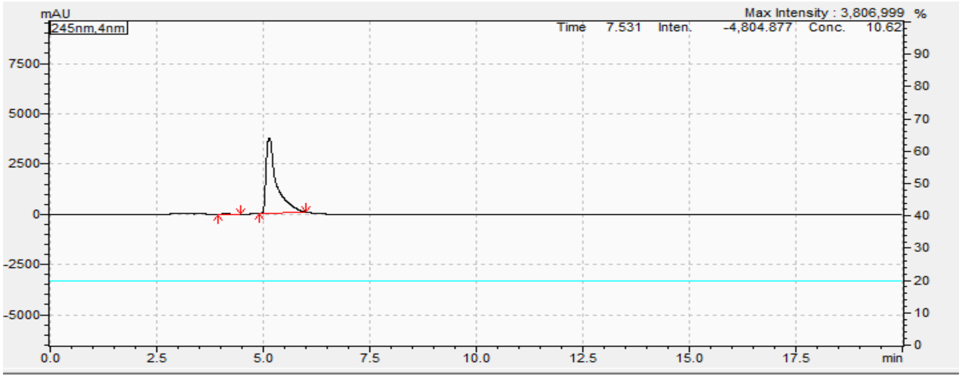

Figure S35. ^{1}H NMR and ^{13}C NMR of **Inhibitor-13-R-Isomer** in CD_3OD. Chiral HPLC-profile reverse phase HPLC profile (bottom, purity = 99.4 %) of **Inhibitor-13-R-Isomer.**

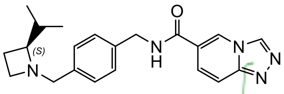

13 (S)

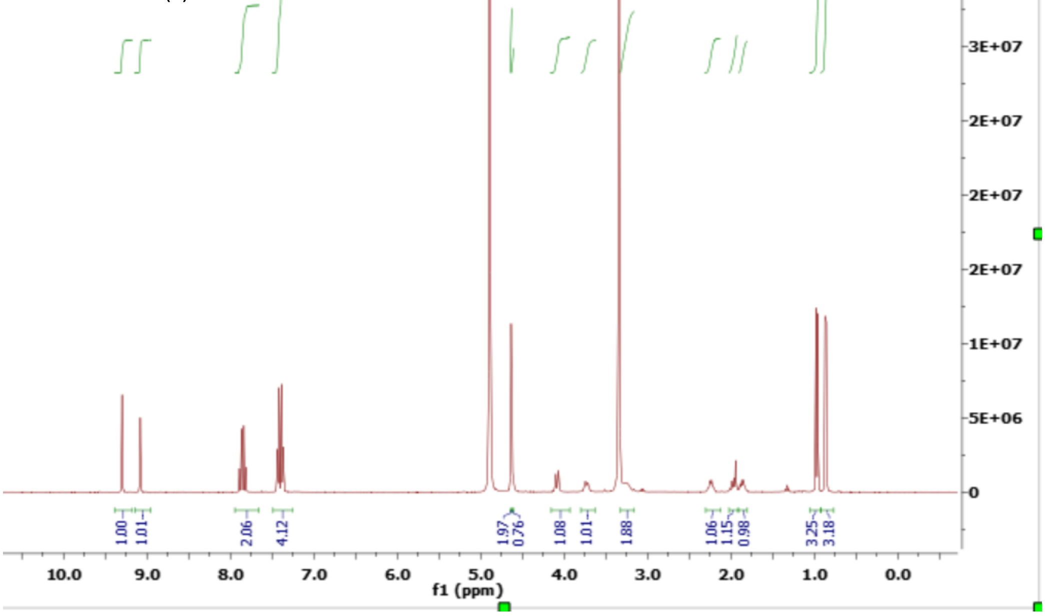

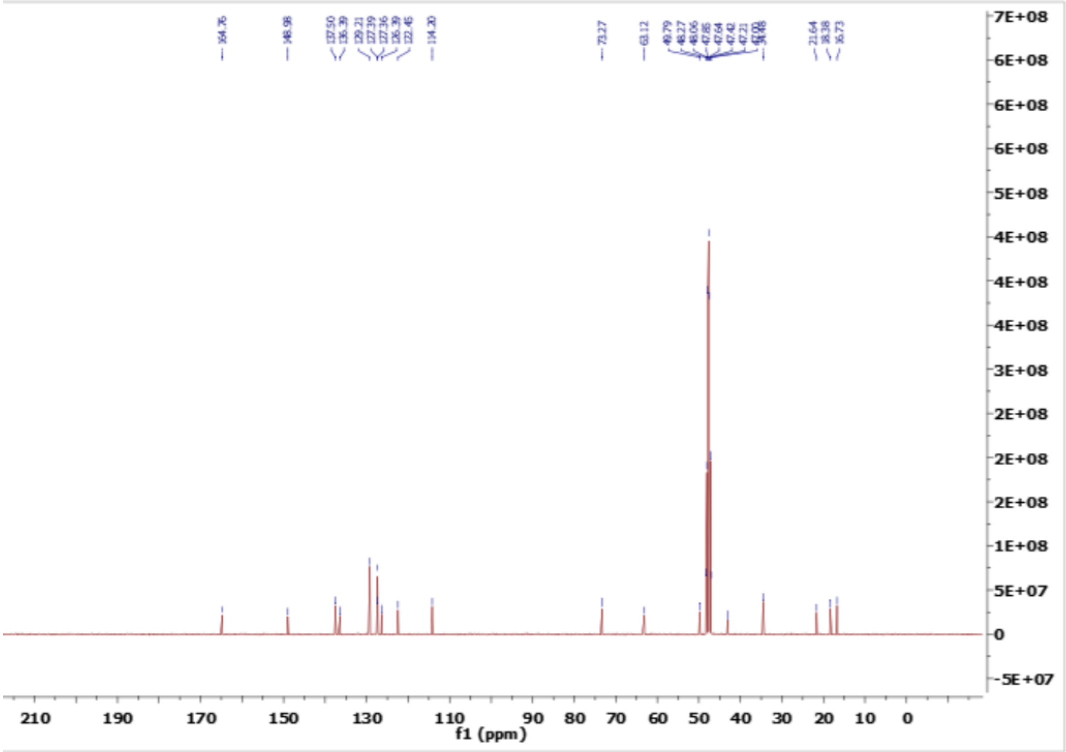

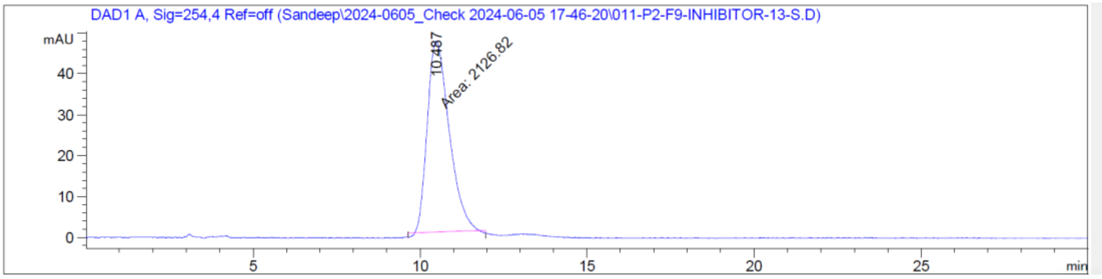
DAD1 A, Sig=254,4 Ref=off (Sandeep\2024-0605_Check 2024-06-05 17-46-20\011-P2-F9-INHIBITOR-13-S.D)
10.487
Area: 2126.82

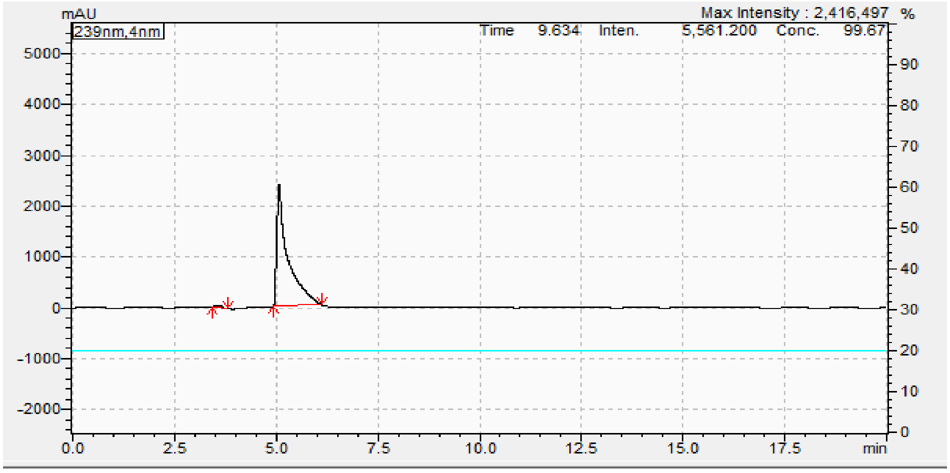

Figure S36. ^{1}H NMR and ^{13}C NMR of **Inhibitor-13-S-Isomer** in CD_3OD. Chiral HPLC-profile reverse phase HPLC profile (bottom, purity = 98.7 %) of **Inhibitor-13-S-Isomer.**

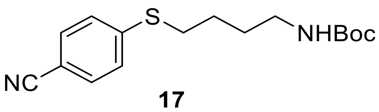

17

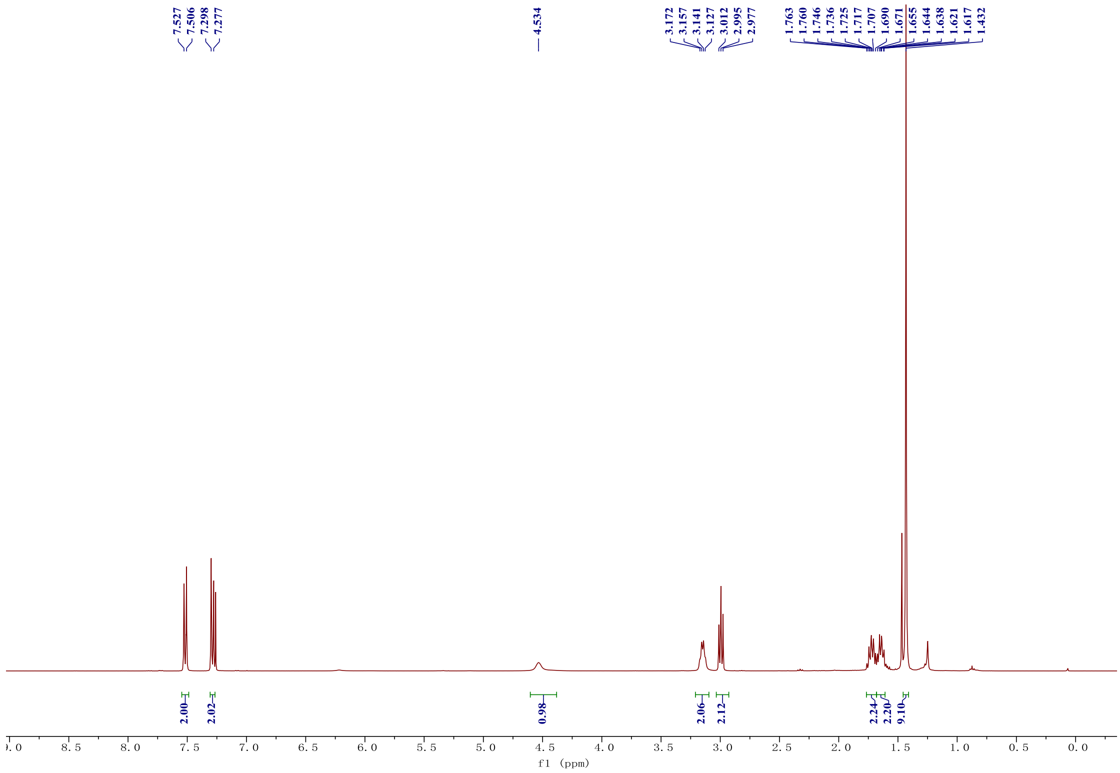

Figure S37. ^{1}H NMR of **17** in Chloroform-d.

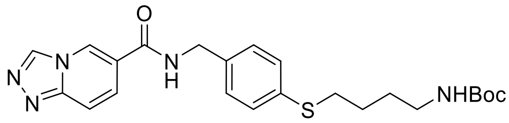

18

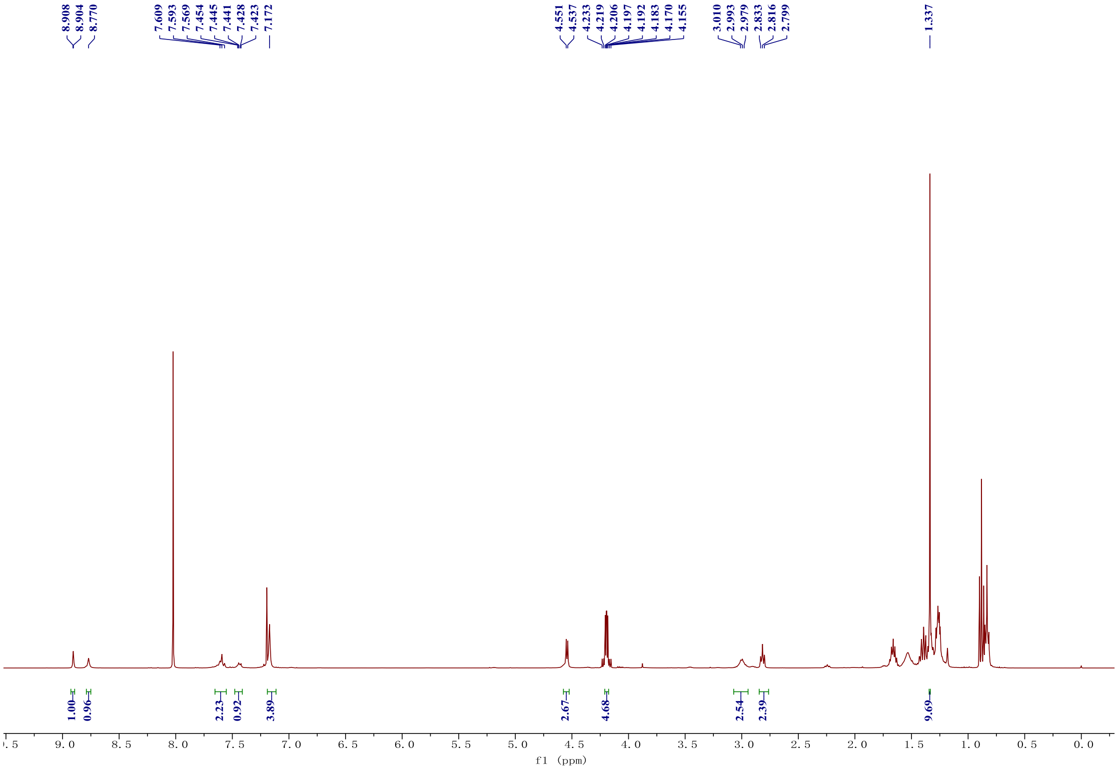

Figure S38. ^{1}H NMR of **18** in Chloroform-d.

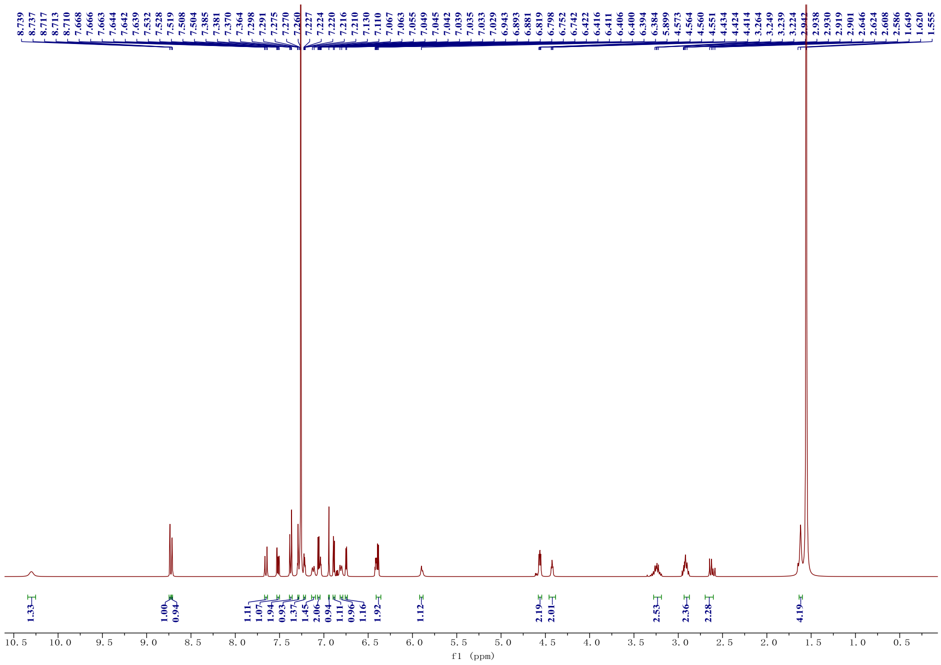

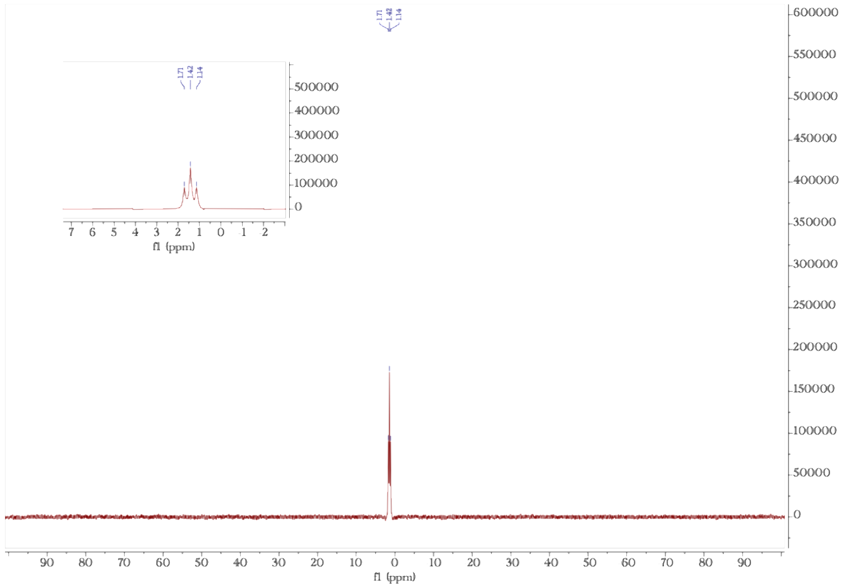

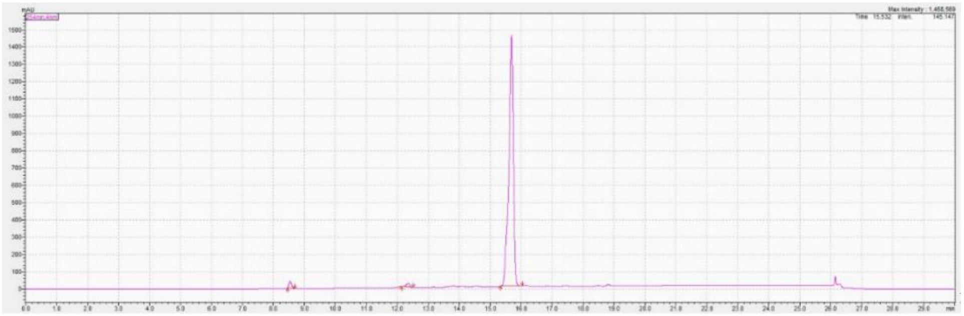

Figure S39. ^{1}H and ^{11}B NMR of **Tracer 1** in Chloroform-d and HPLC-profile of **Tracer 1** (Purity = 97.38%) analyzed by reverse-HPLC.

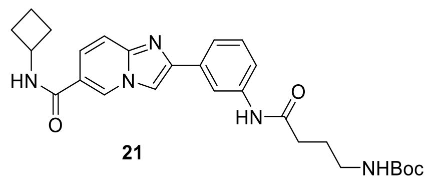

21

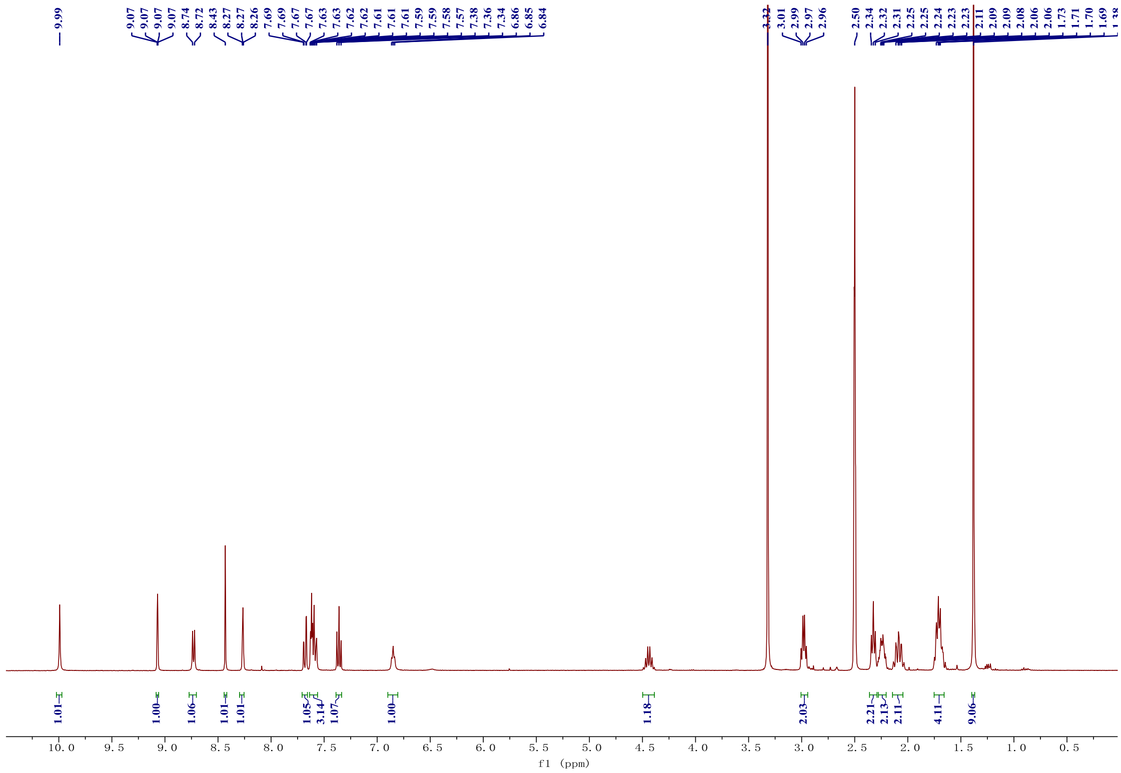

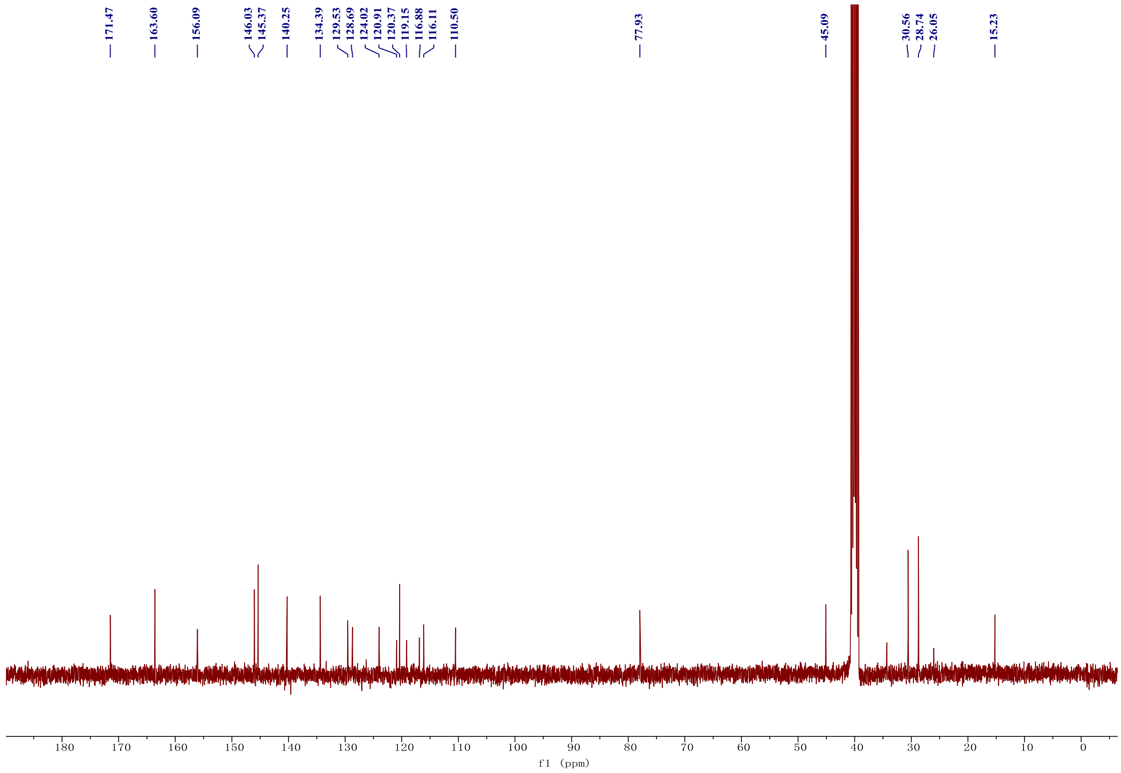

Figure S40. ^{1}H and ^{13}C NMR of compound **21** in DMSO-d$_6$.

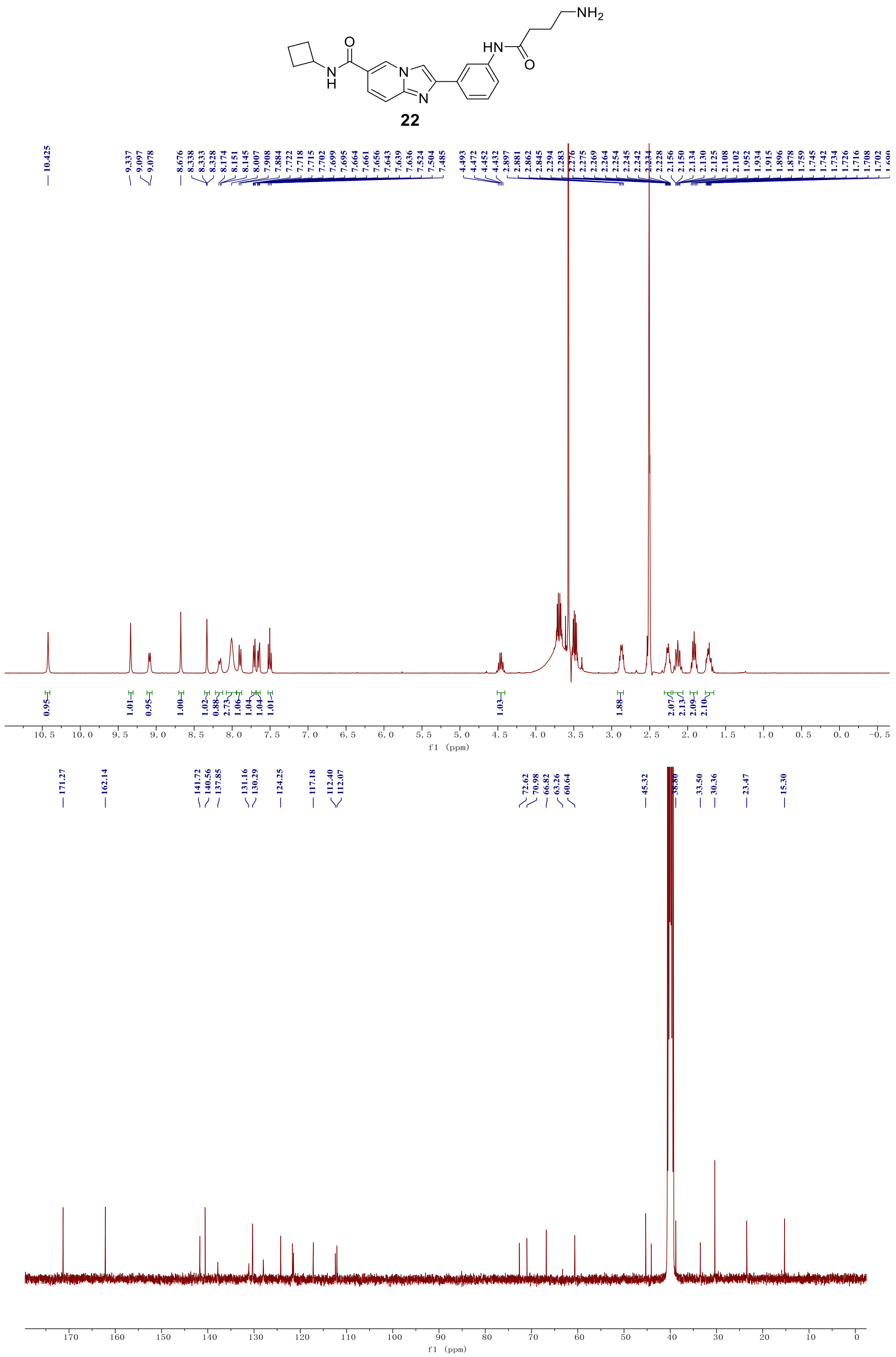

Figure S41. ^{1}H and ^{13}C NMR of compound **22** in DMSO-d6.

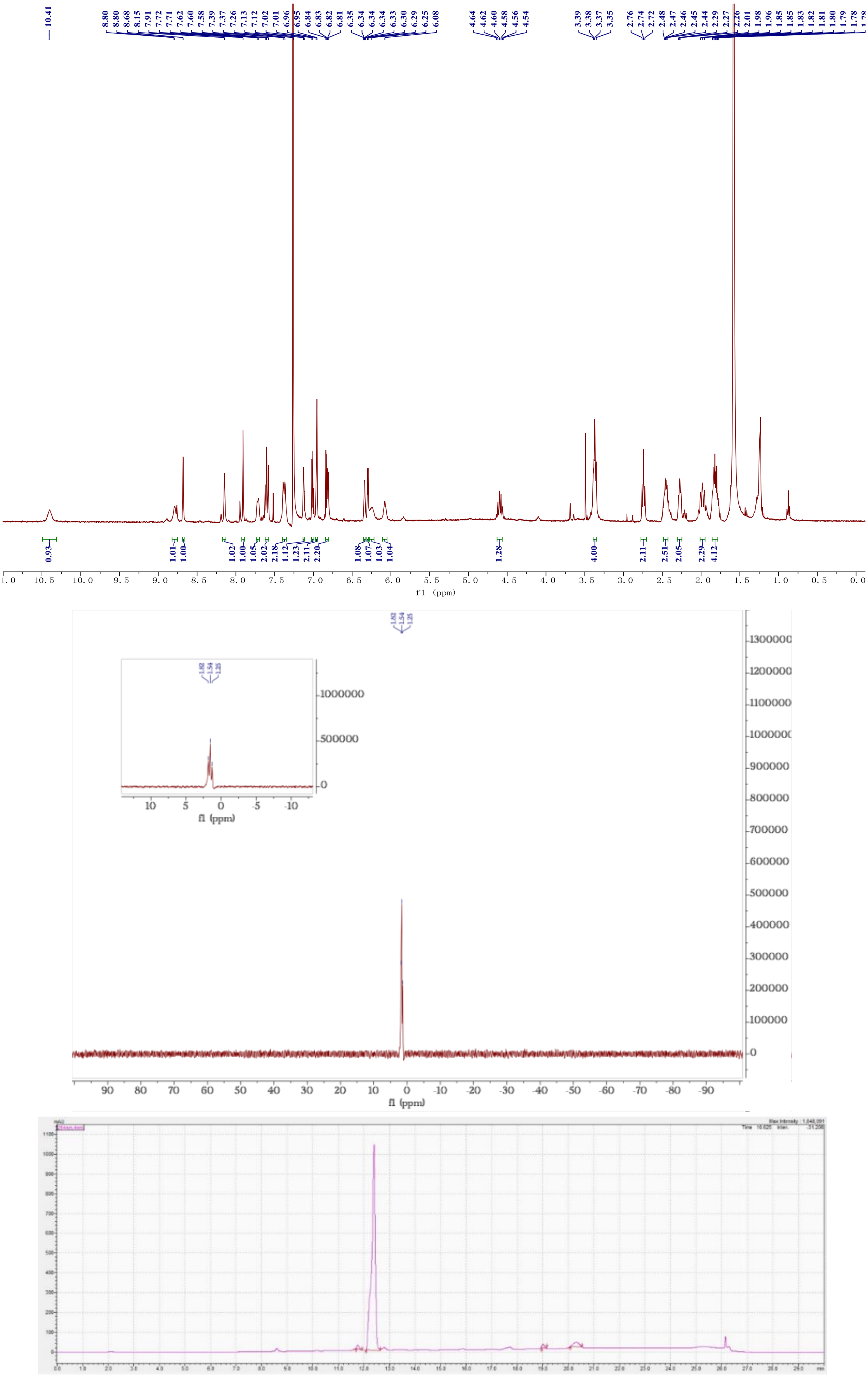

Figure S42. ^{1}H and ^{11}B of NMR of **Tracer 2** in Chloroform-d and HPLC-profile of **Tracer 2** (Purity = 95.03%) analyzed by reverse-HPLC.

Formula	$C_{13}H_{17}NO_2$	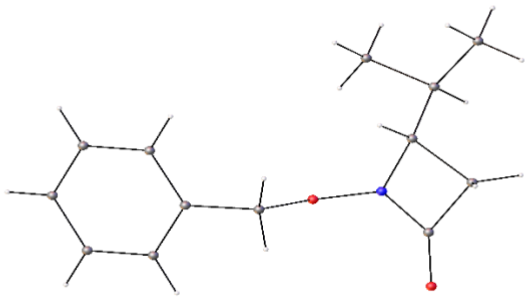
$D_{calc.}$/ g cm^{-3}	1.192	
m/mm^{-1}	0.642	
Formula Weight	219.27	
Colour	colourless	
Shape	plate-shaped	
Size/mm^3	0.12×0.07×0.03	
T/K	100.00(10)	
Crystal System	orthorhombic	
Flack Parameter	-0.04(7)	
Hooft Parameter	-0.05(4)	
Space Group	$P2_12_12_1$	
a/Å	5.57030(10)	
b/Å	9.88460(10)	
c/Å	22.1842(2)	
a/°	90	
b/°	90	
g/°	90	
V/Å^3	1221.47(3)	
Z	4	
Z'	1	
Wavelength/Å	1.54184	
Radiation type	Cu K*a*	
Q_{min}/°	3.985	
Q_{max}/°	79.611	
Measured Refl's.	24286	
Indep't Refl's	2633	
Refl's I≥2 *s*(I)	2588	
R_{int}	0.0390	
Parameters	147	
Restraints	0	
Largest Peak	0.119	
Deepest Hole	-0.132	
GooF	1.080	
wR_2 (all data)	0.0733	
wR_2	0.0730	
R_1 (all data)	0.0279	
R_1	0.0275	

Figure S43. Crystal Data of compound **5a**.

Formula	$C_{13}H_{17}NO_2$
$D_{calc.}$/ g cm^{-3}	1.192
m/mm^{-1}	0.642
Formula Weight	219.27
Colour	colourless
Shape	plate-shaped
Size/mm^3	0.12×0.12×0.02
T/K	100.00(10)
Crystal System	orthorhombic
Flack Parameter	0.01(5)
Hooft Parameter	0.03(5)
Space Group	$P2_12_12_1$
a/Å	5.57010(10)
b/Å	9.88810(10)
c/Å	22.1770(2)
a/°	90
b/°	90
g/°	90
V/Å^3	1221.46(3)
Z	4
Z'	1
Wavelength/Å	1.54184
Radiation type	Cu K$_a$
Q_{min}/°	3.987
Q_{max}/°	69.982
Measured Refl's.	12058
Indep't Refl's	2298
Refl's I≥2 *s*(I)	2262
R_{int}	0.0211
Parameters	148
Restraints	0
Largest Peak	0.146
Deepest Hole	-0.110
GooF	1.083
wR_2 (all data)	0.0598
wR_2	0.0593
R_1 (all data)	0.0233
R_1	0.0228

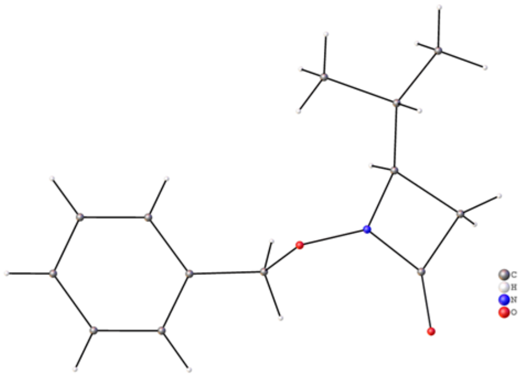

Figure S44. Crystal Data of compound **5b**.
