# Supplementary material for: Prioritization of Eleven-Nineteen-Leukemia Inhibitors as Orally Available Drug Candidates for Acute Myeloid Leukemia
Source: J Med Chem. 2024 Nov 12;67(22):20100–17. doi: 10.1021/acs.jmedchem.4c01337 (PMC11613437; doi:10.1021/acs.jmedchem.4c01337)

WL_NSC87iso1a

CHEMISTRY
TEXAS A&M UNIVERSITY
Bhuv | X-ray Diffraction Lab

Submitted by: **Wenshe Liu**
Texas A&M University

Solved by: **N Bhuvanesh**

Sample ID: **WLB_SN_240320_SDc_NSC87iso1**

R_1=2.75%

Orig. Sample ID: **NS-C-87 Isomer-1** from **Satyanarayana N.** **Absolute structure unambiguously confirmed. Hooft Parameter = -0.05(4). C10 : S (H10 < C9 < C11 < N1)**

Crystal Data and Experimental

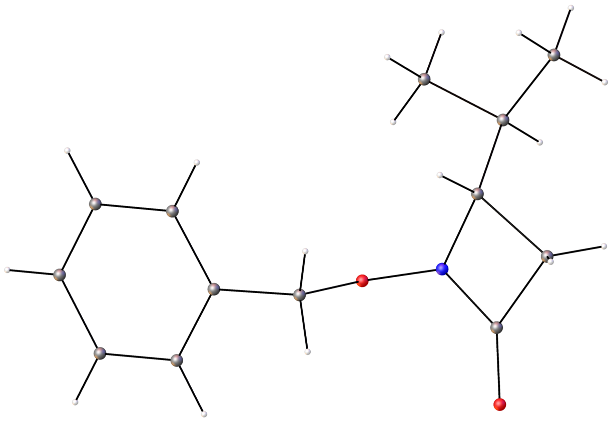

Experimental. Single colourless plate-shaped crystals of **WL_NSC87iso1a** were used as supplied. A suitable crystal with dimensions 0.12 × 0.07 × 0.03 mm³ was selected and mounted on a MITIGEN holder on a XtaLAB Synergy, Dualflex, HyPix diffractometer. The crystal was kept at a steady T = 100.00(10) K during data collection. The structure was solved with the ShelXT (Sheldrick, 2015) solution program using dual methods and by using Olex2 1.5 (Dolomanov et al., 2009) as the graphical interface. The model was refined with ShelXL 2019/1 (Sheldrick, 2015) using full matrix least squares minimisation on F^2.

Crystal Data. $C_{13}H_{17}NO_2$, M_r = 219.27, orthorhombic, $P2_12_12_1$ (No. 19), a = 5.57030(10) Å, b = 9.88460(10) Å, c = 22.1842(2) Å, $\alpha = \beta = \gamma$ = 90°, V = 1221.47(3) Å³, T = 100.00(10) K, Z = 4, Z' = 1, μ(Cu K_α) = 0.642, 24286 reflections measured, 2633 unique (R_{int} = 0.0390) which were used in all calculations. The final wR_2 was 0.0733 (all data) and R_1 was 0.0275 (I≥2 σ(I)).

**Compound**	**WL_NSC87iso1a**
Formula	$C_{13}H_{17}NO_2$
$D_{calc.}$/ g cm^{-3}	1.192
μ/mm^{-1}	0.642
Formula Weight	219.27
Colour	colourless
Shape	plate-shaped
Size/mm³	0.12×0.07×0.03
T/K	100.00(10)
Crystal System	orthorhombic
Flack Parameter	-0.04(7)
Hooft Parameter	-0.05(4)
Space Group	$P2_12_12_1$
a/Å	5.57030(10)
b/Å	9.88460(10)
c/Å	22.1842(2)
α/°	90
β/°	90
γ/°	90
V/Å³	1221.47(3)
Z	4
Z'	1
Wavelength/Å	1.54184
Radiation type	Cu K_α
Θ_{min}/°	3.985
Θ_{max}/°	79.611
Measured Refl's.	24286
Indep't Refl's	2633
Refl's I≥2 σ(I)	2588
R_{int}	0.0390
Parameters	147
Restraints	0
Largest Peak	0.119
Deepest Hole	-0.132
GooF	1.080
wR_2 (all data)	0.0733
wR_2	0.0730
R_1 (all data)	0.0279
R_1	0.0275

Structure Quality Indicators

**Reflections:**	d min (CuKα) 2Θ=159.2° 0.78	I/σ(I) 56.2	Rint m=9.25 3.90%	Full 135.4° 100	
**Refinement:**	Shift 0.001	Max Peak 0.1	Min Peak -0.1	GooF 1.080	Hooft -.05(4)

Experimental (Long Form)

Data Collection

A Leica M80 microscope was used to identify a suitable single **colourless plate-shaped** crystal of **WL_NSC87iso1a** showing well defined faces with dimensions 0.12 × 0.07 × 0.03 mm^3 from a representative sample of crystals of the same habit. The crystal mounted on a nylon loop was then placed in a cold nitrogen stream (Oxford) maintained at T = 100.00(10) K.

Crystal screening, unit cell determination, and data collection were carried out using a XtaLAB Synergy, Dualflex, HyPix diffractometer. The diffraction pattern was indexed and the total number of runs and images was based on the strategy calculation from the program CrysAlisPro system (CCD 43.107a 64-bit (release 07-02-2024)). Data were measured using ω scans with Cu K_{α} radiation. Data was collected to a maximum resolution of Θ = 79.611° (0.78 Å). The unit cell was refined using CrysAlisPro 1.171.43.98a (Rigaku OD, 2023) on 16697 reflections, 69 % of the observed reflections.

Data Reduction, Structure Solution, and Refinement

Integrated Intensity information for each reflection was obtained by reduction of data frames using CrysAlisPro 1.171.43.98a (Rigaku OD, 2023). The final completeness is 100.00 % out to 79.611° in Θ. A gaussian absorption correction was performed using CrysAlisPro 1.171.43.98a (Rigaku Oxford Diffraction, 2023) Numerical absorption correction based on gaussian integration over a multifaceted crystal model Empirical absorption correction using spherical harmonics, implemented in SCALE3 ABSPACK scaling algorithm.. The absorption coefficient μ of this material is 0.642 mm^{-1} at this wavelength (λ = 1.54184Å) and the minimum and maximum transmissions are 0.873 and 1.000.

Systematic reflection conditions and statistical tests of the data suggested the space group $P2_12_12_1$ (# 19) and was confirmed by ShelXT (Sheldrick, 2015) structure solution program using dual methods. The structure was refined by full matrix least squares minimisation on F^2 using version 2019/1 of ShelXL 2019/1 (Sheldrick, 2015). All non-hydrogen atoms were refined anisotropically. Hydrogen atom positions were calculated geometrically and refined using the riding model.

_refine_special_details: Several crystals were tried. The quality of the crystals was not very good. The best among them was chosen and the data was collected. Strategy was targeted for chiral monoclinic to obtain high redundancy. Absolute structure unambiguously confirmed with Hooft parameter = -0.05(4). C10 : S (H10 < C9 < C11 < N1)

_exptl_absorpt_process_details: CrysAlisPro 1.171.43.98a (Rigaku Oxford Diffraction, 2023) Numerical absorption correction based on gaussian integration over a multifaceted crystal model Empirical absorption correction using spherical harmonics, implemented in SCALE3 ABSPACK scaling algorithm.

There is a single molecule in the asymmetric unit, which is represented by the reported sum formula. In other words: Z is 4 and Z' is 1.

The Flack parameter was refined to -0.04(7). Determination of absolute structure using Bayesian statistics on Bijvoet differences using the Olex2 results in -0.05(4). Note: The Flack parameter is used to determine chirality of the crystal studied, the value should be near 0, a value of 1 means that the

stereochemistry is wrong, and the model should be inverted. A value of 0.5 means that the crystal consists of a racemic mixture of the two enantiomers.

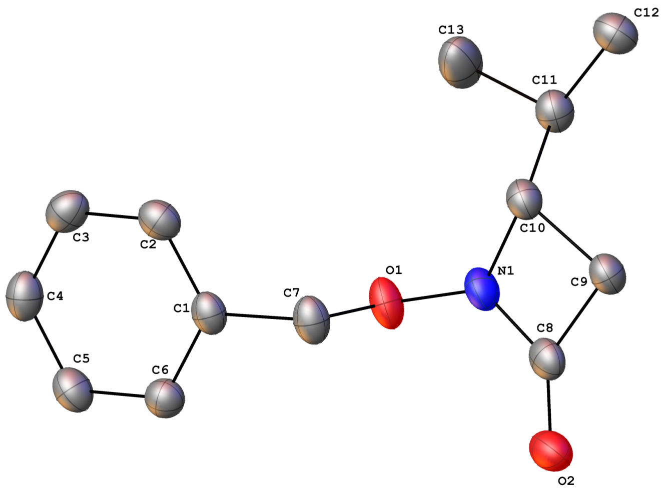

Figure 1 Thermal ellipsoids plot (50 % probability) of WL_NSC87iso1a. Hydrogen atoms are omitted for clarity.

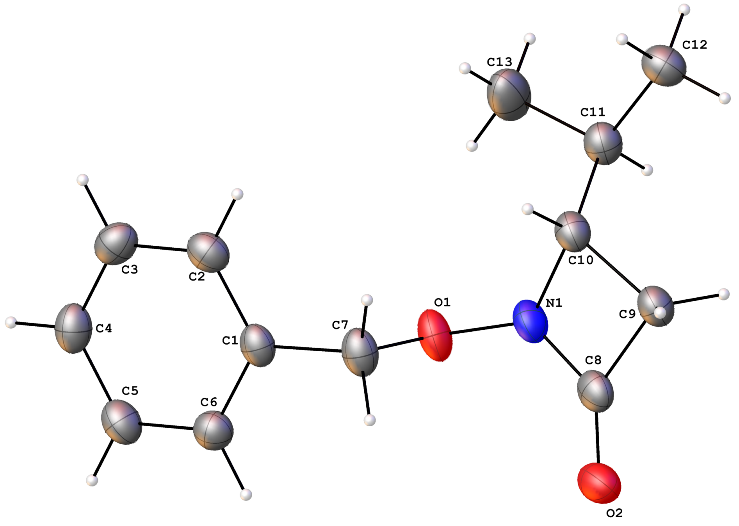

Figure 2 Thermal ellipsoids plot (50 % probability) of WL_NSC87iso1a. Hydrogen atoms are shown at an arbitrarily chosen small radius, and not labeled, for clarity.

Reflection Statistics

Total reflections (after filtering)	24362	Unique reflections	2633
Completeness	0.993	Mean I/σ	46.1
hkl_{max} collected	(5, 12, 28)	hkl_{min} collected	(-7, -12, -27)
hkl_{max} used	(7, 12, 28)	hkl_{min} used	(-7, 0, 0)
Lim d_{max} collected	100.0	Lim d_{min} collected	0.77
d_{max} used	11.09	d_{min} used	0.78
Friedel pairs	1768	Friedel pairs merged	0
Inconsistent equivalents	3	R_{int}	0.039

R_{sigma}	0.0178	Intensity transformed	0
Omitted reflections	0	Omitted by user (OMIT hkl)	0
Multiplicity	(1660, 1332, 1043, 864, 611, 485, 391, 193, 129, 89, 39, 35, 16, 6, 1)	Maximum multiplicity	26
Removed systematic absences	76	Filtered off (Shel/OMIT)	0

Table 1: Fractional Atomic Coordinates (×10^4) and Equivalent Isotropic Displacement Parameters (Å^2×10^3) for **WL_NSC87iso1a**. U_{eq} is defined as 1/3 of the trace of the orthogonalised U_{ij}.

Atom	x	y	z	U_{eq}
O(1)	7975.1(19)	4254.0(9)	6336.7(5)	33.3(2)
O(2)	11119(2)	4940.4(10)	7438.6(5)	37.9(3)
N(1)	8030(2)	5341.3(11)	6734.3(6)	30.5(3)
C(8)	9374(3)	5518.9(13)	7238.8(6)	29.1(3)
C(1)	6197(3)	2131.0(13)	6112.3(6)	27.1(3)
C(6)	7889(3)	1133.7(14)	5989.9(6)	29.4(3)
C(10)	6121(3)	6329.4(13)	6886.1(6)	28.0(3)
C(2)	4151(3)	2223.0(14)	5756.4(6)	30.3(3)
C(9)	7737(3)	6682.1(13)	7433.3(6)	28.9(3)
C(5)	7568(3)	258.0(14)	5507.6(7)	32.3(3)
C(3)	3820(3)	1344.2(16)	5274.0(6)	33.1(3)
C(4)	5533(3)	370.0(15)	5148.6(6)	33.2(3)
C(11)	5649(3)	7407.3(14)	6413.8(6)	30.7(3)
C(7)	6615(3)	3141.6(14)	6604.2(6)	31.9(3)
C(12)	4032(3)	8508.4(15)	6671.9(7)	34.4(3)
C(13)	4528(4)	6772.7(18)	5852.4(8)	49.4(5)

Table 2: Anisotropic Displacement Parameters (×10^4) for **WL_NSC87iso1a**. The anisotropic displacement factor exponent takes the form: $-2\pi^2[h^2a^{*2} \times U_{11} + ... + 2hka^* \times b^* \times U_{12}]$

Atom	U_{11}	U_{22}	U_{33}	U_{23}	U_{13}	U_{12}
O(1)	44.7(6)	22.2(4)	33.0(5)	-6.0(4)	10.5(5)	-5.9(4)
O(2)	33.5(5)	28.4(5)	51.9(6)	3.4(4)	-1.5(5)	1.8(4)
N(1)	33.4(6)	22.6(5)	35.4(6)	-6.8(4)	3.3(5)	0.9(4)
C(8)	29.0(7)	21.8(6)	36.4(7)	2.0(5)	4.0(6)	-4.0(5)
C(1)	34.9(7)	21.6(6)	24.7(6)	1.1(5)	3.8(5)	-3.2(5)
C(6)	31.3(7)	27.9(6)	29.0(6)	2.3(5)	0.1(5)	-0.5(5)
C(10)	28.8(7)	23.7(6)	31.5(6)	-2.4(5)	3.1(5)	-0.9(5)
C(2)	33.7(7)	27.3(6)	30.0(6)	4.6(5)	4.0(6)	1.7(6)
C(9)	33.8(7)	22.7(6)	30.2(6)	-1.3(5)	1.3(6)	0.5(5)
C(5)	36.5(7)	26.9(6)	33.7(7)	-1.3(5)	7.1(6)	1.1(6)
C(3)	34.0(7)	37.4(7)	27.9(6)	5.1(5)	-3.0(5)	-4.7(6)
C(4)	41.6(8)	31.3(7)	26.9(6)	-3.0(5)	3.4(6)	-8.2(6)
C(11)	36.0(7)	27.6(6)	28.6(6)	-0.6(5)	0.7(6)	-1.5(6)
C(7)	43.2(8)	25.6(6)	27.0(6)	-0.5(5)	4.8(5)	-6.0(6)
C(12)	36.8(8)	30.7(7)	35.8(7)	3.2(6)	1.4(6)	4.0(6)
C(13)	73.8(13)	37.6(8)	36.6(8)	-3.3(7)	-14.6(8)	0.4(8)

Table 3: Bond Lengths in Å for **WL_NSC87iso1a**.

Atom	Atom	Length/Å
O(1)	N(1)	1.3907(14)
O(1)	C(7)	1.4612(16)
O(2)	C(8)	1.2116(18)
N(1)	C(8)	1.3578(19)
N(1)	C(10)	1.4827(17)
C(8)	C(9)	1.5297(19)
C(1)	C(6)	1.390(2)
C(1)	C(2)	1.390(2)
C(1)	C(7)	1.4977(18)
C(6)	C(5)	1.388(2)
C(10)	C(9)	1.5509(19)
C(10)	C(11)	1.5173(19)

Atom	Atom	Length/Å
C(2)	C(3)	1.391(2)
C(5)	C(4)	1.390(2)
C(3)	C(4)	1.384(2)
C(11)	C(12)	1.524(2)
C(11)	C(13)	1.528(2)

Table 4: Bond Angles in ° for **WL_NSC87iso1a**.

Atom	Atom	Atom	Angle/°
N(1)	O(1)	C(7)	109.60(10)
O(1)	N(1)	C(10)	129.59(12)
C(8)	N(1)	O(1)	129.41(12)
C(8)	N(1)	C(10)	97.07(11)
O(2)	C(8)	N(1)	133.06(14)
O(2)	C(8)	C(9)	136.87(14)
N(1)	C(8)	C(9)	90.05(11)
C(6)	C(1)	C(7)	120.65(13)
C(2)	C(1)	C(6)	119.43(12)
C(2)	C(1)	C(7)	119.86(13)
C(5)	C(6)	C(1)	120.37(13)
N(1)	C(10)	C(9)	84.81(10)
N(1)	C(10)	C(11)	115.47(11)
C(11)	C(10)	C(9)	118.90(11)
C(1)	C(2)	C(3)	120.33(13)
C(8)	C(9)	C(10)	87.50(10)
C(6)	C(5)	C(4)	119.81(13)
C(4)	C(3)	C(2)	119.88(14)
C(3)	C(4)	C(5)	120.17(13)
C(10)	C(11)	C(12)	110.15(11)
C(10)	C(11)	C(13)	110.21(12)
C(12)	C(11)	C(13)	110.98(14)
O(1)	C(7)	C(1)	106.65(11)

Table 5: Torsion Angles in ° for **WL_NSC87iso1a**.

Atom	Atom	Atom	Atom	Angle/°
O(1)	N(1)	C(8)	O(2)	13.7(3)
O(1)	N(1)	C(8)	C(9)	-164.92(13)
O(1)	N(1)	C(10)	C(9)	164.80(13)
O(1)	N(1)	C(10)	C(11)	-75.59(17)
O(2)	C(8)	C(9)	C(10)	-172.84(17)
N(1)	O(1)	C(7)	C(1)	168.65(11)
N(1)	C(8)	C(9)	C(10)	5.69(10)
N(1)	C(10)	C(9)	C(8)	-5.23(9)
N(1)	C(10)	C(11)	C(12)	-168.76(12)
N(1)	C(10)	C(11)	C(13)	68.46(18)
C(8)	N(1)	C(10)	C(9)	5.94(10)
C(8)	N(1)	C(10)	C(11)	125.55(12)
C(1)	C(6)	C(5)	C(4)	-0.7(2)
C(1)	C(2)	C(3)	C(4)	0.2(2)
C(6)	C(1)	C(2)	C(3)	-1.4(2)
C(6)	C(1)	C(7)	O(1)	87.00(15)
C(6)	C(5)	C(4)	C(3)	-0.5(2)
C(10)	N(1)	C(8)	O(2)	172.63(15)
C(10)	N(1)	C(8)	C(9)	-6.00(11)
C(2)	C(1)	C(6)	C(5)	1.6(2)
C(2)	C(1)	C(7)	O(1)	-90.18(16)
C(2)	C(3)	C(4)	C(5)	0.7(2)
C(9)	C(10)	C(11)	C(12)	-70.22(16)
C(9)	C(10)	C(11)	C(13)	166.99(14)
C(11)	C(10)	C(9)	C(8)	-121.54(13)
C(7)	O(1)	N(1)	C(8)	76.94(17)
C(7)	O(1)	N(1)	C(10)	-75.47(17)
C(7)	C(1)	C(6)	C(5)	-175.61(12)
C(7)	C(1)	C(2)	C(3)	175.85(12)

Table 6: Hydrogen Fractional Atomic Coordinates (×10^4) and Equivalent Isotropic Displacement Parameters (Å^2×10^3) for **WL_NSC87iso1a**. U_{eq} is defined as 1/3 of the trace of the orthogonalised U_{ij}.

Atom	x	y	z	U_{eq}
H(6)	9271.34	1051.16	6237.88	35
H(10)	4606.15	5868.63	7014.21	34
H(2)	2971.79	2889.56	5843.04	36
H(9A)	8496.53	7585.86	7408.73	35
H(9B)	6955.24	6542.65	7829.24	35
H(5)	8736.28	-415.97	5423.03	39
H(3)	2421.35	1412.4	5031.02	40
H(4)	5317.8	-223.77	4816.23	40
H(11)	7216.26	7825.96	6297.87	37
H(7A)	5065.91	3474.11	6765.07	38
H(7B)	7534.37	2725.43	6938.14	38
H(12A)	2491.46	8111.98	6791.24	52
H(12B)	3760.47	9206.02	6365.52	52
H(12C)	4806.03	8915.16	7024.82	52
H(13A)	5612.21	6081.68	5690.39	74
H(13B)	4262.89	7474.64	5547.61	74
H(13C)	2990.29	6354.7	5959.04	74

Citations

CrysAlisPro (ROD), Rigaku Oxford Diffraction, Poland (?).

CrysAlisPro Software System, Rigaku Oxford Diffraction, (2023).

O.V. Dolomanov and L.J. Bourhis and R.J. Gildea and J.A.K. Howard and H. Puschmann, Olex2: A complete structure solution, refinement and analysis program, *J. Appl. Cryst.*, (2009), **42**, 339-341.

Sheldrick, G.M., Crystal structure refinement with ShelXL, *Acta Cryst.*, (2015), **C71**, 3-8.

Sheldrick, G.M., ShelXT-Integrated space-group and crystal-structure determination, *Acta Cryst.*, (2015), **A71**, 3-8.

checkCIF/PLATON report

Structure factors have been supplied for datablock(s) wl_nsc87iso1a

THIS REPORT IS FOR GUIDANCE ONLY. IF USED AS PART OF A REVIEW PROCEDURE FOR PUBLICATION, IT SHOULD NOT REPLACE THE EXPERTISE OF AN EXPERIENCED CRYSTALLOGRAPHIC REFEREE.

No syntax errors found. CIF dictionary Interpreting this report

Datablock: wl_nsc87iso1a

```
Bond precision:   C-C = 0.0020 A          Wavelength=1.54184

Cell:              a=5.5703(1)       b=9.8846(1)       c=22.1842(2)
                   alpha=90          beta=90           gamma=90
Temperature:       100 K
```

	Calculated	Reported
Volume	1221.47(3)	1221.47(3)
Space group	P 21 21 21	P 21 21 21
Hall group	P 2ac 2ab	P 2ac 2ab
Moiety formula	C13 H17 N O2	C13 H17 N O2
Sum formula	C13 H17 N O2	C13 H17 N O2
Mr	219.28	219.27
Dx,g cm-3	1.192	1.192
Z	4	4
Mu (mm-1)	0.642	0.642
F000	472.0	472.0
F000'	473.39	
h,k,lmax	7,12,28	7,12,28
Nref	2652[1568]	2633
Tmin,Tmax	0.948,0.984	0.873,1.000
Tmin'	0.923	

```
Correction method= # Reported T Limits: Tmin=0.873 Tmax=1.000
AbsCorr = GAUSSIAN

Data completeness= 1.68/0.99       Theta(max)= 79.611

R(reflections)= 0.0275( 2588)                    wR2(reflections)= 0.0733( 2633)

S = 1.080                 Npar= 147
```

The following ALERTS were generated. Each ALERT has the format
test-name_ALERT_alert-type_alert-level.
Click on the hyperlinks for more details of the test.

Alert level G

```
PLAT143_ALERT_4_G s.u. on c - Axis Small or Missing .............    0.00020 Ang.
PLAT395_ALERT_2_G Deviating  X-O-Y   Angle From 120 for O1       .      109.6 Degree
PLAT912_ALERT_4_G Missing # of FCF Reflections Above STh/L=  0.600          6 Note
PLAT969_ALERT_5_G The 'Henn et al.' R-Factor-gap value ..........        3.94 Note
              Predicted wR2: Based on SigI**2  1.86 or SHELX Weight  6.98
PLAT978_ALERT_2_G Number C-C Bonds with Positive Residual Density.          3 Info
```

0 **ALERT level A** = Most likely a serious problem - resolve or explain
0 **ALERT level B** = A potentially serious problem, consider carefully
0 **ALERT level C** = Check. Ensure it is not caused by an omission or oversight
5 **ALERT level G** = General information/check it is not something unexpected

0 ALERT type 1 CIF construction/syntax error, inconsistent or missing data
2 ALERT type 2 Indicator that the structure model may be wrong or deficient
0 ALERT type 3 Indicator that the structure quality may be low
2 ALERT type 4 Improvement, methodology, query or suggestion
1 ALERT type 5 Informative message, check

It is advisable to attempt to resolve as many as possible of the alerts in all categories. Often the minor alerts point to easily fixed oversights, errors and omissions in your CIF or refinement strategy, so attention to these fine details can be worthwhile. In order to resolve some of the more serious problems it may be necessary to carry out additional measurements or structure refinements. However, the purpose of your study may justify the reported deviations and the more serious of these should normally be commented upon in the discussion or experimental section of a paper or in the "special_details" fields of the CIF. checkCIF was carefully designed to identify outliers and unusual parameters, but every test has its limitations and alerts that are not important in a particular case may appear. Conversely, the absence of alerts does not guarantee there are no aspects of the results needing attention. It is up to the individual to critically assess their own results and, if necessary, seek expert advice.

Publication of your CIF in IUCr journals

A basic structural check has been run on your CIF. These basic checks will be run on all CIFs submitted for publication in IUCr journals (*Acta Crystallographica*, *Journal of Applied Crystallography*, *Journal of Synchrotron Radiation*); however, if you intend to submit to *Acta Crystallographica Section C* or *E* or *IUCrData*, you should make sure that full publication checks are run on the final version of your CIF prior to submission.

Publication of your CIF in other journals

Please refer to the *Notes for Authors* of the relevant journal for any special instructions relating to CIF submission.

PLATON version of 06/01/2024; check.def file version of 05/01/2024

Datablock wl_nsc87iso1a - ellipsoid plot

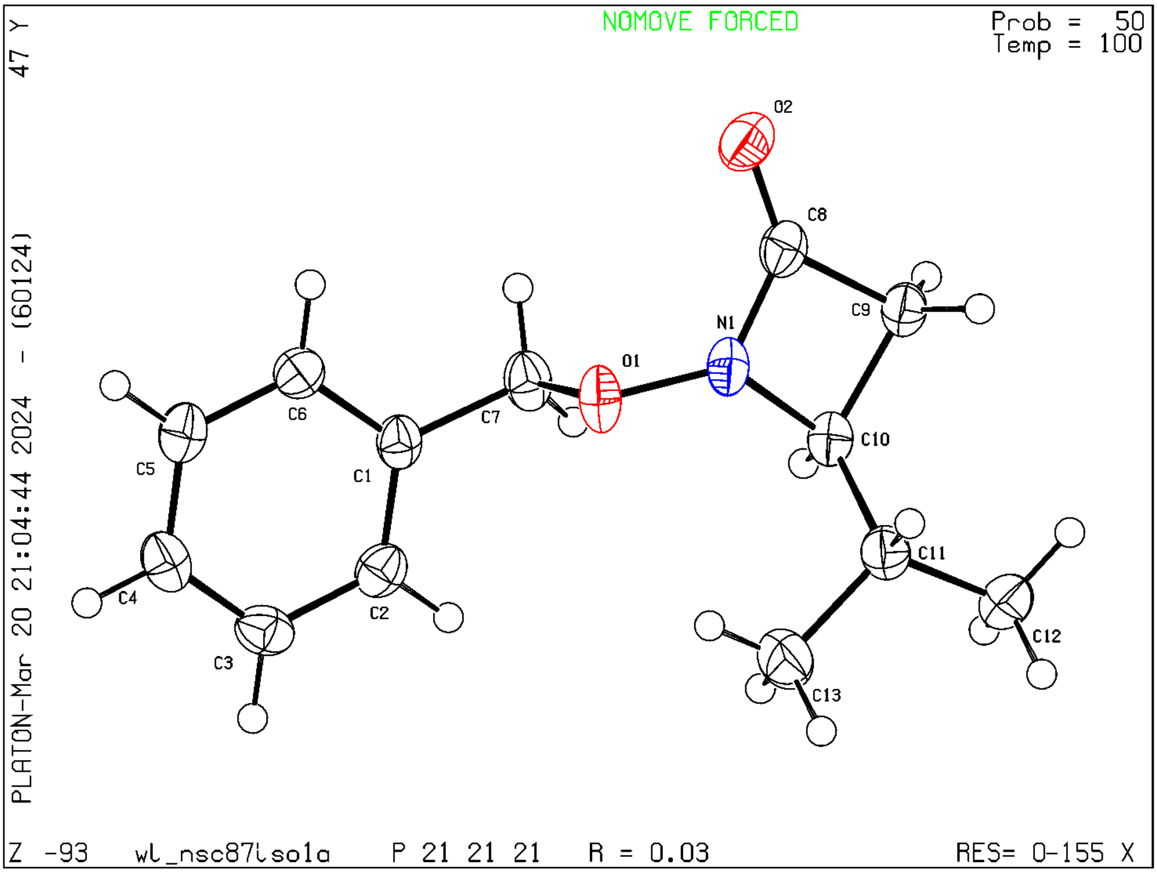

Supplement: Supplementary file 3 — jm4c01337_si_003.pdf [file jm4c01337_si_003.pdf]
